# Rare twin cysteine residues in the HIV-1 envelope variable region 1 link to neutralization escape and breadth development

**DOI:** 10.1016/j.chom.2025.01.004

**Published:** 2025-02-04

**Authors:** Maria C. Hesselman, Marius Zeeb, Peter Rusert, Chloé Pasin, Jennifer Mamrosh, Samuel Kariuki, Ian Pichler, Michèle Sickmann, Masako M. Kaufmann, Daniel Schmidt, Nikolas Friedrich, Karin J. Metzner, Audrey Rindler, Herbert Kuster, Craig Adams, Ruwayhida Thebus, Michael Huber, Sabine Yerly, Karoline Leuzinger, Matthieu Perreau, Roger Koller, Günter Dollenmaier, Simona Frigerio, Dylan H. Westfall, Wenjie Deng, Allan C. deCamp, Michal Juraska, Srilatha Edupuganti, Nyaradzo Mgodi, Hugh Murrell, Nigel Garrett, Kshitij Wagh, James I. Mullins, Carolyn Williamson, Penny L. Moore, Huldrych F. Günthard, Roger D. Kouyos, Alexandra Trkola

**Affiliations:** 1 Institute of Medical Virology, University of Zurich (UZH), 8057 Zurich, Switzerland; 2 Department of Infectious Diseases and Hospital Epidemiology, University Hospital Zurich (USZ), 8091 Zurich, Switzerland; 3 Theoretical Biology and Biophysics Group, Los Alamos National Laboratory, Los Alamos, NM 87545, USA; 4 Department of Biological Sciences, School of Science, University of Eldoret, 30100 Eldoret, Kenya; 5 Institute for Infectious Diseases and Molecular Medicine, Division of Medical Virology, Faculty of Health Sciences, University of Cape Town and National Health Laboratory Service, 7925 Cape Town, South Africa; 6 Laboratory of Virology, University Hospital Geneva, University of Geneva, 1205 Geneva, Switzerland; 7 Clinical Virology, University Hospital Basel, 4031 Basel, Switzerland; 8 Division of Immunology and Allergy, University Hospital Lausanne, University of Lausanne, 1011 Lausanne, Switzerland; 9 Institute for Infectious Diseases, University of Bern, 3001 Bern, Switzerland; 10 Centre for Laboratory Medicine, 9008 St. Gallen, Switzerland; 11 Institute of Laboratory Medicine, Ente Ospedaliero Cantonale, 6500 Bellinzona, Switzerland; 12 Department of Microbiology at the University of Washington, Seattle, WA 98195, USA; 13 Fred Hutchinson Cancer Center, Seattle, WA 98109, USA; 14 Department of Medicine, Division of Infectious Diseases, Emory University, Atlanta, GA 30322, USA; 15 University of Zimbabwe Clinical Trials Research Centre, Harare, Zimbabwe; 16 University of California, San Francisco, San Francisco, CA 94115, USA; 17 Department of Public Health Medicine, School of Nursing and Public Health, University of KwaZulu-Natal, 4041 Durban, South Africa; 18 SA MRC Antibody Immunity Research Unit, School of Pathology, Faculty of Health Sciences, University of the Witwatersrand, 2050 Johannesburg, South Africa; 19 National Institute for Communicable Disease of the National Health Laboratory Services, 2192 Johannesburg, South Africa; 20 Centre for the AIDS Programme of Research in South Africa (CAPRISA), University of KwaZulu-Natal, 4013 Durban, South Africa; 21 Lead contact

## Abstract

Identifying HIV-1 envelope (Env) traits associated with neutralization cross-reactivity is crucial for vaccine design. Variable loops 1 and 2 (V1V2), positioned at the Env trimer apex, are key regions linked to neutralization. We describe non-canonical cysteine (Cys) residues in V1 that are enriched in individuals with elite neutralization breadth. Analyzing over 65,000 V1 sequences from the CATNAP database, AMP trials, and longitudinal HIV-1 cohorts (SHCS, ZPHI, and CAPRISA), we found that Env variants with extra V1 Cys are present at low levels and fluctuate over time. Extra V1 Cys associate with elite plasma neutralization, and two additional Cys are preferred, suggesting stabilization through disulfide bonds. Among 34 broadly neutralizing antibody (bnAb)-inducer Envs, 17.6% had elongated V1 regions with extra Cys. These extra Cys moderately increased neutralization resistance and altered bnAb epitope accessibility. Collectively, altering epitope exposure alongside Env stabilization renders the V1 twin Cys motif a promising feature for HIV-1 bnAb immunogens.

## In brief

Hesselman et al. identify rare non-canonical cysteines in the HIV-1 Env V1 region that are associated with elite neutralization development and contribute to bnAb escape. Twin V1 Cys residues decrease epitope exposure for most bnAbs but increase accessibility for V2-apex bnAbs, highlighting their potential in creating effective HIV-1 immunogens.

## Graphical Abstract

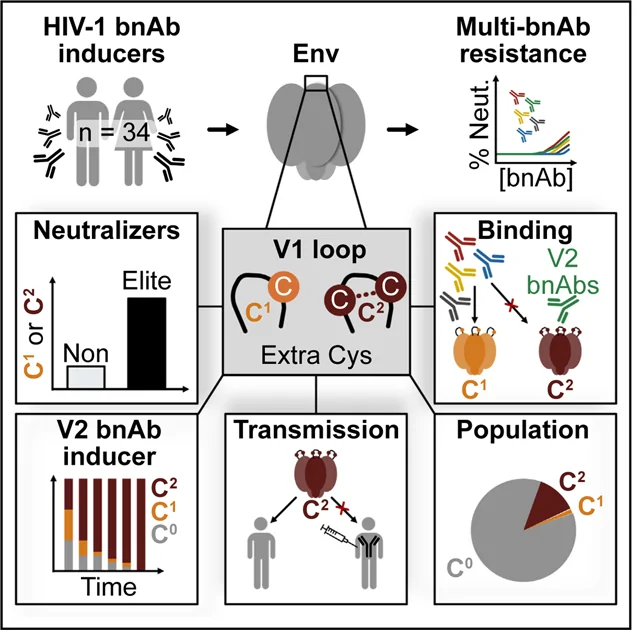

## INTRODUCTION

Genetic traits of the HIV-1 envelope glycoprotein (Env) that link to bnAb development and escape in untreated HIV-1 infection are key to vaccine immunogen development. A known regulator of neutralization resistance is the Env region encompassing variable loops 1 and 2 (V1V2), which affects the neutralization properties of multiple epitopes, including those of bnAbs.^1–11^ Spanning residues 126 to 196 in the gp120 portion of Env (HXB2 numbering), the V1V2 domain is positioned at the apex of the Env trimer.^12–14^ There, it forms a top layer on the trimer that shields key neutralization-sensitive sites, the variable loop 3 (V3), and the co-receptor-binding site.

The high degree of sequence and length heterogeneity observed in the V1V2 is indicative of its remarkable adaptability.^1–10,15^ Longer and more glycosylated V1V2 can decrease susceptibility to many bnAbs.^1,4,11^ Furthermore, V1V2 length dynamics during infection align with the presence or absence of neutralizing antibodies (nAbs).^1,3^ During sexual transmission of HIV-1, a robust genetic bottleneck allows only a few transmitted variants to establish the infection,^16–19^ and in some transmission cohorts, shorter and less-glycosylated V1V2 regions appeared to be favored during transmission and early infection.^3,20^ The onset of humoral immunity and nAb development sees an elongation of the variable loops, which subsides with waning humoral immunity in late infection stages.^3^ Although longer V1V2 regions may provide an advantage in the presence of nAbs, the shortening of the loops in environments where nAbs are absent highlights the potential detrimental effect of V1V2 elongation on viral fitness.

We previously conducted the Swiss 4.5K screen,^21,22^ a population-level investigation of bnAb activity in 4,484 people with HIV-1 (PWH) participating in the Swiss HIV Cohort Study (SHCS)^23,24^ and the Zurich Primary Infection Study (ZPHI).^25^ The Swiss 4.5K screen led to the definition of key parameters of bnAb induction and identified 239 bnAb inducers.^21,22^ This screen and a follow-up study investigating 303 transmission pairs revealed a clear effect of virus genetics on the antibody response.^26^ Here, building on these findings, we conducted a search for *env* genetic and phenotypic features that are enriched among bnAb inducers.

## RESULTS

### High neutralization resistance among Envs from bnAb inducers

To gain insight into global Env features associated with bnAb induction and escape, we leveraged a collection of bnAb-inducer samples and data identified in the Swiss 4.5K screen.^21,22^ We created a panel of *env* cloned from plasma virus of 34 bnAb inducers with diverse neutralizing specificities and high breadth and potency (Figures 1A and S1A; Table S1). The bnAb-inducer pseudovirus (PSV) panel was significantly more neutralization resistant to 14/19 reference bnAbs when compared with a previously described^21^ multi-subtype panel of Envs (Wilcoxon *p* < 0.05) (*n* = 41; Tables S2–S4) (Figures S1B and S1C). We expected to observe epitope-specific bnAb resistance, corresponding to *in vivo* escape from the bnAb specificity in the donor. Instead, we observed a remarkable generalized resistance of bnAb-inducer Envs to neutralization, with the majority of bnAb-virus combinations having 50% inhibitory concentrations (IC50) >1 μg/mL (Figure 1B; Table S2). Notably, four bnAbs in clinical development, VRC01, 3BNC117, PGT121, and PGDM1400, all showed poor neutralization breadth against bnAb-inducer Envs. Only the CD4-binding site (CD4bs) bnAb N6, the membrane proximal region (MPER) bnAbs DH511.11P and 10E8, and the silent face (SF) bnAb SF12, retained high breadth against the resistant bnAb-inducer Envs, neutralizing more than 50% of Envs with IC50s ≤ 1 μg/mL, which was not the case for the 41-virus panel (Figure 1B). Resistance to V3-glycan and V2-apex bnAbs was particularly high, despite similar frequencies of key glycosylation sites for V3-glycan (N332/N334) and V2-apex (N156/N160) bnAbs among both panels (Tables S2 and S4).

Because the Envs in the bnAb-inducer PSV panel were all derived from chronic infection, we compared the bnAb-inducer Envs to a separate set of 123 chronic infection Envs derived from the CATNAP^27^ database (Table S5), confirming that bnAb-inducer Envs were more neutralization resistant than average chronic Envs (Figure 1C). Setting IC50 < 1 μg/mL as a potency threshold, bnAbs showed markedly lower breadth against bnAb-inducer Envs compared with CATNAP-chronic Envs (Figure 1D, t test *p* < 0.0001). Ten out of 19 bnAbs tested were significantly less potent against the bnAb-inducer Env panel than CATNAP-chronic Envs (Wilcoxon *p* values < 0.0001 to 0.03) (Figures 1C, S1D, and S1E) and several more bnAbs (CH01, PGDM12, PGDM21, and PGT151) with overall lower breadth showed the same trend without reaching significance. Only the MPER bnAbs, the SF bnAb SF12, and the interface bnAb 35O22 had similar potency against bnAb-inducer Envs (Figures 1C, S1D, and S1E). Because the bnAb-inducer panel was enriched for subtype B viruses (22/34; Table S2), we conducted a subtype-B-restricted comparison that confirmed its higher resistance compared with CATNAP-Envs of subtype B (Figure S1E).

Collectively, we conclude that bnAb-inducer Envs have a marked generalized neutralization resistance against various bnAbs that is not subtype specific and extends beyond bnAb-specific escape.

### Distinct features of the V1 loop among bnAb-inducer Envs

Env variable loop length and glycosylation can act as a regulator of neutralization sensitivity.^1–10^ We observed significantly longer V1, V2, and V5 loops in bnAb-inducer Envs as compared with CATNAP-chronic Envs (Figure 2A). The largest difference was in V1 (mean length 35 vs. 29 residues, t test *p* < 0.0001) (Figure 2A). The differences in V2 and V5 length were less pronounced, though statistically significant, whereas V3 and V4 lengths were not significantly different between the bnAb-inducer and CATNAP-chronic Env panels (Figure 2A). Furthermore, bnAb-inducer Envs had on average more glycosylation sites in V1, V2, and V3 loops than CATNAP-chronic Envs, and V4 and V5 loops had fewer glycosylation sites (t test *p* < 0.05) (Figure 2B).

In the closed Env trimer conformation, the V1V2 region forms a Greek key motif held together by disulfide bonds between two pairs of highly conserved cysteine (Cys) residues,^28,29^ one in V1 between residues 131 and 157 and a second in V2 between residues 126 and 196.^30^ Analysis of the V1 regions of the bnAb-inducer Env panel showed that 6/34 bnAb-inducer Envs (17.6%) had non-canonical Cys residues in their V1 loops in addition to the conserved Cys at 131 and 157 (Figures 2C and S1F). Insertions of two Cys residues (C^2^) were preferred over single Cys insertions (C^1^), with five of the bnAb-inducer Envs bearing C^2^, whereas only one had C^1^. The five Envs with C^2^ had especially long V1 regions, averaging 38 residues compared with an average of 29-residue-long V1 regions among CATNAP-chronic Envs. C^1^ and C^2^ Envs were of different subtypes (B, CPX, and D) and from donors with different predicted bnAb neutralization specificities (V3-glycan, CD4bs, V2-apex, and MPER), with 3/6 stemming from V3-glycan bnAb-activity-predicted donors (Figure 2C).

The presence of C^2^ suggests that an additional disulfide bond may form in these Envs, which may contribute to the conformational stability of the region. Elongated V1 loops with C^2^ have been reported thus far in only a few donors, notably including seven cases of bnAb inducers.^6,31–33^ Taken together, the observation of V1 Cys insertions in bnAb-inducer Envs by us and others suggests that V1 Cys insertions may be associated with bnAb evolution.

### Cys insertions in V1 promote low-level neutralization resistance

We hypothesized that stabilization of the V1 domain through an additional disulfide bond may contribute to the high degree of generalized neutralization resistance in some bnAb-inducer Envs. To investigate this, we followed plasma neutralization and *env* evolution longitudinally in donor S5206G5, an elite neutralizer with a predicted V2-apex bnAb specificity (Table S6).^21^ The S5206G5 Env (S5206G5–581wk-c51) included in the bnAb-inducer Env panel combines the features we observed: a long V1 region (37 residues), including a C^2^ motif and high resistance to a wide range of bnAb types (Figure 2C; Table S2). S5206G5 plasma neutralization breadth against a 19-virus multiclade panel peaked at 90% in week 610 post estimated date of infection (EDI) and reached a maximum geometric mean 50% neutralization titer (NT50) of 1:6,275 at week 643 (Figure S2A). Sequence analysis of *env* cloned from plasma viruses at 12 time points, and *env* haplotypes reconstructed from Nanopore sequencing at eight time points, revealed that variants with no extra V1 Cys (C^0^)––C^1^ and C^2^—co-existed at week 42, the earliest available sample (Figures 3A and S2B–S2E). Afterward, C^2^ became predominant and the V1 region longer, suggesting that this motif provided a benefit for the virus (Figure 3A). The peaks in plasma breadth were accompanied by subsequent increases in the fraction of *env* haplotypes containing internal STOP codons (Figure S2D).

To investigate the effect of the C^2^ motif on sensitivity to S5206G5 autologous plasma neutralization, we selected two *env* clones from week 42 that represented naturally occurring C^0^ and C^2^ genotypes and generated mutated versions by either removing or adding extra Cys residues (Figure 3B). With the exception of clone S5206G5–42-wk-c3 C^0^ variant, all variants were entry competent. All Env variants had comparable autologous plasma NT50s (fixed effects model *p* > 0.05, Figure 3C). Both wild-type (WT) and mutant Env variants proved relatively resistant against heterologous bnAb-inducer plasma with different bnAb specificities (Table S6), with only a few of the longitudinal plasma-virus combinations reaching NT50 > 1:100 (Table S7). Comparing percent neutralization at a 1:100 plasma dilution across all longitudinal heterologous plasma profiles, we saw a significantly decreased neutralization of C^2^ variants compared with C^1^/C^0^ variants (Figure 3D) (linear mixed effects model *p* < 0.001). The differences in neutralization susceptibility were subtle, with most being prominent in plasmas with weak neutralization activity, such as S30834, S51928, S50725, S5213G5, and S10437, against Env S5206G5–42wk-c3.

The mean IC50s of 14 reference bnAbs (Table S3) were not affected by the C^2^ motif (Wilcoxon *p* > 0.05), but bnAbs PG16 and VRC26.25 lost neutralization against the C^2^ variant of S5206G5–42wk-c6.2 (Figure S2F). Similar to plasma inhibition, modest resistance increases between C^0^ and C^2^ inhibition were evident when comparing neutralization at a fixed, low bnAb concentration, although not all bnAbs showed shifts (Figure S2G). These results indicate that although C^2^ in S5206G5 Env has no clear impact on potent, high-dose bnAb-mediated neutralization, the C^2^ motif reduces neutralizing sensitivity under conditions such as lower bnAb doses or in the presence of weaker neutralizing plasma and antibodies.

We then harnessed neutralization and sequence data available on CATNAP^27^ for 21 bnAbs and C^0^ (*n* = 1,189) or C^2^ (*n* = 71) *env* sequences. C^2^ Envs were significantly more resistant against bnAbs PG16, PG9, PGT121, and 35O22 (mixed effects model adjusted *p* < 0.05) (Figures 3E and S2H). The higher neutralization resistance of C^2^ Envs in the CATNAP database suggests that C^2^ insertions play a role in resistance development to bnAbs targeting diverse epitopes in diverse Env backgrounds.

### C^2^ motif shifts accessibility of bnAb epitopes

We next hypothesized that C^2^ motifs may facilitate V1-mediated shielding by stabilizing the V1 and generated soluble C^2^ and C^1^ native-like SOSIP and DS-SOSIP trimers (Table S8). Env S5206G5–42wk-c3 naturally carries a C^2^ motif and we made two C^1^ versions of it by mutating either of the two extra V1 Cys to serine (Ser) (C146eS or C137S, HXB2 numbering). Removal of either Cys leads to enhanced binding of many bnAbs to C^1^ DS-SOSIP (Figures 4A and 4B) and C^1^ SOSIP (Figures S3A and S3B) trimers compared with the C^2^ versions, as shown by a binding antibody multiplex assay (BAMA) on 29 reference bnAbs and monoclonal antibodies (mAbs) (Table S3). The difference was significant for CD4bs and interface/fusion peptide bnAbs (Wilcoxon *p* < 0.05) (Figure 4B). mAbs 17b and 48d failed to bind DS-SOSIP trimers but showed equal capacity to bind SOSIP C^1^ and C^2^ variants (Figures S3A–S3F), confirming that the changes in epitope exposure in C^1^ trimers did not result in general trimer opening.

To define the relative accessibility of the bnAb epitopes in a given Env to each other, we normalized the BAMA data to the maximum binding observed for N6 (Figures 4C, 4D, S3C, and S3D). These analyses highlighted an increase in V2-apex bnAb binding to C^2^ compared with C^1^ variants, both by comparing effects on binding of individual bnAbs and by comparing effects on distinct regions (Figure 4D) (Wilcoxon *p* < 0.05). The alterations in epitope exposure from C^2^ to C^1^ were similar, irrespective of which site was removed or whether we analyzed raw or normalized MFI (Figures 4E, 4F, S3E, and S3F), suggesting that both Cys residues contribute equally to the epitope-modulating properties of the C^2^ motif, most likely by forming a disulfide bond.

Taken together, these findings indicate that the C^2^ motif in S5206G5 may have resulted from an escape from early autologous nAb activity, suggesting that by inadvertently exposing the V2, the C^2^ motif may have generated an improved Env antigen that favored the V2-apex bnAb evolution that occurred later in this donor.

### V1 Cys insertions are enriched among Env of bnAb inducers

The high frequency of C^1^ and C^2^ in the bnAb-inducer Env panel, along with the functional data, prompted us to investigate the role of these motifs in the larger SHCS and ZPHI cohorts, which included participants of the Swiss 4.5K screen. We determined the frequency of C^1^/C^2^ among participants using NGS-derived consensus V1 *env* sequences from plasma viruses (*n* = 6,424 sequences from 4,373 unique participants) generated in previous studies^34,35^ and V1 sequences derived from single *env* clones (*n* = 750 from 77 unique participants) (Figure S4A; Table S9). V1 sequences were used to study the frequencies of C^1^/C^2^ in the context of bnAb evolution (Figures 5A and 5B), across infection stages, including early transmission (Figures 5C and S4B) and population-wide time trends (Figure S4C).

For 484 of the 4,484 Swiss 4.5K participants with the required sequence data available (Figure S4A), we quantified the proportion of donors carrying C^1^/C^2^ *env* variants as a function of their bnAb-inducer status as defined in the Swiss 4.5K screen.^21,22^ We observed a significantly higher frequency of C^1^/C^2^ in bnAb inducers, with 6/31 elite neutralizers and only 19/362 non-neutralizers carrying C^1^ or C^2^ Env (19.4% vs. 5.2%, logistic regression, *p* = 0.0042) (Figure 5A). Using univariable and multivariable logistic regression models, we confirmed a positive, independent association between the presence of C^1^/C^2^ and elite bnAb activity (adjusted odds ratio = 6.26, 95% confidence interval 1.89–20.72) (Figure 5B).

We next monitored the frequency of C^1^/C^2^ Envs as a function of duration of untreated HIV-1 infection in 1,528 SHCS and ZPHI donors (Figure S4A). We detected C^1^ and C^2^ variants at all disease stages, including very early after transmission, where 7.5% of participants sequenced <90 days post infection had C^1^ or C^2^ *env* variants (Figure S4B). The frequency of participants with C^1^/C^2^ Envs was constant throughout early and late infection, ranging between 6.2% and 9.9% (logistic regression *p* > 0.05), suggesting that V1 Cys insertion is not restricted to specific disease stages (Figure S4B).

Because extra V1 Cys are associated with increased resistance to neutralization, we probed whether HIV-1 was evolving toward a higher frequency of C^1^/C^2^ at the population level. Analyzing SHCS and ZPHI Env sequences from the first infection year (*n* = 431) sampled between 1996 and 2019, we observed no significant differences in frequencies of participants with C^1^/C^2^ between different sampling years (logistic regression *p* > 0.05) (Figure S4C). In a similar analysis, we examined the Los Alamos HIV Sequence Database^36^ for differences in year of sampling of C^0^, C^1^, and C^2^ sequences among viruses sampled < 1 year post seroconversion. There were no significant differences in sampling year, consistent with the SHCS and ZPHI cohort data (t test *p* > 0.05) (Figure S4D).

Longitudinal sequences were available for 33 SHCS and ZPHI participants with C^1^/C^2^ Envs allowing us to study within-donor temporal dynamics. Across longitudinal samples, C^0^ and C^2^ variants were the most common, with C^1^ and C^3^ (containing three extra V1 Cys) only appearing sporadically (Figure 5C). For 9/33 participants, C^2^ variants were detected at all time points (C^2^ dominant, Figure 5C). Nineteen participants had C^2^ variants appearing transiently and five had transient C^1^ sequences without any C^2^ variants detected. We recorded C^2^ Envs at more than one time point for six bnAb inducers with V3-glycan, CD4bs, and V2-apex plasma specificities (Figure 5C). As these analyses were based on consensus sequences and single Env clones, we cannot exclude the possibility that C^2^ variants persisted as minor variants for prolonged periods. A re-appearance of C^2^ variants is indicative of this scenario in participant S51125 (Figure 5C).

The consensus sequences and *env* clones of the SHCS/ZPHI provided information on whether or not C^1^/C^2^ *env* were present at a given time point but not their frequencies. To obtain further insights into the evolutionary dynamics and proportions of C^1^ and C^2^ *env*, we analyzed longitudinal V1 sequences from 103 women enrolled in the CAPRISA 002 cohort.^37,38^ These sequences were obtained from deep sequencing of plasma virus sampled from very early infection (2 weeks) to 4 years post infection using the Pacific Biosciences SMRT (PacBio-SMRT) UMI approach, which tags each RNA molecule, enabling a proportional analysis of *env* variants.^39,40^ We analyzed an average of 191 sequences per participant at each time point (range 1–893) and found that 20/103 (19.4%) participants had at least one C^1^ or C^2^ sequence at any time point (Figure 5D). C^2^ Envs reached frequencies of >50% in seven donors (C^2^ dominant), one bnAb inducer, and six non-neutralizers (Figure 5E). In the remaining 13 participants, C^0^ Envs dominated, with only transient increases in the frequencies of C^1^ Envs.

Taken together, the low frequency of C^1^ and C^2^ *env* supports the notion that this motif may be associated with transient viral escape variants and mostly confer an evolutionary advantage only during a short period.

### Over-representation of C^2^ among V1 Cys insertions

We next assessed whether selective forces drive insertion of extra Cys on a population level. Using *env* sequences from the Los Alamos National Laboratory (LANL) HIV Sequence database (*n* = 6,657 sequences from unique PWH), we investigated the preference for one to four extra Cys insertions in V1. Among the 6,657 sequences, 79 V1 regions were C^1^ (1.2%) and 356 V1 regions were C^2^ (5.3%). C^3^ and C^4^ sequences were rare (<0.12 %) (Figure 6A). We observed a significant, albeit weak, correlation between the number of Cys residues and the length of the V1 hypervariable region (Kendall’s tau = 0.167, *p* = 1.2 × 10^−60^), suggesting that V1 length is only partially responsible for the insertion of Cys residues (Figure 6A). The position of the C^2^ Cys residues was skewed toward the N terminus of the V1 region, with a median distance to the canonical N-terminal V1 Cys of six amino acids (Figures S4E and S4F). Comparing the fractions of C^0^, C^1^, C^2^, C^3^, and C^4^ sequences between different subtypes confirmed that, across all subtypes except F, C^0^ sequences were the most common, followed by C^2^ (Figure 6B). The frequencies of C^1^/C^2^ *env* were comparable between most subtypes, except subtype G, which showed an astonishingly high frequency of C^2^ sequences (35/116 sequences, 30.2%) (Figure 6B). Out of the 116 analyzed subtype G sequences, C^2^ sequences were more likely to come from Nigeria, with 15/26 Nigerian subtype G sequences being C^2^, 10/26 C^0^, and 1/26 C^1^ (Fisher’s exact test, *p* value = 0.0032), suggesting that the high frequency of C^2^ Envs in subtype G may be due to limited sampling of geographically related sequences. Subtype G Envs from Nigeria were recently found to harbor higher levels of bnAb resistance coupled to longer and more glycosylated V1 regions as compared with subtype CRF02_AG Envs from Nigeria.^41^ Overall, these data illustrate that enrichment of the C^2^ motif is possible.

C^2^ sequences were strongly overrepresented compared with other categories of extra V1 Cys in all subtypes except F (Figure 6B). To analyze the probability of the observed C^2^ frequencies occurring by chance, we performed sequence simulations and generated distributions of C^1^, C^2^, C^3^, and C^4^ V1 sequences, assuming that Cys are randomly inserted with a probability calculated on their overall prevalence in the V1 of *env* (Figure S4G) (see STAR Methods). The likelihood of observing at least as many C^2^ sequences in the simulated dataset was <0.0001, strongly suggesting that the presence of C^2^ is non-random and selected for (Figure S4G). This observation held true when limiting the analysis to sequences of a single subtype; except for subtype F, all subtypes showed a very low probability of similar C^2^ frequencies occurring by chance (Figure 6B). The analyses of the LANL filtered web alignment suggest that there are selection pressures acting on the virus that select for the insertion of two Cys in the V1 hypervariable region.

### Comparable entry capacity and transmission potential of Env variants with extra V1 Cys

Occlusion of conserved Env regions by additional C^2^ bonds, including bnAb epitopes, may suggest that C^2^ variants have a selective advantage for nAb escape. In contrast, the low frequency and often transient nature of C^1^/C^2^ variants suggest that this motif may be associated with fitness deficits. When we examined the entry capacity of five Env clones from participant S5206G5 (three C^2^, one C^1^, and one C^0^ clone) and matched mutant Envs, we did not find a consistent, strong effect of V1 Cys content on pseudovirus infection of TZM-bl cells (<4-fold changes between C^0^, C^1^, and C^2^ Envs) (Figure S5). Thus, in the context of the S5206G5 bnAb-inducer Env, the extra V1 Cys motif is well tolerated, consistent with its existence in later Envs (Figure 3).

We hypothesized that the lack of fitness costs in chronic Envs from a bnAb donor (who would have experienced unique selection pressures) may not reflect overall fitness deficits that are consistent with the low population-level prevalence of this motif. We therefore used infection data from peripheral blood mononuclear cells (PBMCs) of 295 primary, replication-competent plasma virus isolates from the ZPHI cohort, sampled in acute and recent infection^42^ when autologous neutralization is low. Of the 295 isolates that likely represent transmitted viruses, 7.1% were C^2^, 1.3% C^1^, and 91.5% C^0^, and all groups had comparable replication capacity, with C^2^ recording a modest significant difference (*p* = 0.049) to C^0^ but not C^1^ (Figure 6C), confirming that viruses with extra Cys are fit and transmissible.

Putative fitness costs might be relevant only in the context of stronger immune selection pressure, such as that of bnAb prophylaxis. To address this, we used sequences from the antibody-mediated protection (AMP) trials HVTN703/HPTN081 and HVTN704/HPTN085,^43,44^ which showed the CD4bs bnAb VRC01 prevented acquisition of VRC01-sensitive HIV-1. In the two trials combined, 4,611 participants were enrolled in three arms: 10 mg/kg VRC01 (*N* = 1,537), 30 mg/kg VRC01 (*N* = 1,539), and placebo (*N* = 1,535). We assessed *env* clones from 172 participants who acquired HIV-1 by the 80-week endpoint.^43–45^ In total, 346 single-genome Env clones (one to six clones per participant) were derived from samples very close to infection (%28 days post last negative test). Nine participants (5.2%) had at least one C^1^ or C^2^ *env* sequence (Figure 6D). Notably, seven of these participants were in the placebo arm, two in the low-dose VRC01 arm, and none in the high-dose VRC01 arm, suggesting a reduced transmission capacity of C^1^ and C^2^ Envs in the presence of VRC01 (Fisher’s exact test, *p* value = 0.0289 for placebo vs. combined VRC01 arms). Collectively, these data confirm that C^1^ and C^2^ Envs are transmissible to a new host, where no neutralization pressure exists, but do not persist in the presence of strong selective pressure, accounting for their low overall prevalence (Figure 6E).

## DISCUSSION

Defining Env genetic features enriched in bnAb inducers is of interest for bnAb vaccine development as such features may contribute to the co-evolutionary process of bnAb elicitation. In previous research, we demonstrated that *env* genetics influence the specificity of bnAb activity,^21,22^ highlighting that translating this virus-encoded heritability of antibody responses to vaccine immunogens will be key to the success of HIV-1 vaccines.^26^

The identification of common HIV-1 Env signatures in bnAb inducers is challenging due to the immense genetic diversity of *env*. In particular, the V1V2 region shows high genetic diversity while simultaneously being an important regulator of overall neutralization sensitivity.^1,2,4^ Here, utilizing extensive data from the longitudinal SHCS and ZPHI cohorts, we show that the insertion of non-canonical Cys residues in V1 associates with elite bnAb induction. The insertion of Cys residues into V1 has previously been implicated as an escape strategy in some bnAb inducers.^6,31–33^ We extend this to show that additional V1 Cys are highly enriched in bnAb inducers, and we confirm that this is a common strategy used by the virus regardless of bnAb status, infection stage, and subtype.

We provide evidence for a multi-faceted role of extra V1 Cys in steering resistance to neutralization and in shaping bnAb activity (Figure 6E). HIV-1 selectively enriches for C^2^, as opposed to C^1^, on the population level, which we show to be linked with epitope shielding and modest increases in neutralization resistance. The over-representation of C^2^ compared with C^1^ suggests that the formation of an additional disulfide bridge contributes to resistance. It is plausible that this extra bond provides stabilization to the V1, which may be particularly beneficial after extensive V1 elongation, as seen in certain bnAb-inducer Envs. Furthermore, as we show by analysis of the LANL HIV-1 Sequence database, the occurrence of extra V1 Cys is associated with an extended V1 length. However, the C^2^ motif also exists in Envs with shorter V1 loops suggesting that structural fixation of the V1 is not restricted to long V1 loops.

Selection pressures drive within-host evolution of HIV-1.^46^ Although escape from single bnAbs can be achieved by mutating a few amino acids to inhibit bnAb-Env interactions, these mutations frequently come at a fitness cost for the virus.^47,48^ In contrast to single escape mutations, our data suggest that resistance conferred by V1 elongation and C^2^ insertions is not directed to a specific epitope region; rather, this appears to be a common strategy to increase resistance to multiple nAb epitope regions. The fact that the C^2^ Envs do not persist at high frequency in most donors suggests, however, that they are associated with other fitness deficits. We hypothesize that, in the absence of an optimal escape mutation, the modest level of resistance conferred by the C^2^ motif may allow sufficient residual replication to acquire mutations that improve resistance and fitness.

With increasing Cys content, the potential for aberrant disulfide bridging increases, which may affect the yields of correctly folded entry-competent trimers and, therefore, virus infectivity. Intriguingly, however, we did not observe strong entry capacity deficits linked to the C^2^ motif. Likewise, a replication capacity analysis of 295 transmitted founder (T/F) viruses from the ZPHI study^25,42^ indicated no deficit of C^2^ variants, highlighting that viruses with C^2^ motif can be transmitted. T/F Envs from a transmission pair carrying the C^2^ motif, which we previously documented (pair T6-R6 in Oberle et al.^49^), are consistent with this observation. Data from the CAPRISA cohort confirm that C^2^ Envs can be present very early in infection, underlining their transmission potential. Taken together, these results support the notion that the C^2^ motif is not itself a fitness constraint, but, given its overall low prevalence, the motif may associate with unfit escape variants that are rapidly counter-selected.

Our longitudinal cohort studies investigated the role of the C^2^ motif in escape in an autologous immune environment relevant for evolution and transmission. However, in the context of bnAb vaccines or bnAb prophylaxis, the transmitted virus would face a new type of antibody, likely different to that from which it escaped. Indeed, the analysis of breakthrough cases in the AMP trials revealed that C^1^/C^2^ Envs appear to have a deficit in transmission when facing potent bnAb activity. This finding is consistent with the notion that additional V1 Cys confer a sufficient advantage to the virus only in a particular environment and at a particular time to become predominant. If these variants experience an altered immune environment in which their associated resistance patterns are no longer advantageous, e.g., due to newly emerging autologous nAbs or through bnAbs in prevention, as in the case of the AMP trial, fitness deficits will prevail, and they will be counter-selected (Figure 6E). However, we also present several examples of donors retaining additional V1 Cys longitudinally, including from early time points after infection.

Viruses from bnAb inducers that have co-evolved with an ever broader and more potent neutralization response are likely optimized for both fitness and resistance, increasing the likelihood that the added resistance provided by the C^2^ motif will remain important, be conserved, and become predominant. In support of this, two bnAb inducers we previously defined as a transmission pair,^26^ S30402 and S30349 (Figure 1A; Table S2), both had viruses with V1 Cys insertions present already in the early V3-glycan escape variant Env and both subsequently mounted a CD4bs bnAb response.^26^

We hypothesize that the C^2^ motif may contribute to the bnAb imprinting capacity of Env by improving stabilization of trimer regions, as has been previously shown in soluble trimers with synthetically disulfide-stabilized V1V2 regions.^50^ We provide evidence for this scenario by studying the Env of the V2-apex bnAb-inducer S5206G5, which evolved to create a C^2^ variant, reducing access to several bnAb epitopes, as expected, but increasing access to the V2-apex region. This suggests that escape from early autologous neutralization activity necessitated a V1 elongation that was stabilized by the C^2^ motif. This introduction of C^2^ occurred at a stage when V2-bnAb activity had not yet evolved, suggesting that the antigenic features of the C^2^ variant Env contributed to the evolution of V2-apex bnAb activity in this donor. The role of autologous responses leading to V1 elongation and Cys insertion is supported by the fact that C^2^ *env* was also detected in donors without cross-neutralizing activity.

Our data provide insights to the link between bnAb induction and insertion of V1 Cys. We describe two possible roles for the V1 Cys insertions in the context of bnAb development: they may aid autologous nAb/bnAb escape by shielding epitope regions and they may promote bnAb development by presenting a more stabilized immunogen. Both scenarios have implications for vaccine development. BnAb development in natural infection typically takes years, and multiple rounds of co-evolution between B cell receptor repertoires and Env are necessary. The association between the presence of Cys insertions in the V1 loop and bnAb induction may highlight an important intermediate step in the co-evolution, where pre-fusion Env is sufficiently stabilized for further maturation of bnAb lineages toward breadth. Furthermore, by harnessing the differential bnAb epitope exposure of C^0^/C^2^ Envs, C^0^/C^2^ variants of candidate trimers can generate immunogens with structural diversity. This, in turn, could be exploited in the design of boost immunogens to advance initial binding antibody responses toward neutralization breadth.

### RESOURCE AVAILABILITY

#### Lead contact

Requests for further information and resources should be directed and will be fulfilled by the lead contact, Alexandra Trkola (trkola.alexandra@virology.uzh.ch).

#### Materials availability

Plasmids encoding the soluble gp140 trimers and *env* used in this study are available through a standard MTA from the lead contact upon request.

#### Data and code availability

- Source data for analyses in Figures 5A–5E are available in Table S9.
- The *env* sequences of the bnAb-inducer Env pseudovirus panel are accessible in GenBank (accession numbers GenBank: PQ282666-PQ282697, MH714324, and MH714327).
- This paper does not report original code.
- Any additional information required to reanalyze the data reported in this work paper is available from the lead contact upon request

## STAR★METHODS

### EXPERIMENTAL MODEL AND STUDY PARTICIPANT DETAILS

#### Clinical specimens

The study utilized biobanked plasma samples of PWH identified as bnAb-inducers in the Swiss 4.5K screen^21,22^ for HIV-1 Env cloning and neutralization analyses. The specimen were obtained from the biobanks of the Swiss HIV Cohort study (SHCS)^23,24^ and the Zurich Primary HIV Infection Study (ZPHI).^25^ The SHCS is registered under the Swiss National Science longitudinal platform (http://www.snf.ch/en/funding/programmes/longitudinal-studies/Pages/default.aspx#Currently%20supported%20longitudinal%20studies). The SHCS data are collected by the five Swiss University Hospitals, two Cantonal Hospitals, 15 affiliated hospitals and 36 private physicians (listed in http://www.shcs.ch/180-health-care-providers).The ZPHI is an ongoing, observational, non-randomized, single center cohort founded in 2002 that specifically enrolls patients with documented acute or recent primary HIV-1 infection (ClinicalTrials.gov, identifier NCT00537966). The SHCS and the ZPHI were approved by the ethics committees of the participating institutions (Kantonale Ethikkommission Bern, Ethikkommission des Kantons St. Gallen, Comité Departemental d’Éthique des Spécialités Médicales et de Médicine Communautaire et de Premier Recours, Kantonale Ethikkommission Zürich, Repubblica et Cantone Ticino–Comitato Ethico Cantonale, Commission Cantonale d’Éthique de la Recherche sur l’Être Humain, Ethikkommission beider Basel for the SHCS and Kantonale Ethikkommission Zürich for the ZPHI), and written informed consent was obtained from all participants.

The study utilized biobanked plasma specimen of PWH with known bnAb-inducer or non-neutralizer status enrolled in the CAPRISA cohorts for V1 generated using the PacBio-SMRT UMI sequencing protocol.^39^ The CAPRISA 002 Acute Infection cohort was established in 2004, and has been following women from early/acute stage of infection. Env sequences were truncated to the V1 hypervariable region to assess the number of Cys residues. The CAPRISA 002 study protocol was reviewed and approved by the Biomedical Research Ethics Committees of the University of KwaZulu-Natal (E013/04), the University of Cape Town (025/2004) and the University of the Witwatersrand (MM040202).

Demographic characteristics of the analyzed cohort participant subsets are indicated in Table S11. Analyzed participants from the SHCS/ZPHI cohorts were predominantly of male sex (83%) and white ethnicity (86%). Analyzed participants from the CAPRISA 002 cohort were exclusively black women (100%).

Analyses of the influence of host parameters on the presence of non-canonical V1 Cys residues in Env were adjusted for ethnicity and a variable combining sex and transmission mode (Figure 5B).

#### Cell lines

HEK 293T cells (American Type Culture Collection, USA) and TZM-bl cells (NIH AIDS Reagent Program, USA) were cultivated in DMEM supplemented with 10% heat-inactivated FBS, 100 U ml^−1^ penicillin and 100 μg ml^−1^ streptomycin (all from Gibco, Thermo Fisher Scientific, USA) at 37 °C, 5% CO2 and 80% relative humidity. Expi293F suspension cells (Thermo Fisher Scientific, USA) for protein expression were maintained in serum-free Expi293F expression medium (Thermo Fisher Scientific, USA), according to the manufacturer’s instructions. Cells were regularly tested for mycoplasma contamination and tested negative. No cell line authentication was performed.

### METHOD DETAILS

#### HIV-1 full-length Env cloning

HIV-1 full-length Env cloning from bnAb inducers was performed using undiluted cDNA generated from plasma virus RNA as described in Kouyos et al.^26^ Briefly, viral RNA was extracted using the NucliSENS easyMAG platform according to the manufacturer’s instructions. RNA samples were used in an Env RT-PCR reaction using the PrimeScript One Step RT-PCR Kit (Takara Bio, Japan) and primers MSR5 and E27. The RT-PCR product was then used as a template in a second PCR with the high-fidelity KAPA HiFi PCR kit (Kapa Biosytems) and primers MascA InFusion and MascM InFusion to amplify the *env* gene and add 14–15 bp 5’-extensions. The final amplicons were cloned into pcDNA3.1/V5-His vector (Invitrogen, Thermo Fisher Scientific, USA) linearized with EcoRV/BamHI using the InFusion protocol (Takara Bio, Japan) according to the manufacturer’s instructions, and heat-shock transformed into XL10 gold ultra-competent *E. coli* (Agilent Technologies, USA). After plating the transformed bacteria, 12–24 random clones were picked from each cloning reaction, and *env* plasmid DNA was recovered from small scale bacterial cultures using the Qiagen Plasmid Plus 96 well miniprep kit (Qiagen) kit according to the manufacturer’s instructions.

#### Env clone entry capacity screening

To assemble the bnAb inducer Env pseudovirus panel, and the longitudinal S5206-G5 Envs, entry capacity of the cloned Envs was measured. Env-encoding plasmids were co-transfected with the luciferase reporter HIV vector pNLluc-AM into HEK 293T cells as described^55^ in 96-well plates. Entry competence of the produced small-scale Env pseudoviruses was assessed on TZM-bl cells and luciferase activity (relative light units (RLU)) was measured three days post-infection using Luciferase assay reagents (Promega, USA). Only entry-competent Envs showing RLU values >10-fold over background were studied further. If several entry-competent Envs were retrieved, one was selected at random for inclusion in the bnAb inducer pseudovirus panel (N=34; Table S2). For the longitudinal Env characterization of participant S5206-G5, all entry competent Envs were analyzed.

#### Sequencing of clonal *env* variants

Sequencing libraries of the selected Env clone plasmids included in the bnAb inducer Env pseudovirus panel and in the participant S5206-G5 longitudinal sequencing were prepared with NexteraXT (Illumina, USA), sequenced using the MiSeq Reagent Kit v2 (50 cycles) (Illumina, USA) and Env sequences retrieved by EnvSeq (https://github.com/medvir/EnvSeq). Additionally, V1 sequences were derived from Env cloned from other SHCS participants (N=750 V1 sequences from 77 unique participants) to study extra Cys content in V1 (Table S9).

#### Nanopore sequencing and haplotyping of S5206G5 *env* amplicons

In addition to clonal *env* sequences derived from 12 plasma time points of participant S5206G5, PCR-amplified *env* from 8/12 time points was sequenced using Oxford Nanopore technology and haplotyping was performed with ShoRAH.^54^ The PCR product obtained from the above described *env* PCR was sequenced on MinION and GridION flow cells (R10.4.1) using the ligation sequencing kit SQK-LSK114 (Oxford Nanopore Technologies, UK). The obtained sequencing reads were filtered by quality (Phred ≥ 20) and length (1–1.5 times the length of the consensus sequence). A random subset of 1,000 filtered reads per time point was then analyzed by ShoRAH using the amplicon method (https://github.com/cbg-ethz/shorah).^54^ A BAM file generated with a reference sequence for each time point, obtained from the consensus sequence of the clonal *env* sequences for each time point and the subset of 1,000 reads per time point, was used as input. Haplotypes generated from ShoRAH were then filtered for > 0.95 posterior probability, aligned to the HXB2 reference *env* sequence and analyzed for V1 Cys content.

#### Env mutagenesis

All mutagenesis reactions of Env-expressing plasmids for pseudovirus production and for soluble Env production were performed using the QuickChange II XL kit (Agilent Technologies, USA) according to the manufacturer’s instructions using 10 ng of plasmid template encoding the respective Env, 125 ng of forward and 125 ng of reverse mutagenesis primers per reaction. Successful mutagenesis was confirmed with Sanger Sequencing (Microsynth, Switzerland).

#### HIV-1 Env pseudoviruses

Env-pseudotyped viruses were prepared by co-transfection of HEK 293T cells with plasmids encoding the respective *Env* genes and the luciferase reporter HIV vector pNLluc-AM^51^ as described.^55^ Input of Env pseudoviruses for neutralization assays was adjusted to yield virus infectivity corresponding to 5,000–20,000 relative light units (RLU; measured on a Dynex MLX reader, Dynex Technologies, USA) obtained by infection of TZM-bl cells in 96-well microtiter plates in the absence of inhibitors. Details on Env-pseudotyped viruses generated with corresponding GenBank entry and subtype is provided in Tables S2 and S4.

The 34 Envs included in the bnAb-inducer panel (Table S2) were isolated on average 438 weeks (8.4 years) post the estimated date of infection (EDI), the average calendar year of isolation was 2005. The 41-virus panel comprised Envs from both acute and chronic stage viruses including Transmitted /Founder (T/F) Envs and Envs form the global neutralization reference panel.^56–58^ The average calendar year of isolation was 2005. For 35 Envs included in the 41-virus panel estimates on the infection stage at isolation were available: acute or acute T/F (N=7), <3 months (N=8), <12 months (N=12), chronic (N=8).

The information on the 123 CATNAP-chronic Envs was derived from the CATNAP database^27^ where they were specified to have been isolated in chronic infection without further details on years post EDI. The average year of isolation of the CATNAP chronic Env panel was 2000 (Table S5). Overall, per reference bnAb, IC50 values for 14–114 of the total 123 analyzed CATNAP-chronic Envs were available (see Table S5 for the full listing).

#### Neutralization assays

The neutralization activity of plasma and mAbs was tested on TZM-bl cells using Env-pseudotyped viruses in a 384-well format using a PerkinElmer EnVision Multilabel Reader (PerkinElmer, Inc., USA) to record luciferase reporter activity as described.^55^ 4-fold serial dilutions of plasma and Abs were tested, with starting concentrations of 1:100 and 25 μg/ml, respectively. Concentrations correspond to the final assay volume. The low antibody dose recorded in Figure S2D corresponds to dilution 6 of the 3-fold serial dilution of Abs. The plasma or antibody concentrations causing 50% reduction in viral infectivity (half-maximum inhibitory concentration, IC50) were calculated by fitting data to sigmoid dose–response curves (variable slope) using Prism (GraphPad Software, Inc., USA). If 50% inhibition was not achieved at the highest or lowest inhibitor concentration, a greater than or less than value was recorded.

#### bnAb plasma neutralization specificity

Neutralization specificity of bnAb inducer plasma was defined through neutralization fingerprints of the plasma analyzed in the Swiss 4.5K screen using the fingerprinting method described there.^21^ Briefly, plasma neutralization activity against a 41-virus multi-clade panel (Table S4) was compared to neutralization activity of 43 reference bnAbs (Table S3). For each plasma, the dominant plasma bnAb specificity was predicted using the maximal-Spearman-based prediction (MSBP) as described in Rusert et al.^21^ Plasma neutralization specificity based on the fingerprint results are listed in Tables S1 and S6.

#### V1 sequence analysis from SHCS and ZPHI participants

The V1 sequence analysis of SHCS and ZPHI participants (Figures 5A–5C, S4B, and S4C) included consensus V1 sequences from published data^34,35^ and V1 sequences from individual full-length Env cloned from Swiss 4.5K and SHCS/ZPHI participants (N=750 sequences from 77 participants) in this study (Table S9). Consensus V1 sequences (N=6424) were derived from near full-length HIV-1 genome next generation sequence (NGS) data sets of plasma virus from the SHCS and ZPHI cohorts that were generated by long overlapping amplicon sequencing on Illumina MiSeq^34,35^ followed by assembly with an in-house sequence alignment tool (available at https://github.com/medvir/SmaltAlign). Alignment was done with an initial *de novo* assembly assisted alignment to the HIV-1 reference genome HXB2, followed by three alignments against the iteratively improved consensus sequence.^59^ From the final sequence alignment, the majority consensus sequence was generated with a depth threshold of ≥20 for each position. The respective nucleotide regions were extracted from each sequence using the local version of NCBI BLAST.^60^ The BLAST database consisted of the appropriate regions from a panel of 459 reference sequences obtained from the Los Alamos HIV sequence database (https://www.hiv.lanl.gov/). To obtain the final consensus sequences on amino acid level, we made codon alignments of all blasted gene sequences using MACSE v2, to account for frameshifts, for the amino acid translation.^61^

For all analyses, we excluded sequences from participants without EDI estimate available, and sequences lacking the conserved Cys residues that flank the V1 hypervariable region (HXB2 positions 131 and 157) to exclude non-functional Env sequences. Criteria for sequences inclusion/exclusion in the respective analyses are depicted in Figure S4A. Consensus and clone-derived V1 sequences were compiled alongside parameters on infection length, time, bnAb status and demographic characteristics as available from the Swiss 4.5K screen^21^ or the SHCS and ZPHI databases for the analyses in Figures 5A–5C, S4B, and S4C (see Table S9 for full source data).

To analyze interdependencies between bnAb status and occurrence of extra V1 Cys, we used V1 sequence data from Swiss 4.5K participants. To ascertain a relationship between V1 features and neutralization status, the analysis was restricted to sequences sampled within one year of the plasma neutralization measurement in the Swiss 4.5K screen^21,22^ that defined the bnAb status and that were collected a minimum one year post the EDI (Figure S4A). The selection criteria were fulfilled for 507 of the 4,484 Swiss 4.5K participants.

To probe the prevalence of extra V1 Cys at different disease stages by monitoring the frequency of V1 Cys insertions as a function of duration of untreated HIV-1 infection plasma neutralization data was not required, allowing a total of V1 sequences of additional SHCS and ZPHI donors to be included (total N = 1481 sequences, one per donor; Figure S4A).

#### V1 sequence analysis of CAPRISA 002 participants

The V1 sequence analysis of CAPRISA 002^37^ participants with known bnAb-inducer status^62–64^ (Table S9; Figures 5D and 5E) included V1 sequences derived by PacBio UMI Env sequencing using a method modified from^39^ to generate products with single PCR, instead of nested PCR. See Table S9 for source data.

#### V1 sequence analysis of AMP trial breakthrough viruses

The V1 sequence analysis of AMP trial breakthrough viruses^43,45^ (Figure 6D) included single-genome V1 sequences derived by PacBio UMI Env sequencing Env sequencing from^45^ made publicly available at https://atlas.scharp.org/cpas/project/HVTN%20Public%20Data/HVTN%20704%20HPTN%20085%20and%20HVTN%20703%20HPTN%20081%20AMP/begin.view.

#### V1 sequence analysis of LANL database

HIV sequences from the 2021 Los Alamos HIV Sequence Database M group with CRFs Env amino acid filtered web alignment (alignment ID: 121P2) were analyzed, which consists of 6657 sequences, with only one sequence per PWH and with very similar sequences having been removed. Additional quality control steps were performed when generating this alignment, including removal of the following sequences: those defined as ‘problematic’ by the Los Alamos HIV Sequence Database, those with >1 frameshifts, and those with significant insertions and deletions (as assessed by manual curation).

To determine whether sampling biases were contributing to the high frequency of C^2^ sequences in subtype G, infection country was obtained from the Los Alamos HIV Sequence Database. For countries with at least 5 C^0^ and/or C^2^ sequences (Cameroon, Nigeria, and Spain), we performed Fisher’s exact tests on subtype G sequences comparing number of C^0^ and C^2^ sequences in each of these countries versus all other sequences. Statistics were Bonferroni corrected.

To assess the probability of the observed frequency of C^2^ sequences, sequence simulations were performed. For each sequence in the filtered web alignment, an equally long V1 hypervariable region sequence was simulated, randomly assigning Cys at each position, based on the overall frequency of Cys in all V1 sequences (P(C) = 0.00565). Simulations were repeated 10,000 times to generate a theoretical distribution of sequences with extra Cys. P-values were calculated by comparing the frequency of C^2^ sequences in the simulated dataset with the observed frequencies.

#### Soluble native-like Env trimer variants expression and purification

Codon-optimized sequences of strain S5206G5–42wk-c3 gp140 wild type comprising amino acids 31–664 (HXB2 numbering) and containing stabilizing mutations including the SOSIP or the DS-SOSIP mutations (Table S8) were custom synthesized (Thermo Fisher Scientific, USA) and cloned into the CMV/R expression vector inframe with a C-terminal AviTag.^52^ Env proteins were produced in Expi293F suspension cells (Thermo Fisher Scientific, USA) by transient transfection using a furin-expressing helper plasmid^53^ at a 3:1 ratio (w/w). HIV-1 Env proteins were purified from culture supernatants using *Galanthus nivalis* lectin agarose-resin (Vector Laboratories, USA) and C-terminally biotinylated using the BirA enzyme (Avidity Biosciences, USA) according to the manufacturer’s instructions. Subsequent size exclusion chromatography (SEC) (Cytiva, USA) on a Superdex 200 increase 10/300 GL was performed to derive the trimer protein fraction.

#### Binding antibody multiplex assay

Binding of mAbs to soluble trimers was measured with a bead-based binding antibody multiplex assay (BAMA). MagPlex^®^-Avidin microspheres (Luminex) were washed before use in 100 μl phosphate buffered saline (PBS) with 2% FCS using a Magnetic Plate Separator (Luminex). Biotinylated AviTag HIV-1 envelope trimers (100 nM) were coupled to washed 5×10^5^ MagPlex^®^-Avidin microspheres in a total volume of 150 μl PBS/2% FCS. Coupled beads were stored at 4°C for up to 4 weeks before use in the assay. On the day of analysis, per bnAb tested, 1,000 coupled beads were washed twice with 100 μl 2% FCS in PBS and resuspended in 100 μl 2% FCS in PBS. The different trimer-coupled microspheres were pooled and incubated with 5 μg/ml of bnAbs (IgG1) in a total volume of 100 μl at room temperature for 1h under continuous shaking at 750 rpm on a Heidolph Titramax shaker. After washing microspheres twice with 2% FCS in PBS, PE-labeled detector antibody (mouse anti-human IgG-Fc; Southern Biotech, Cat#9040–09, clone JDC-10; 1 μg/ml) was added and beads were incubated at room temperature for 1h on a shaker, followed by two wash steps with 2% FCS in PBS. Bound PE-detector Ab was recorded as mean fluorescence intensity (MFI) on a FlexMap 3D instrument (Luminex). A minimum of 50 beads per antigen and antibody were acquired to guarantee accurate MFI values.

#### Entry capacity of S5206G5 C-variants

The entry capacity of Env variants was assessed by measuring the infectivity of Env pseudoviruses on TZM-bl cells, normalized to the p24 content of pseudovirus stocks. Infectivity and p24 content were measured in parallel from the same transfection and measurements were performed in biological duplicates. For the infectivity measurements, pseudovirus serial dilutions were added to TZM-bl cells and luciferase activity (relative light units (RLU)) was measured three days post-infection using Luciferase assay reagents (Promega, USA). In parallel, pseudovirus serial dilutions were inactivated with 2% Empigen in PBS and p24 content of virus lysates was measure by ELISA as previously described.^65^ Briefly, p24 was captured on anti-p24 antibody (D7320, Aalto Bioreagents, Ireland) coated plates and detected using alkaline-phosphatase-coupled antibody BC1071-AP (Aalto Bioreagents, Ireland). P24 content was then interpolated from a standard curve of recombinant p24 (AG 6054, Aalto Bioreagents, Ireland). The obtained infectivity (RLU/μL) values were normalized to the p24 concentrations (ng/μL) of all virus stocks.

#### Replication capacity

Replication capacity of primary virus isolates from the ZPHI studied analyzed in Figure 6C were derived from Rindler et al.^42^

#### Figure preparation

Figures were prepared either using R version 4.3.1 and the ggplot2^66^ package or in GraphPad Prism version 10.1.0 (GraphPad Software, USA). Figures were assembled and finalized in Affinity Designer (Serif Europe Ltd, United Kingdom).

### QUANTIFICATION AND STATISTICAL ANALYSIS

Statistical analyses were done in R (version 4.3.1) and in GraphPad Prism (Version 10.1.0, GraphPad Software, Inc., USA). Linear mixed-effects models and linear regressions were fitted using the lme4 package in R.^67^ Significance thresholds were Bonferroni corrected for multiple testing where applicable. Statistical details for each experiment can be found in the corresponding figure legends.

## Supplementary Material

MMC2

MMC3

MMC1

MMC4

SUPPLEMENTAL INFORMATION

Supplemental information can be found online at https://doi.org/10.1016/j.chom.2025.01.004.

## Figures and Tables

**Figure 1.**
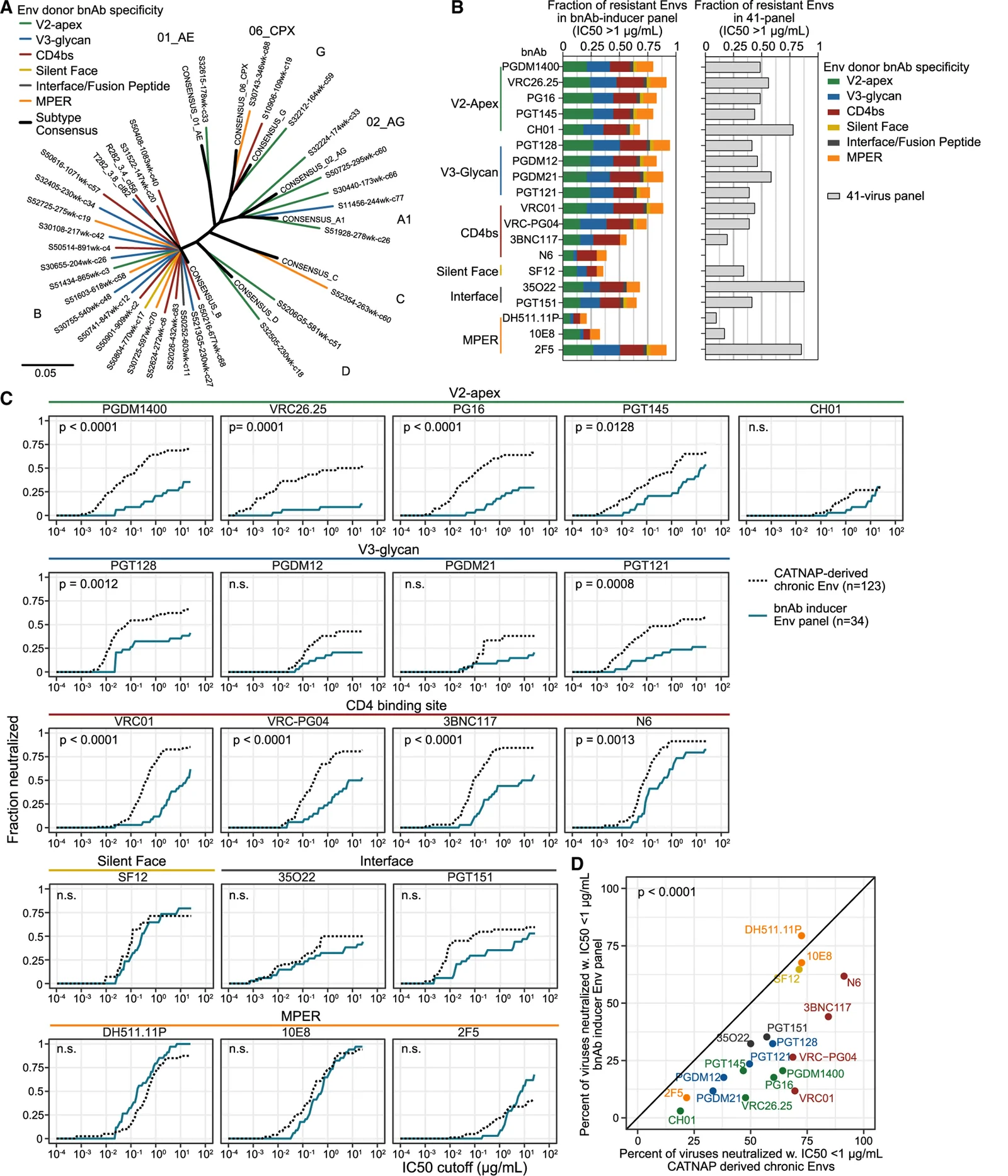
bnAb-inducer Envs have a generalized neutralization resistance (A) Phylogenetic tree of *env* nucleotide sequences of bnAb-inducer Env panel (*n* = 34 Envs) (Table S2). Branch color indicates the predicted plasma bnAb specificity of the donor. Env subtype consensus sequences (2005 version) from the LANL HIV Sequence database are shown as thicker black lines. (B) Resistance of the bnAb-inducer Env panel (*n* = 34) to known bnAbs (left) and multiclade 41-panel (right). Resistance to each bnAb is quantified as the fraction of Envs with a half-maximal inhibitory concentration (IC50) > 1 μg/mL. Colors indicate the predicted plasma bnAb specificity of the donor. (C) Cumulative frequency distribution (breadth-potency curves) of the IC50 values of 19 bnAbs against the bnAb-inducer Env panel (blue) and CATNAP-derived chronic Envs (black dotted line) (123 Envs sampled in chronic infection). *p* values are derived from Wilcoxon test by comparing median IC50 of each bnAb against the bnAb-inducer and CATNAP-Envs (Figures S1D and S1E). (D) Comparison of the percentage of highly sensitive Envs (IC50 < 1 μg/mL) in the CATNAP-chronic Env (x axis) and bnAb-inducer Env panels (y axis) for each of the 19 bnAbs tested. *p* value calculated with t test comparing mean breadth of all antibodies between the two panels.

**Figure 2.**
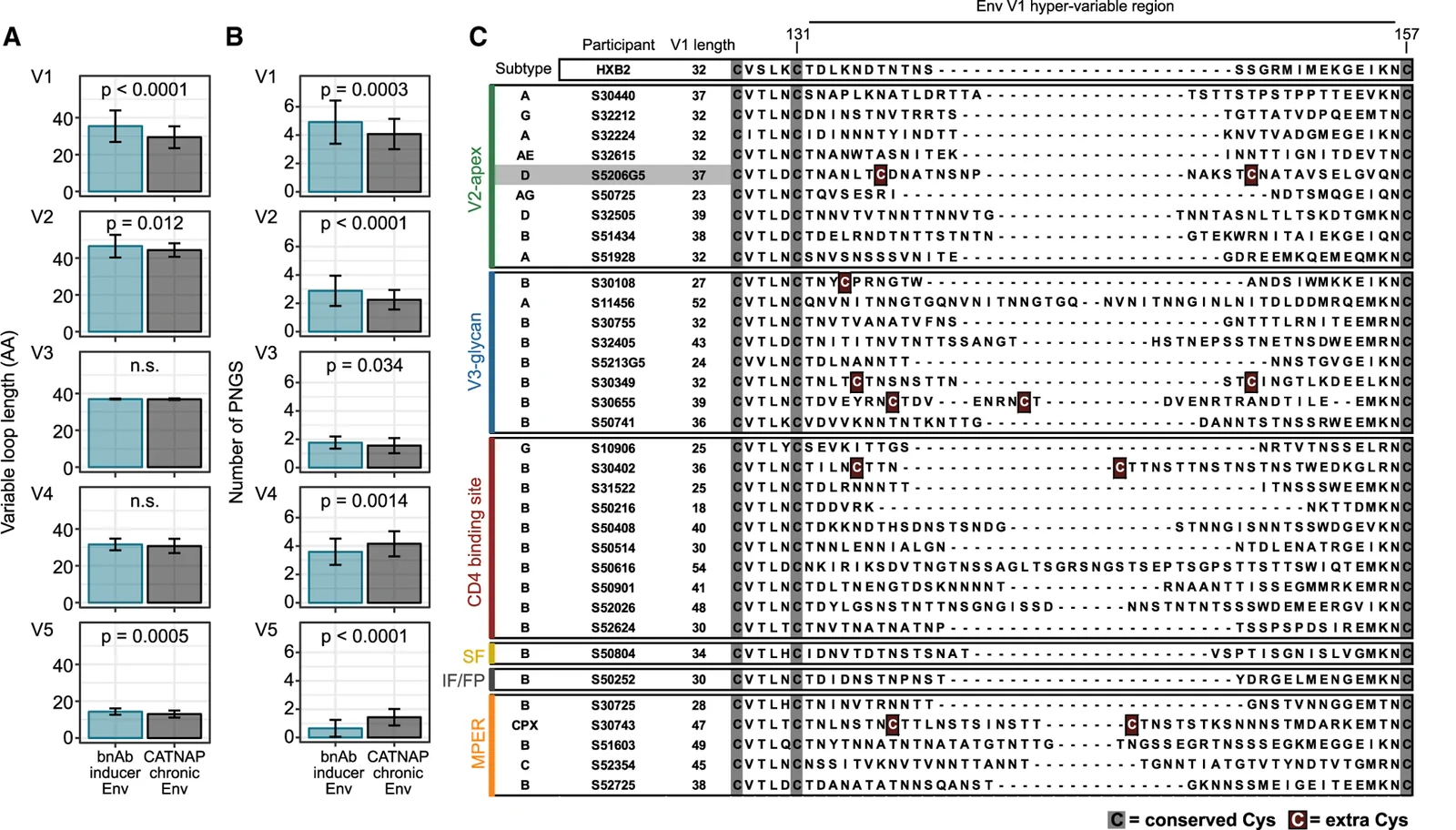
bnAb-inducer Envs have longer V1 loops with non-canonical cysteine residues (A) Comparison of variable loop length (V1-V5) of bnAb-inducer Envs (*n* = 34) CATNAP-derived chronic Envs (*n* = 123). Bars show mean loop length, and error bars show standard deviation. *p* values calculated with t test on the mean length in each group. (B) Comparison of the number of predicted N-linked glycosylation sites (PNGS) in V1–V5 between bnAb-inducer Envs (*n* = 34) CATNAP-derived chronic Envs (*n* = 123). Bars show mean number of predicted N-linked glycosylation sites (PNGS), and bars show standard deviation. *p* values calculated with t test on the mean number of PNGS in each group. (C) Multiple sequence alignment of the V1 hyper-variable region (HXB2 residues 126–157) of the bnAb-inducer Env panel. Gray and burgundy boxes indicate conserved and extra V1 Cys, respectively. The predicted specificity of the bnAb-inducer plasmas from which the Envs were isolated is indicated on the left.

**Figure 3.**
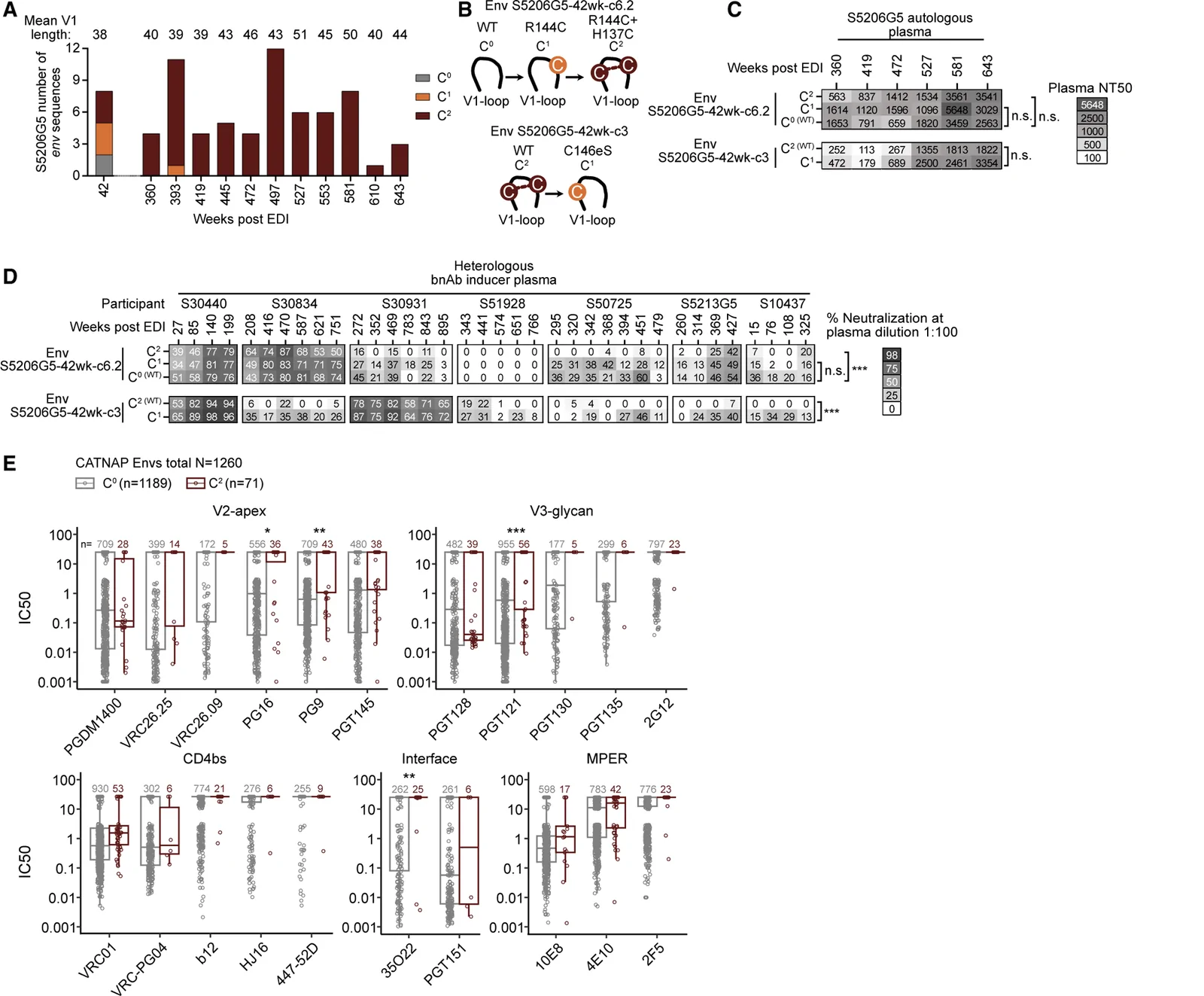
Effects of V1 Cys insertion in bnAb-inducer S5206G5 Env on plasma neutralizing activity (A) Number of S5206G5 Env variants with extra V1 Cys per sample time point (42–643 weeks post EDI). Mean V1 length of each time point indicated above. (B) Mutations introduced in the wild-type (WT) C^0^ clone S5206G5–42wk-c6.2 and WT C^2^ clone S5206G5–42wk-c3 to generate variants with altered V1 Cys content. (C) Longitudinal autologous plasma neutralization of WT and V1 Cys variant Envs. Heatmap depicts the reciprocal half-maximal neutralization plasma dilution (NT50). Significance of neutralization differences was assessed by linear regression (ns, not significant). (D) Neutralization activity of heterologous bnAb-inducer plasma (seven donors, longitudinal time points) against S5206G5 V1 Cys variants shown in (C). Heatmap depicts the percent neutralization at 1/100 plasma dilution. Differences in neutralization sensitivities were assessed with linear mixed effects models to account for multiple plasma from the same donor in the analysis (ns, not significant, ***p value < 0.001). (E) Comparison of neutralization IC50s of bnAbs between C^0^ and C^2^ Envs derived from CATNAP. Boxplots show median IC50s and quartiles. Bonferroni-adjusted *p* values are derived from a mixed effects model with V1 Cys content and virus subtype as fixed effects, assessing the influence of C^2^ on the IC50s of each bnAb (*p value < 0.05, **p value < 0.01, ***p value < 0.001).

**Figure 4.**
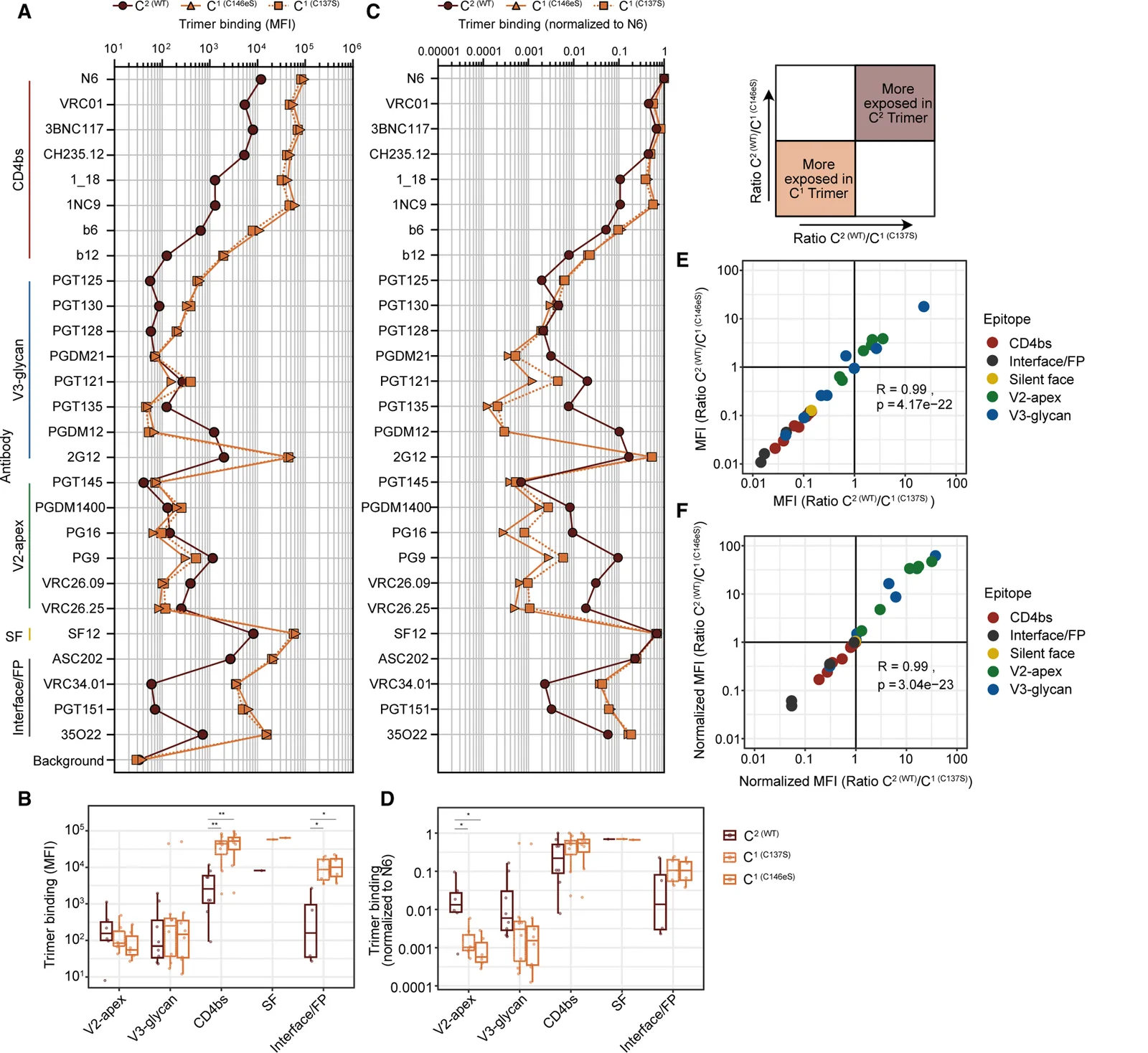
Extra V1 Cys modulate bnAb epitope exposure (A) Luminex bead-based binding antibody multiplex assay (BAMA) of bnAbs (*n* = 27) against soluble DS-SOSIP trimers of S5206G5–42wk-c3 Env variants: WT trimer (C^2^), with a C137S mutation (C^1^) and with a C146eS mutation (C^1^). Data depict mean fluorescence intensity (MFI). (B) Boxplots depicting the median MFI values from (A) with antibodies grouped by epitope. Differences in epitope exposure were assessed with Wilcoxon tests (*p value < 0.05, **p value < 0.01). (C) MFI values shown in (A) normalized to bnAb N6, which achieved the highest MFI signal in all datasets. (D) Boxplots depicting N6-normalized MFI values with antibodies grouped by epitope. (E and F) Proportional differences in binding of C^2^ compared with C^1^ trimer versions based on data shown in (A) and (B), respectively. Each dot represents one bnAb, colors denote the bnAb epitope. Pearson correlation results of fold-change values are shown.

**Figure 5.**
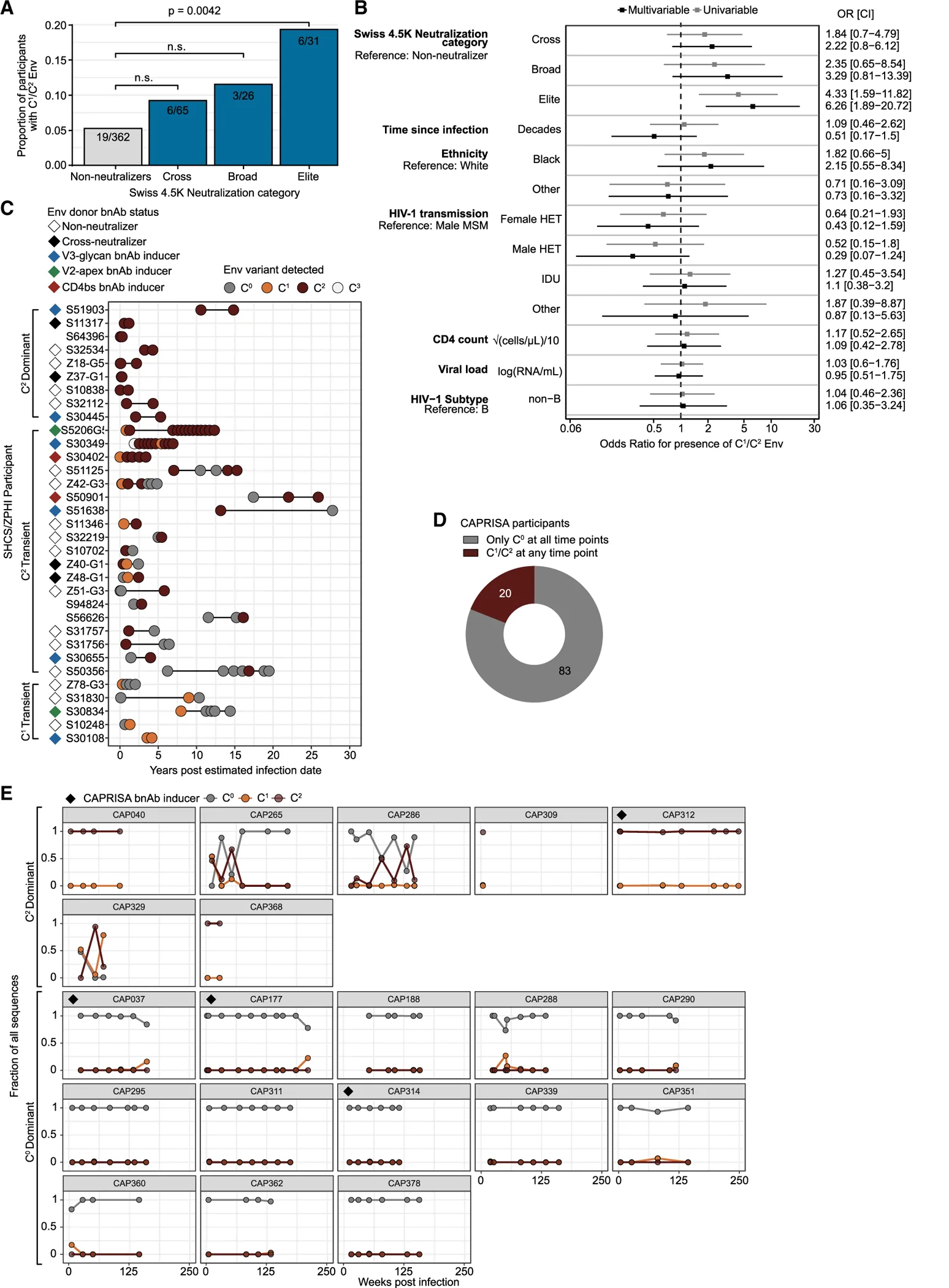
Non-canonical V1 Cys are enriched in bnAb inducers (A) Frequencies of donors with C^1^/C^2^ Env among 484 Swiss 4.5K participants with known neutralization status.^21^ *p* values calculated with logistic regression. (B) Uni- and multivariable logistic regression models assessing the independent contribution of host factors on the presence of C^1^/C^2^ Env in Swiss 4.5K donors analyzed in (A). (C) Longitudinal V1 sequence analysis of 33 SHCS and ZPHI participants accounting for presence of C^0^, C^1^, C^2^, and C^3^ *env* in NGS consensus sequences or cloned *envs*. Each dot represents a time point. The dot color indicates the number of extra V1 Cys observed in the sequence. Diamonds on the left indicate the bnAb-inducer status and plasma specificity of the Env donors. (D) Longitudinal V1 sequence analysis of CAPRISA donors (*N* = 103) indicating the proportion of donors with (C^1^ or C^2^) or without extra V1 Cys detected at any time point based on single-genome deep-sequencing data. (E) Longitudinal frequencies of Envs with different V1 Cys content for 20 CAPRISA participants with at least one C^1^ or C^2^ sequence. The proportion of observed C^0^/C^1^/C^2^ variants is depicted.

**Figure 6.**
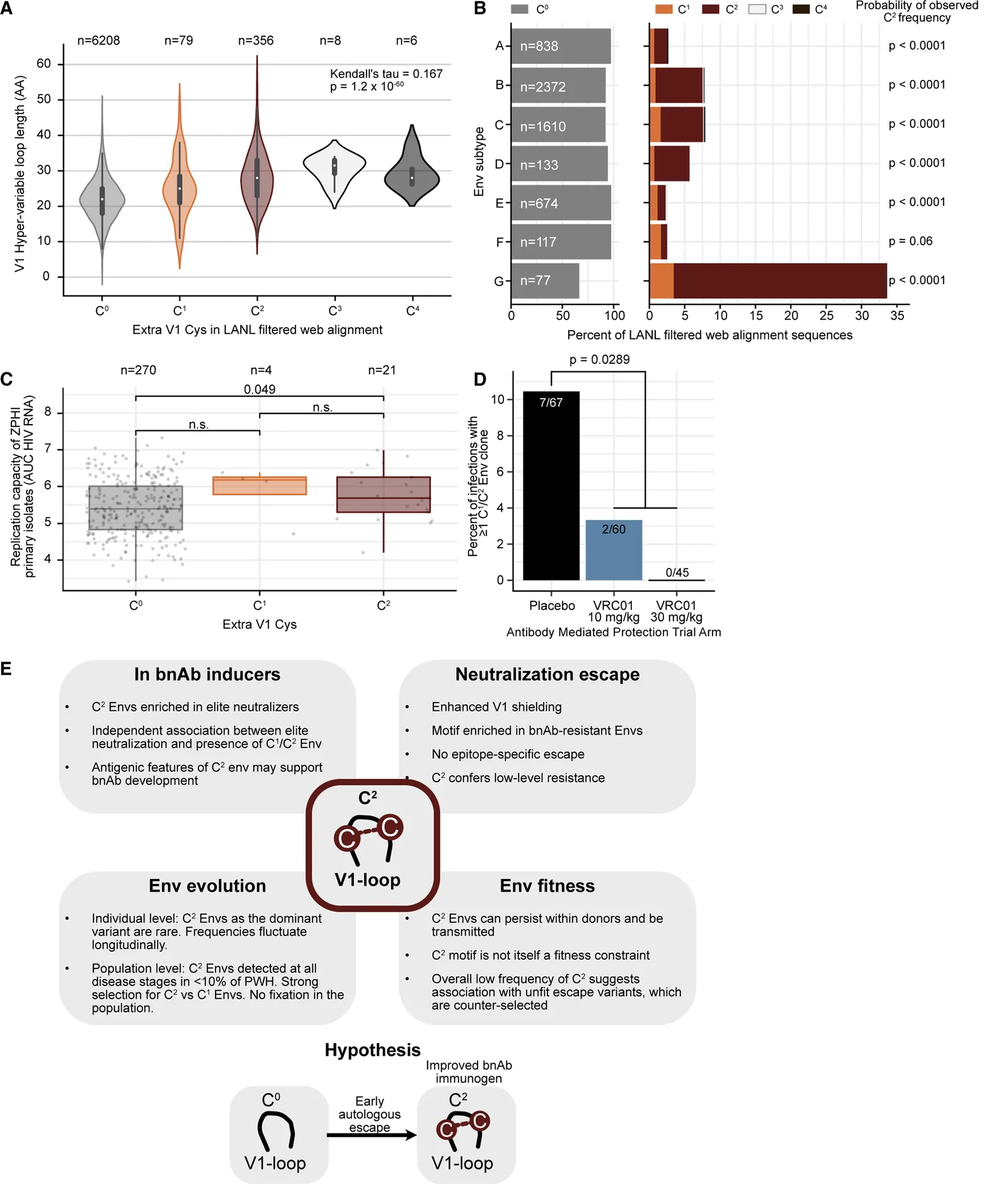
Fitness and selection of V1 with non-canonical Cys (A) Correlation analysis of the length of the V1 hypervariable region and the number of Cys in V1 using the LANL HIV-sequence-database-filtered web alignment (*n* = 6,657 *env* sequences). (B) Frequencies of C^0^, C^1^, C^2^, C^3^, and C^4^ Env sequences in different HIV-1 subtypes in the LANL HIV-sequence-database-filtered web alignment. V1 hyper-variable loop sequences were simulated for each subtype, 10,000 times. *p* values show the probability of observing at least as many C^2^ sequences in the simulated dataset as in the filtered web alignment. (C) Comparison of the replication capacity data of C^0^, C^1^, and C^2^ primary HIV-1 isolates (*n* = 257) sampled in acute to recent infection. Infectivity and sequence data are from the ZPHI cohort published in^42^ . Boxplots show median replication capacity and quartiles, *p* values were calculated using a Student’s t test. (D) Analysis of C^1^/C^2^ occurrence among 172 participants who acquired HIV-1 by the primary endpoint of 80 weeks in the HVTN703 and HVTN704 (AMP) trials probing bnAb VRC01 for prevention. Fraction of participants with at least one C^1^/C^2^ sequence in the placebo and low and high dose of VRC01 treatment arms is shown. *p* value comparing placebo to pooled VRC01 arms was calculated using Fisher’s exact test. (E) Summary of findings on the effect of V1 Cys insertions.

**KEY RESOURCES TABLE:** 

REAGENT or RESOURCE	SOURCE	IDENTIFIER
Antibodies		
Monoclonal antibodies (HIV-1 bnAbs), see Table S3	N/A	N/A
Sheep Anti-HIV-1-p24 antibody	Aalto Bioreagents	Cat#D7320
Mouse monoclonal anti-HIV-1p24, Alkaline Phosphatase Conjugate	Aalto Bioreagents	Cat# BC1071-AP
Bacterial and virus strains		
XL10 gold Ultracompetent Cells (*Escherichia coli*)	Agilent Technologies	Cat#200315
Biological samples		
Human Plasma Samples	Swiss HIV Cohort Study, Zurich Primary HIV Infection Study	N/A
Chemicals, peptides, and recombinant proteins		
*Galanthus nivalis* lectin Agarose Resin	Vector Laboratories	Cat#AL-1243–5
Recombinant gp140 stabilized Env protein S5206G5–42wk-c3 SOSIP and DS-SOSIP, see Table S8	This paper	N/A
Recombinant p24	Aalto Bioreagents	Cat#AG6054
Critical commercial assays		
In-Fusion Snap Assembly Master Mix	Takara Bio Inc.	Cat#638949
QuikChange II XL Site-directed Mutagenesis Kit	Agilent Technologies	Cat#200521
Bright-Glo Luciferase Assay System	Promega	Cat#E2650
BirA biotinylation kit	Avidity	Cat#EC 6.3.4.15
PrimeScript One Step RT-PCR Kit	Takara Bio Inc.	Cat#RR057B
Deposited data		
Antibody Mediated Protection trial env sequences	Juraska et al.^45^ and available at https://atlas.scharp.org/cpas/project/HVTN%20Public%20Data/HVTN%20704%20HPTN%20085%20and%20HVTN%20703%20HPTN%20081%20AMP/begin.view	N/A
bnAb inducer *env* sequences	This paper	GenBank: PQ282666-PQ282697
Experimental models: Cell lines		
HEK 293-T cells	ATCC	CRL-3216, RRID: CVCL_0063
TZM-bl cells	NIH AIDS Reagent Program	ARP-8129, RRID: CVCL_B478
Expi293F cells	Thermo Fisher Scientific Inc.	A14528
Oligonucleotides		
Primers for site-directed mutagenesis, see Table S10	This paper	N/A
Primer MSR5: 5’-GCACTCAAGGCAAGCTTTATTGAGGCT-3’	Kouyos et al.^26^	N/A
Primer E27: 5’-AAGGCTTAGGCATYTCCTATG-3’	Kouyos et al.^26^	N/A
Primer MASC_A_InFusion: 5’-ACCGAGCTCGGATCGGCTTAGGCATCTCCTATGGCAGGAAGAA-3’	Kouyos et al.^26^	N/A
Primer MASC_M_InFusion: 5’-GCCACTGTGCTGGATTAGCCCTTCCAGTCCCCCCTTTTCTTTTA-3’	Kouyos et al.^26^	N/A
Recombinant DNA		
pNLluc-AM	Provided by A. Marozsan and J. P. Moore (Pugach P. et al.^51^)	N/A
pcDNA3.1/V5-His	Thermo Fisher Scientific Inc.	Cat#V81020
pCMV/R Avi	NIH AIDS Reagent Program (Barouch et al.^52^)	ARP-12279
Plasmid pcDNA3.1 encoding Furin	Provided by J. P. Moore (Sanders et al.^53^)	N/A
Env plasmids for pseudotyping, see Tables S2 and S4	N/A	N/A
Software and algorithms		
GraphPad Prism version 10.1.0	GraphPad Software	N/A
R version 4.3.1	www.r-project.org	N/A
EnvSeq	www.github.com/medvir/EnvSeq	N/A
ShoRAH	Zagordi et al.^54^ (https://cbg-ethz.github.io/shorah/)	N/A
Other		
Superdex 200 Increase 10/300 GL	Cytiva	Cat#28990944
Dynex MLX reader	Dynex Technologies	N/A
EnVision 2014 Multilabel Reader	Perkin Elmer Inc.	N/A

## Highlights

- HIV-1 Env from bnAb inducers is multi-bnAb resistant
- bnAb-inducer Envs frequently carry Cys insertions in V1
- V1 Cys insertions associate with the development of elite neutralization activity
- V1 Cys insertions change epitope accessibility on Env, augmenting bnAb escape
