# Rare twin cysteine residues in the HIV-1 envelope variable region 1 link to neutralization escape and breadth development

**DOI:** 10.1016/j.chom.2026.05.021

**Published:** 2026-06-02

**Authors:** Maria C. Hesselman, Marius Zeeb, Peter Rusert, Chloé Pasin, Jennifer Mamrosh, Samuel Kariuki, Ian Pichler, Michèle Sickmann, Masako M. Kaufmann, Daniel Schmidt, Nikolas Friedrich, Karin J. Metzner, Audrey Rindler, Herbert Kuster, Craig Adams, Ruwayhida Thebus, Michael Huber, Sabine Yerly, Karoline Leuzinger, Matthieu Perreau, Roger Koller, Günter Dollenmaier, Simona Frigerio, Dylan H. Westfall, Wenjie Deng, Allan C. deCamp, Michal Juraska, Srilatha Edupuganti, Nyaradzo Mgodi, Hugh Murrell, Nigel Garrett, Kshitij Wagh, James I. Mullins, Carolyn Williamson, Penny L. Moore, Huldrych F. Günthard, Roger D. Kouyos, Alexandra Trkola

In the originally published version of this article, an erroneous source data file was used to provide characteristics of the bnAb inducers studied, affecting Figures 1, 2, 5, and S1 as well as Tables S1, S2, and S6, including two text passages. The article has been corrected online, and original and corrected figures appear below. The corrections do not affect the key findings, interpretations, or conclusions of the study. The authors regret the error.

## Supplementary Material

Table S6

Table S2

Table S1

## Figures and Tables

**Figure 1.**
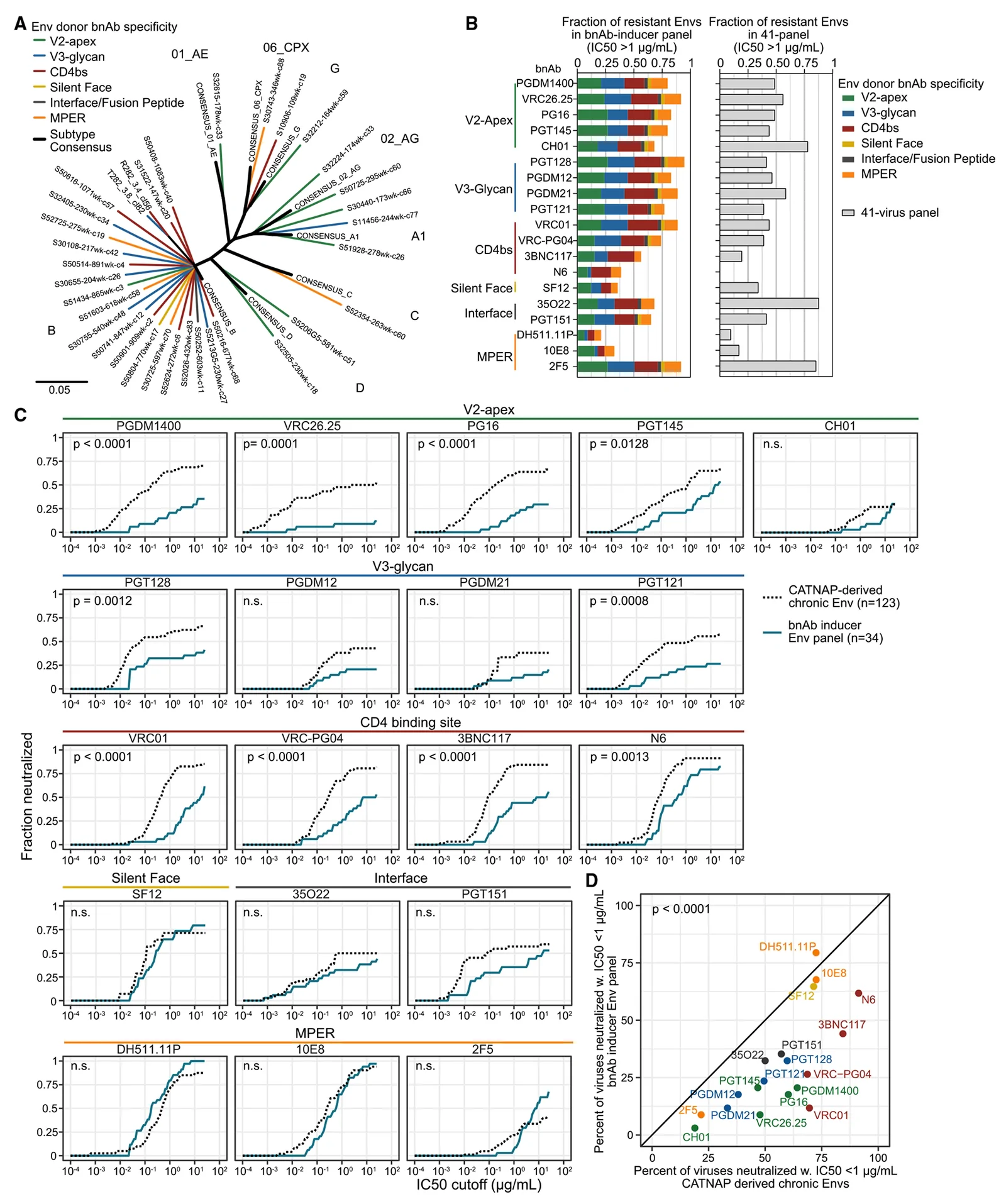
bnAb-inducer Envs have a generalized neutralization resistance (corrected)

**Figure 1.**
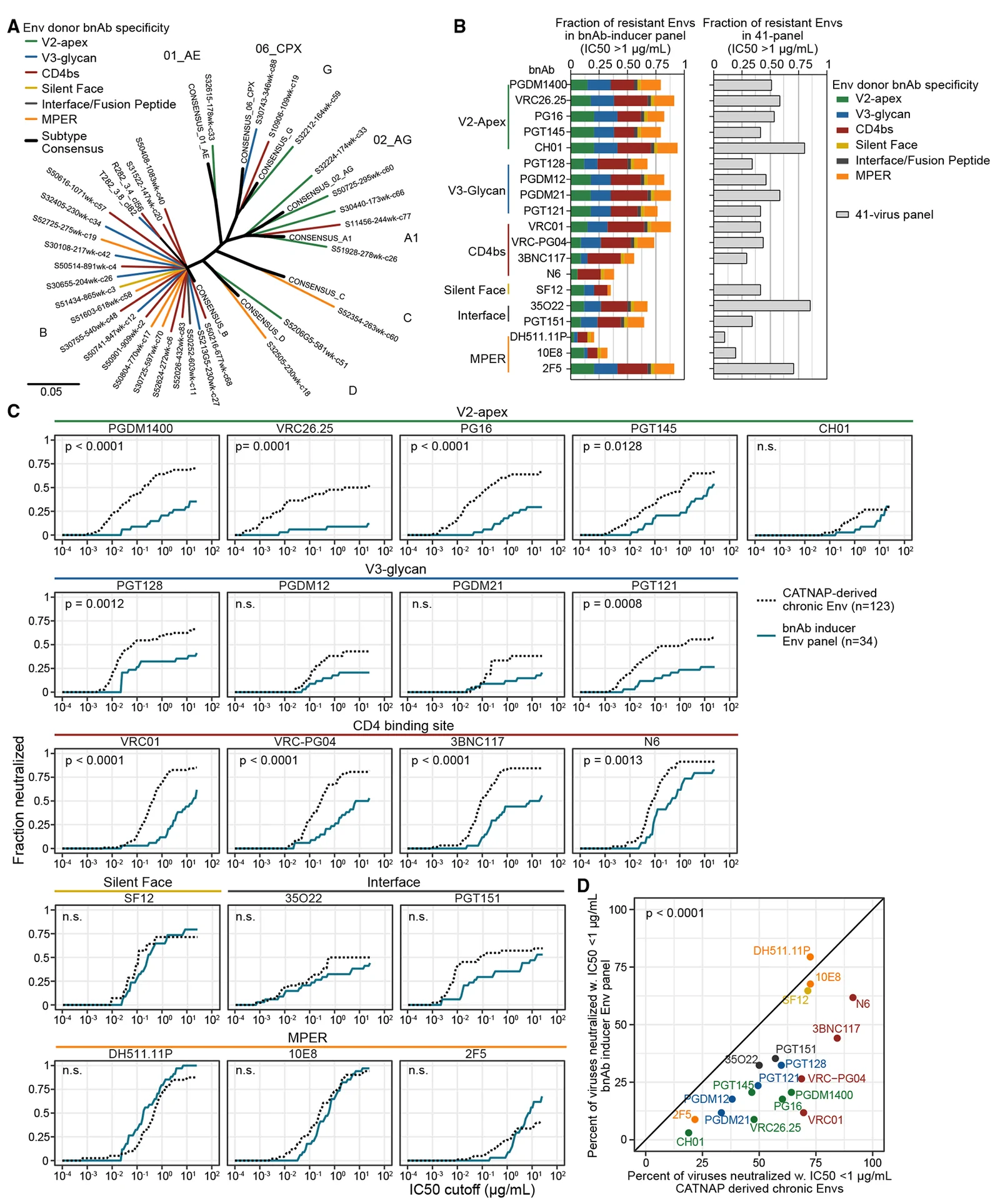
bnAb-inducer Envs have a generalized neutralization resistance (original)

**Figure 2.**
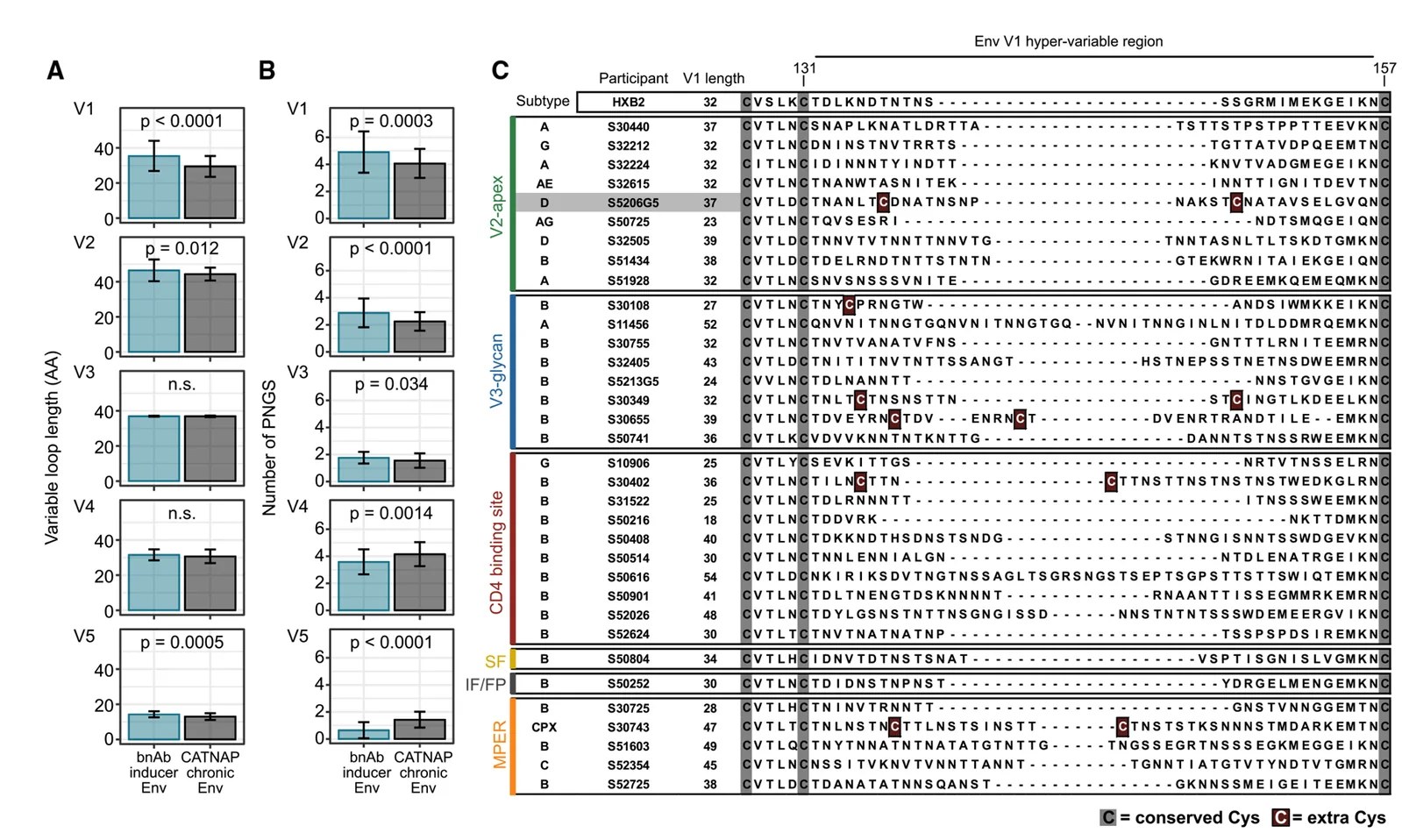
bnAb-inducer Envs have longer V1 loops with non-canonical cysteine residues (corrected)

**Figure 2.**
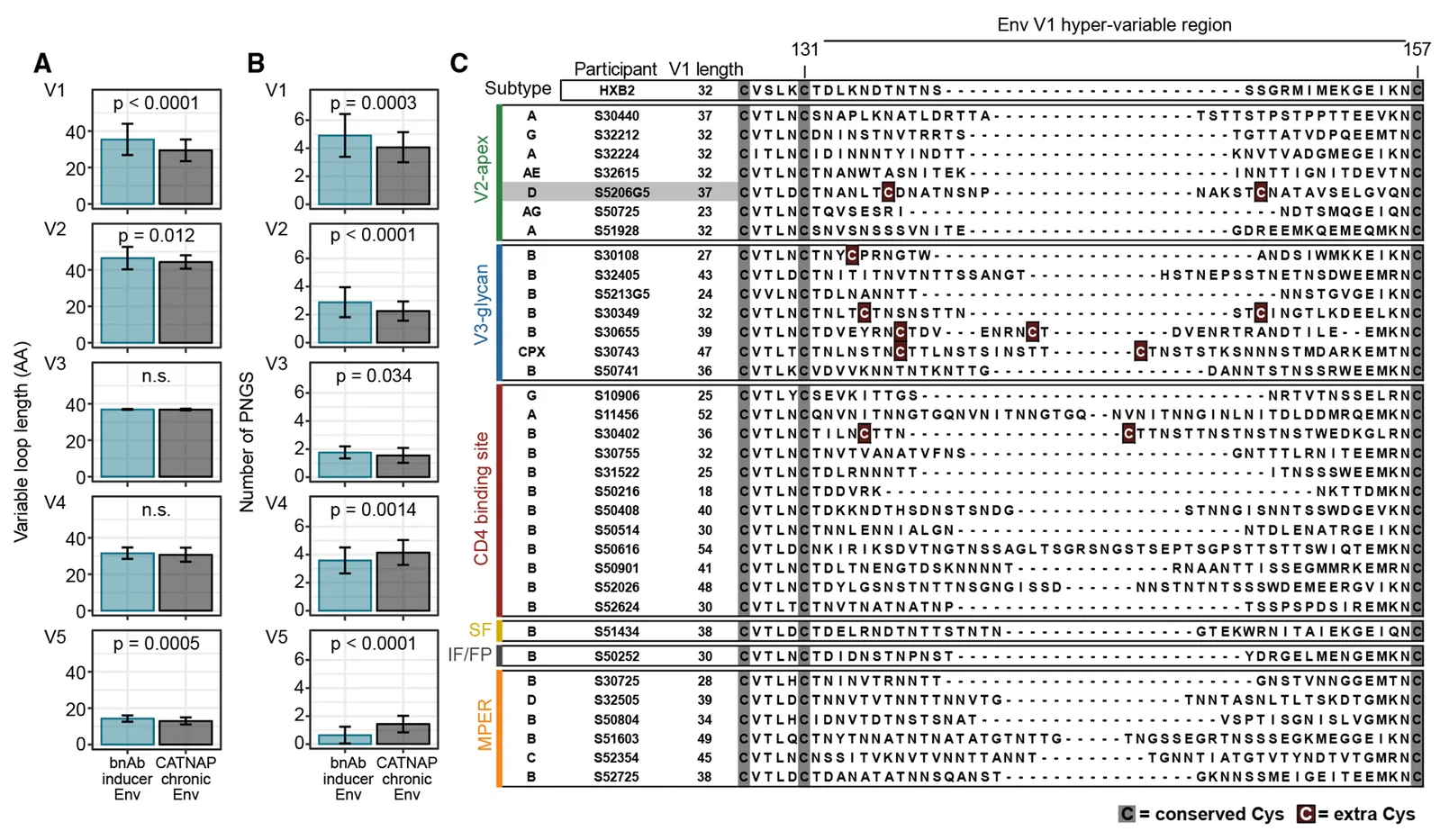
bnAb-inducer Envs have longer V1 loops with non-canonical cysteine residues (original)

**Figure 5.**
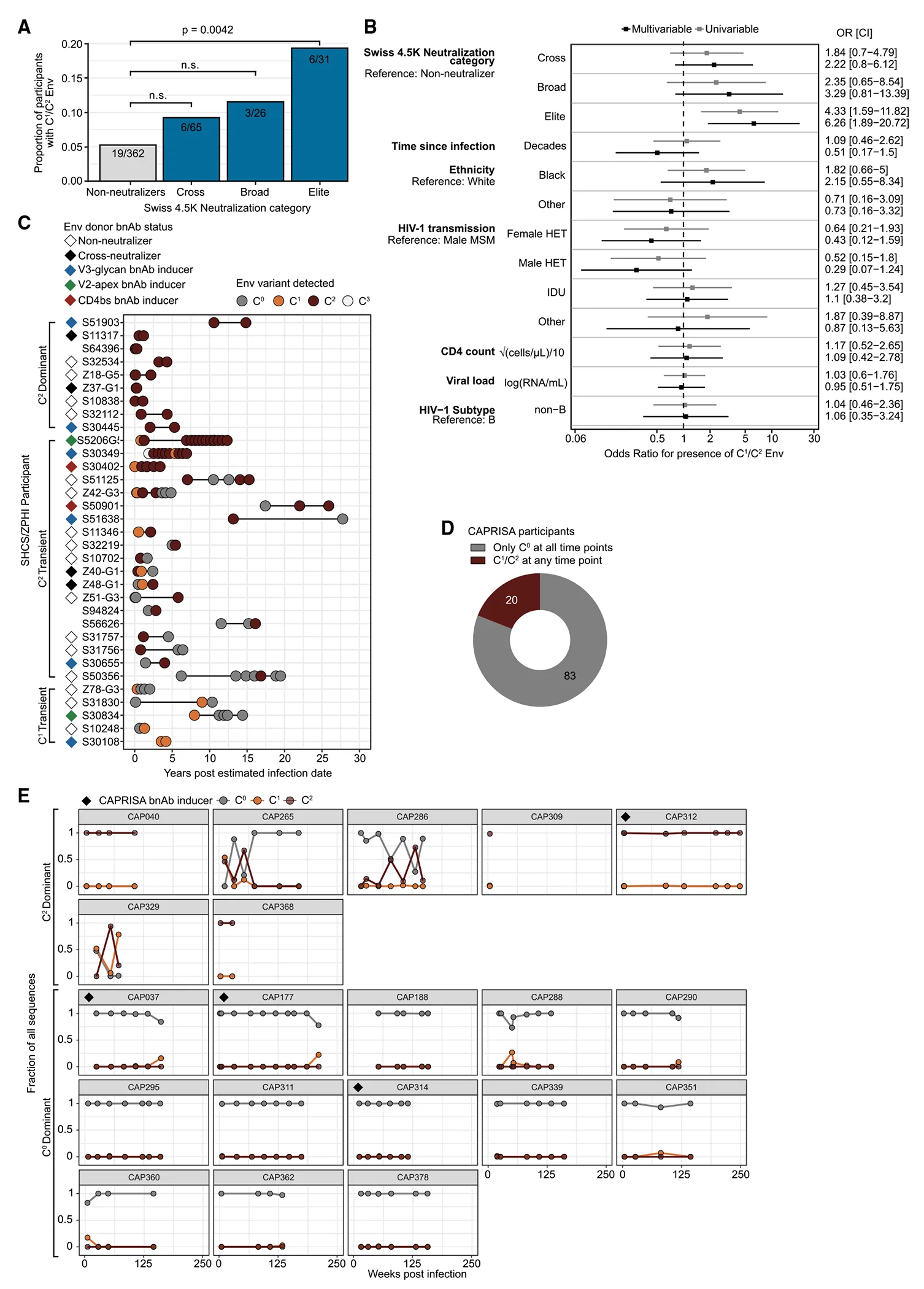
Non-canonical V1 Cys are enriched in bnAb inducers (corrected)

**Figure 5.**
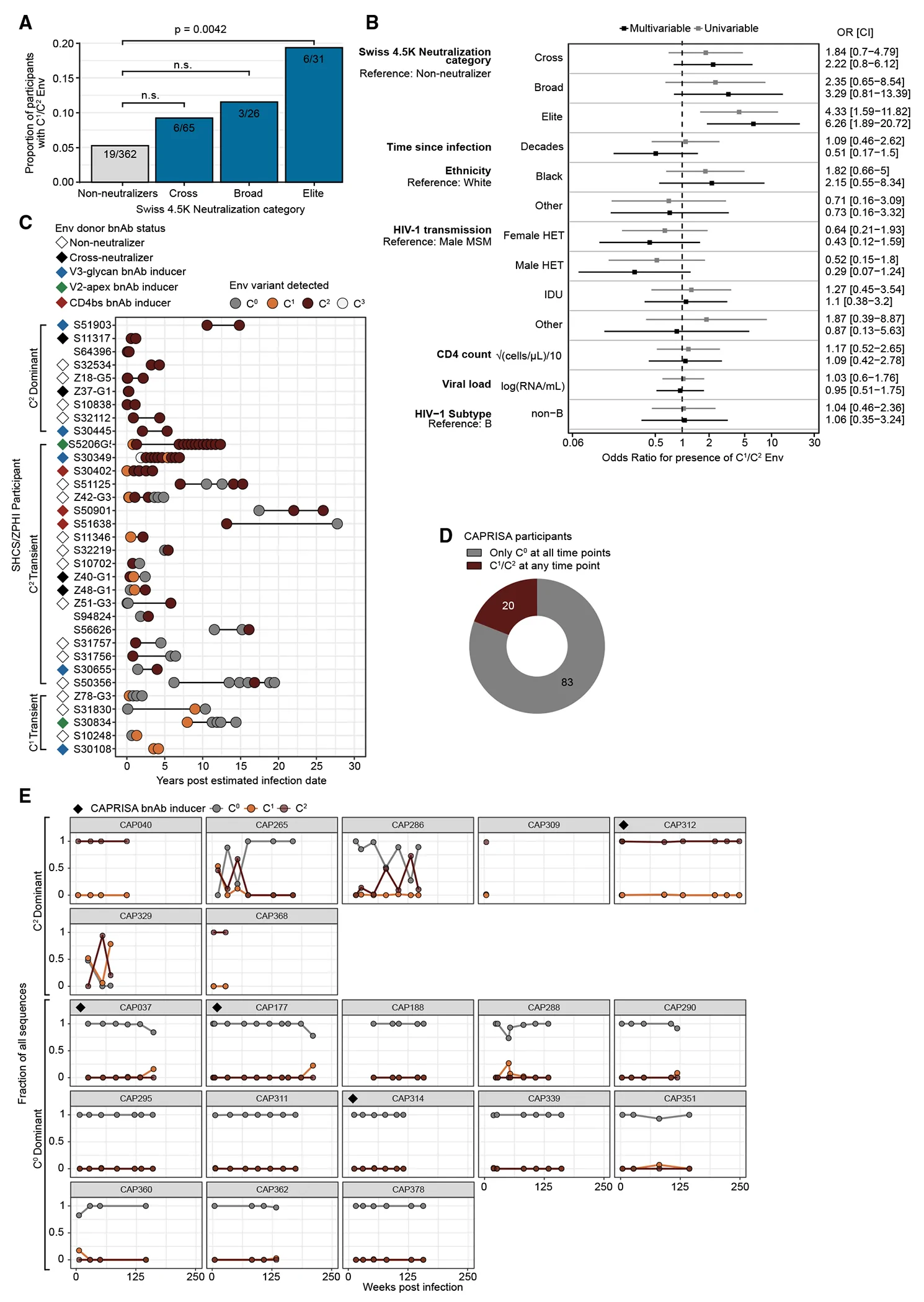
Non-canonical V1 Cys are enriched in bnAb inducers (original)

