# Supplementary material for: Rare twin cysteine residues in the HIV-1 envelope variable region 1 link to neutralization escape and breadth development
Source: Cell Host Microbe. Author manuscript; Available in PMC 2026 Sep 29. (PMC13621619; doi:10.1016/j.chom.2025.01.004)
Supplement: Supplementary file 1 [file NIHMS2116957-supplement-MMC1.pdf]

Cell Host & Microbe, Volume *33*

Supplemental information

Rare twin cysteine residues in the HIV-1 envelope variable region 1 link to neutralization escape and breadth development

Maria C. Hesselman, Marius Zeeb, Peter Rusert, Chloé Pasin, Jennifer Mamrosh, Samuel Kariuki, Ian Pichler, Michèle Sickmann, Masako M. Kaufmann, Daniel Schmidt, Nikolas Friedrich, Karin J. Metzner, Audrey Rindler, Herbert Kuster, Craig Adams, Ruwayhida Thebus, Michael Huber, Sabine Yerly, Karoline Leuzinger, Matthieu Perreau, Roger Koller, Günter Dollenmaier, Simona Frigerio, Dylan H. Westfall, Wenjie Deng, Allan C. deCamp, Michal Juraska, Srilatha Edupuganti, Nyaradzo Mgodi, Hugh Murrell, Nigel Garrett, Kshitij Wagh, James I. Mullins, Carolyn Williamson, Penny L. Moore, Huldrych F. Günthard, Roger D. Kouyos, and Alexandra Trkola

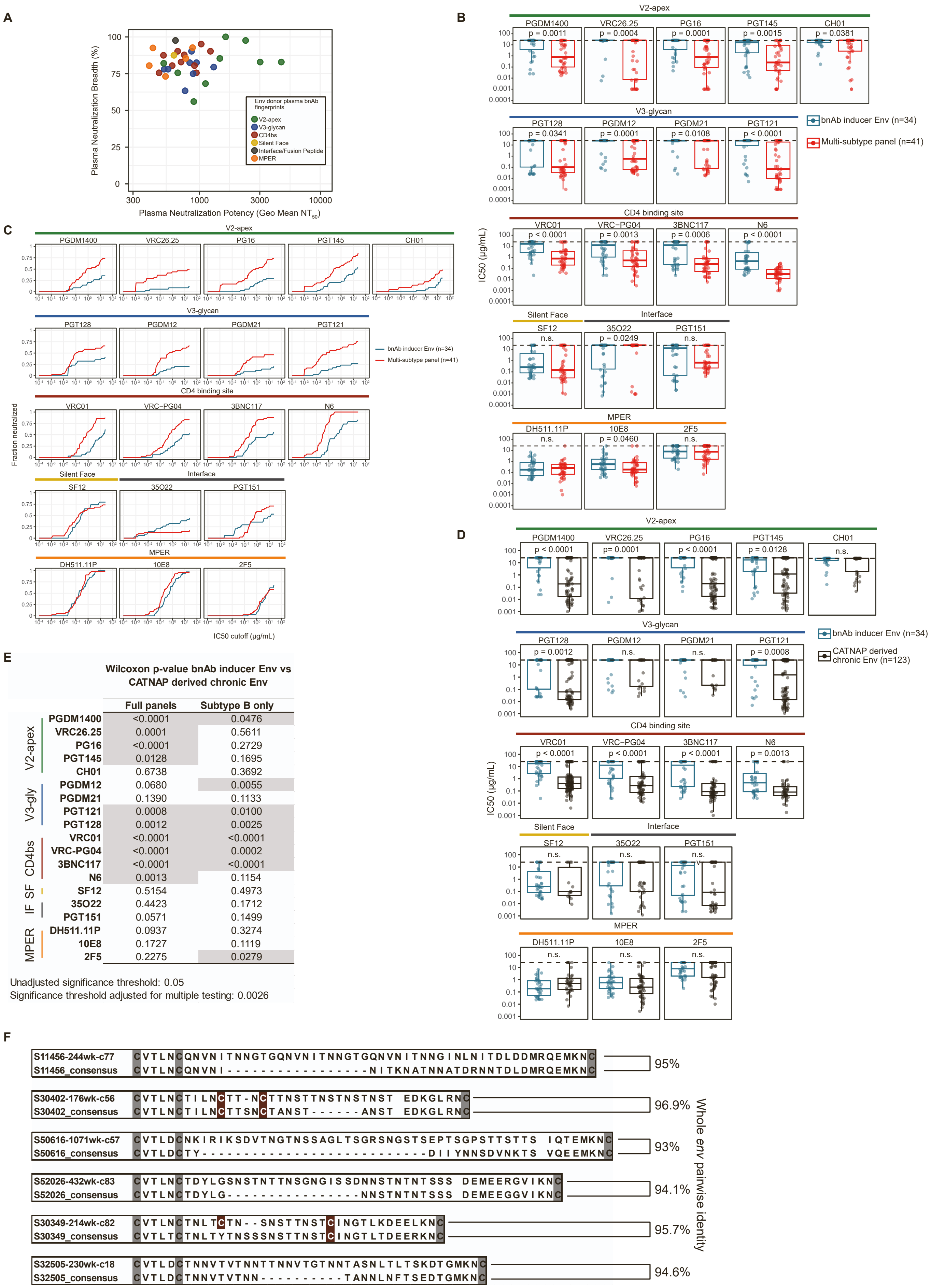

E

Wilcoxon p-value bnAb inducer Env vs CATNAP derived chronic Env

		Full panels	Subtype B only
V2-apex	PGDM1400	<0.0001	0.0476
	VRC26.25	0.0001	0.5611
	PG16	<0.0001	0.2729
	PGT145	0.0128	0.1695
	CH01	0.6738	0.3692
V3-gly	PGDM12	0.0680	0.0055
	PGDM21	0.1390	0.1133
	PGT121	0.0008	0.0100
	PGT128	0.0012	0.0025
CD4bs	VRC01	<0.0001	<0.0001
	VRC-PG04	<0.0001	0.0002
	3BNC117	<0.0001	<0.0001
	N6	0.0013	0.1154
SF	SF12	0.5154	0.4973
IF	35O22	0.4423	0.1712
	PGT151	0.0571	0.1499
MPER	DH511.11P	0.0937	0.3274
	10E8	0.1727	0.1119
	2F5	0.2275	0.0279

Unadjusted significance threshold: 0.05
Significance threshold adjusted for multiple testing: 0.0026

Figure S1. High neutralization resistance of bnAb-inducer Envs, related to Figures 1 and 2.
(A) Plasma neutralization breadth and potency of bnAb-inducers whose Envs are included in the bnAb-inducer Env panel (N = 34). Colors indicate the predicted plasma bnAb specificity. **(B)** IC50 of bnAbs against the bnAb-inducer Env panel (blue boxes) and the 41-virus multi-subtype Env panel (red boxes) (Table S2). Boxplots show the IC50 interquartile range, lines show the median IC50 and whiskers show the IC50 range. Points depict data of individual Envs. P-values indicate significance of the difference as assessed by Wilcoxon test. **(C)** Cumulative frequency distribution (breadth-potency curves) of the IC50 values of 19 bnAbs against the bnAb-inducer Env panel (blue) and 41-virus multi-subtype Env panel (red). **(D)** Comparison of bnAb IC50 against the bnAb-inducer Env panel (blue boxes) determined in the present study and bnAb IC50 reported in the CATNAP database for Envs isolated from chronic infection (CATNAP derived chronic Env). Boxplots show the IC50 interquartile range, lines show the median IC50 and whiskers show the IC50 range. Points depict data of individual Envs. P-values indicate significance of difference between the median IC50 of bnAbs in both Env panels as assessed by Wilcoxon test. **(E)** Wilcoxon test results depicted in (D) compared to the same analysis restricting to Subtype B Envs in in both Env panels. **(F)** Pairwise amino acid sequence alignment of the V1 regions of six bnAb inducer env sequences. Clonal sequences presented in Figure 2C were aligned to translated majority consensus sequences derived from NGS sequencing of plasma time points within one year of the clonal sequence sample. Pairwise identity was calculated on the whole env sequence.

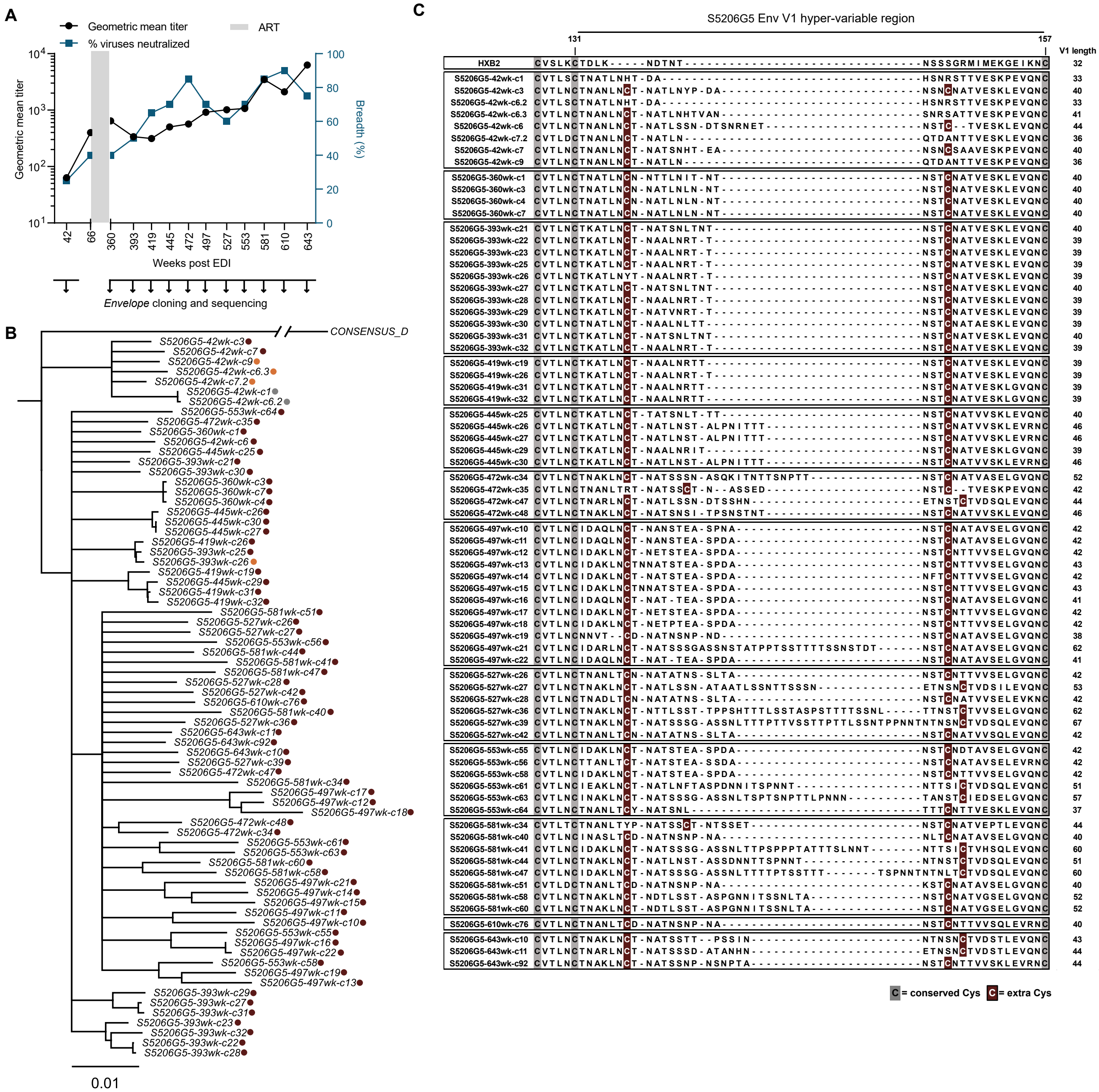

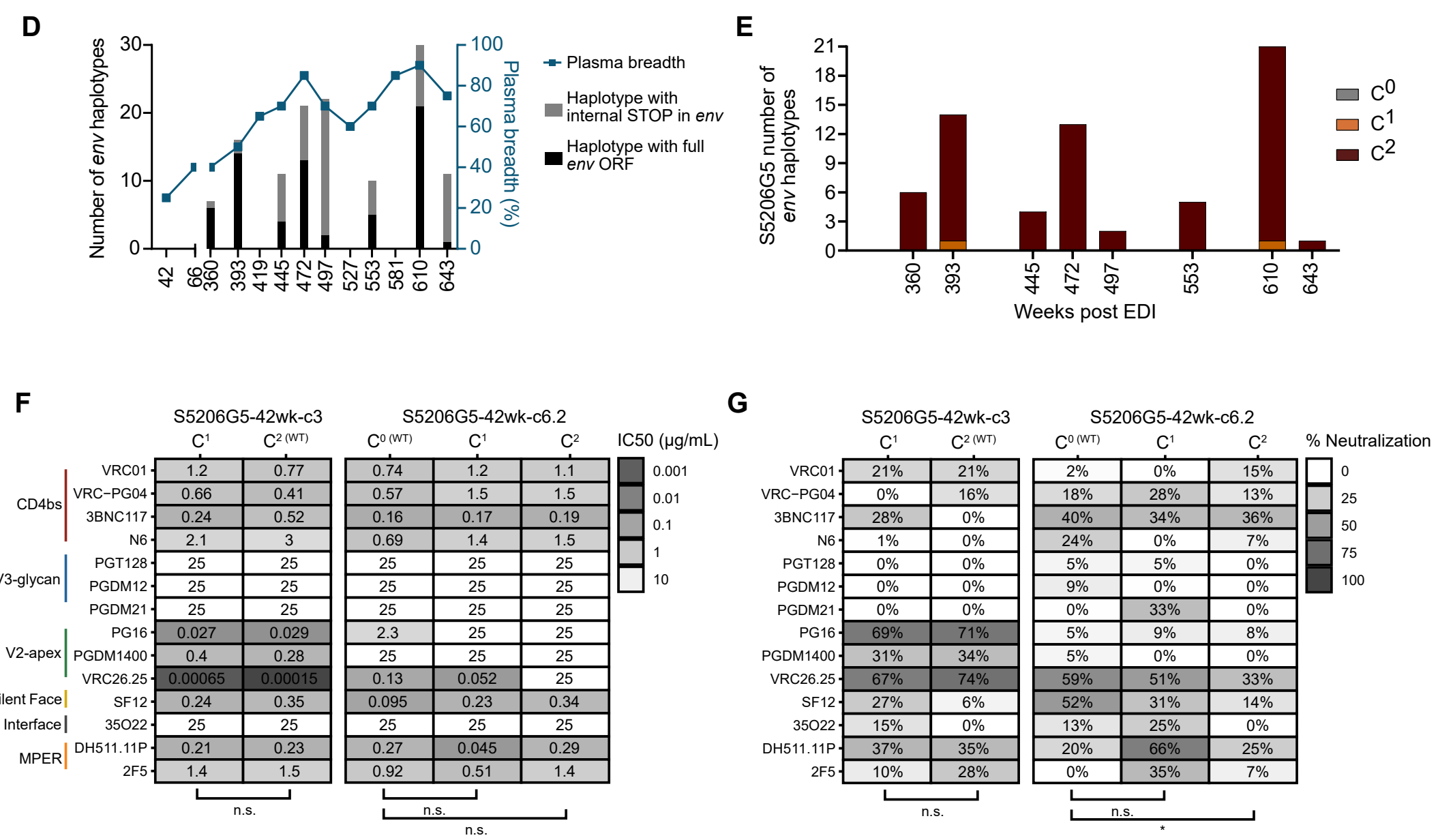

H Subtype distributions in CATNAP dataset used in Figure 3E

Subtype	C^0	C^2
01_AE	126	3
02_AG	19	0
02A1	5	0
06_cpx	1	0
07_BC	56	4
08_BC	16	0
12_BF	1	0
14_BG	1	0
21_A2D	3	0
41_CD	2	0
44_BF1	1	0
A1	69	0
A1C	13	1
A1CD	3	0
A1D	12	4
A1G	2	0
AD	1	0
B	458	44
BC	28	1
BG	1	0
C	299	12
CD	8	0
D	41	1
DU	1	0
F1	4	0
F2	1	0
G	16	1
H	1	0

Chi-squared p = 0.147

Figure S2. Modest impact of V1 Cys insertions on neutralization sensitivity, related to Figure 3.
(A) Longitudinal plasma neutralization breadth and potency of S5206G5. Right: y-axis (blue) shows percentage of viruses neutralized from a 19-virus multi-clade panel. Left: y-axis (black) the geometric mean NT50 against the neutralized viruses. The gray area indicates antiretroviral therapy (ART). The arrows below the plot indicate time points from which Env was sequenced. **(B)** Phylogenetic tree of clonal *env* nucleotide sequences of the V2-apex bnAb-inducer S5206G5 sampled 42-643 weeks post estimated infection date (EDI). C^0 (gray), C^1 (orange) and C^2 (burgundy) circles indicate sequences with 0, 1 or 2 extra V1 Cys, respectively. **(C)** Multiple sequence alignment of longitudinal clonal Env V1 hypervariable loop sequences from participant S5206G5. Conserved and extra V1 Cys residues are indicated by gray and burgundy shading, respectively. **(D)** Longitudinal env haplotypes from 8 time points of S5206G5 obtained from Nanopore sequences analyzed with ShoRAH haplotyping pipeline. Black bars indicate env haplotypes with a full env open reading frame (ORF) and gray bars indicate haplotypes with internal stop codons. Longitudinal plasma neutralization breadth is shown as blue line. **(E)** S5206G5 longitudinal env haplotypes' V1 Cys content. Only haplotypes with a full env ORF are plotted. **(F)** Neutralization sensitivity of S5206G5 week 42 wildtype and V1Cys mutant clones. Heat map depicts IC50 of 14 bnAbs. Results of paired t-test are depicted (ns = not significant). **(G)** Data depicted in (F) displaying percent neutralization at a low bnAb concentration (0.103 μg/mL). Results of paired t-test are depicted (ns = not significant). **(H)** Subtype distribution of CATNAP dataset analyzed in Figure 3E. Differences in subtype distributions between C^0 and C^2 Env subsets were assessed with Chi-squared test.

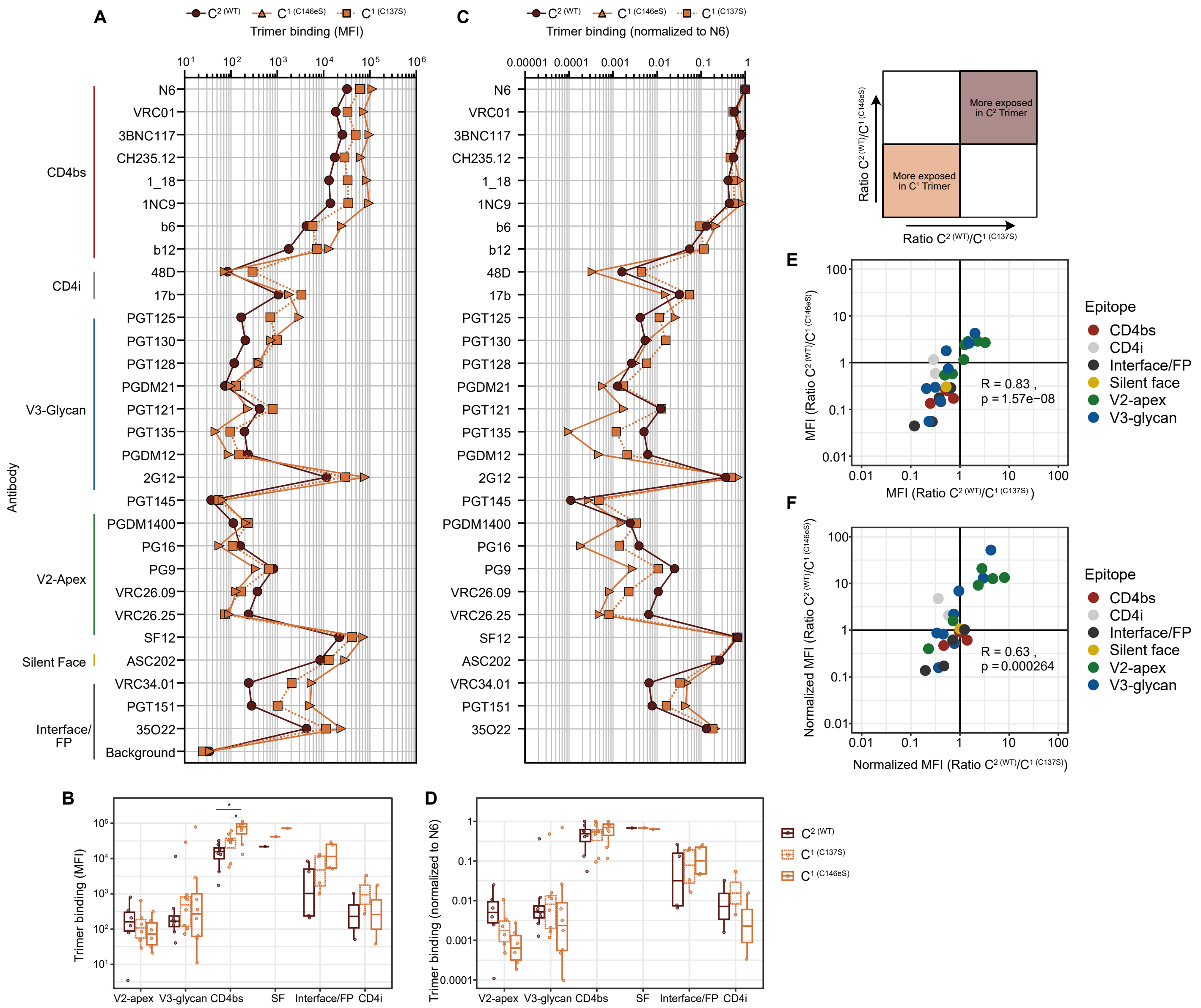

Figure S3. Antigenic properties of V1 Cys insertions, related to Figure 4.
(A) Luminex bead-based binding antibody multiplex assay (BAMA) of bnAbs (N = 29) against soluble SOSIP trimers of S5206G5-42wk-c3 Env variants: WT trimer (C^2), with a C137S mutation (C^1) and with a C146eS mutation (C^1). Data depict mean fluorescence intensity (MFI). **(B)** Boxplots depicting the MFI values from (A) with antibodies grouped by epitope. Differences in epitope exposure were assessed with Wilcoxon tests (*p-value < 0.05, **p-value < 0.01). **(C)** MFI values shown in (A) normalized to bnAb N6 which achieved the highest MFI signal in all data sets. **(D)** Boxplots depicting N6-normalized MFI values with antibodies grouped by epitope. **(E) (F)** Proportional differences in binding of C2 compared to C1 trimer versions based on data shown in (A) and (B) respectively. Each dot represents one bnAb, colors denote the bnAb epitope. Pearson correlation results of fold-change values are shown.

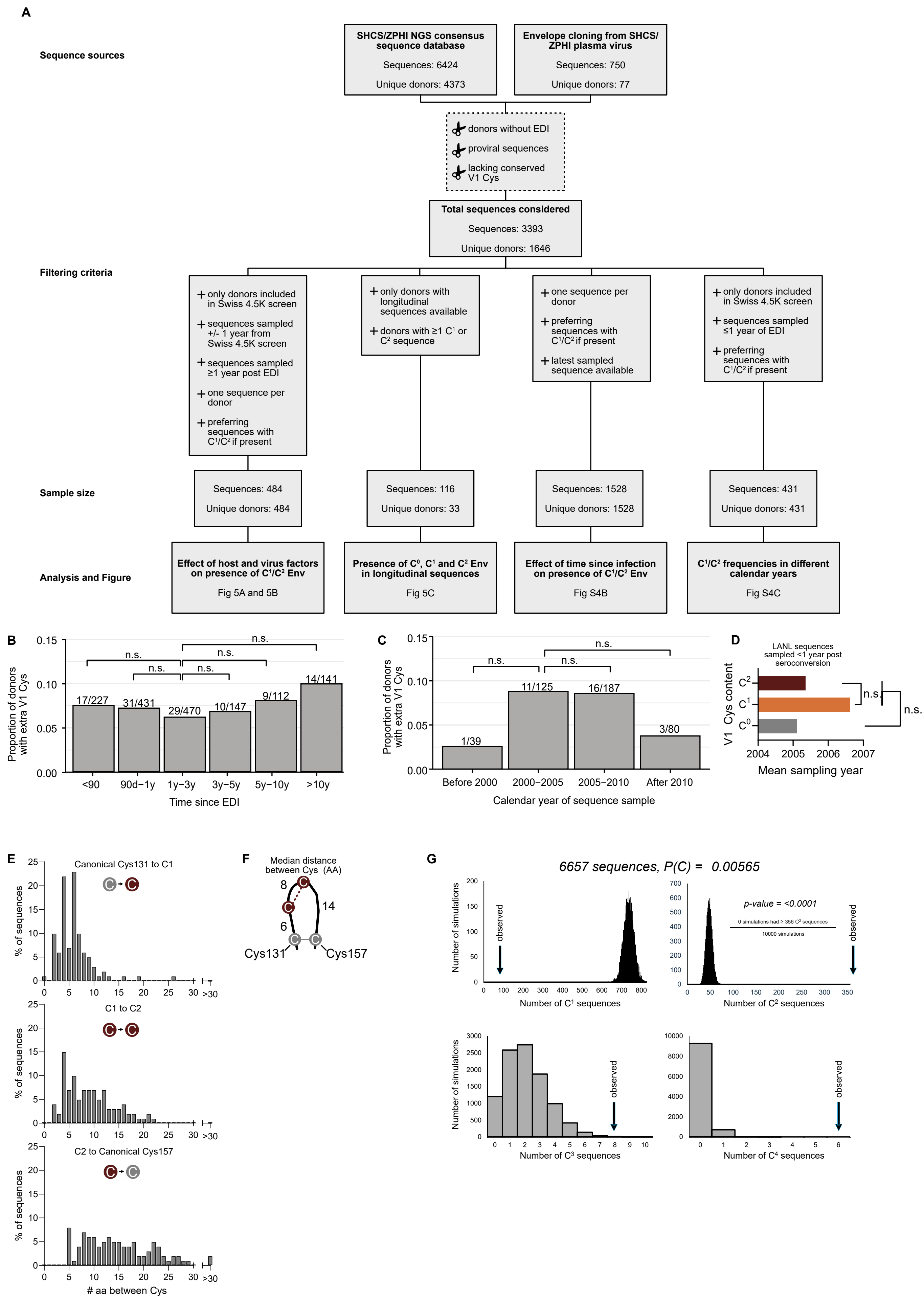

A
Sequence sources
SHCS/ZPHI NGS consensus sequence database
Sequences: 6424
Unique donors: 4373
Envelope cloning from SHCS/ ZPHI plasma virus
Sequences: 750
Unique donors: 77
donors without EDI
proviral sequences
lacking conserved V1 Cys
Total sequences considered
Sequences: 3393
Unique donors: 1646
Filtering criteria
+ only donors included in Swiss 4.5K screen
+ sequences sampled +/- 1 year from Swiss 4.5K screen
+ sequences sampled ≥1 year post EDI
+ one sequence per donor
+ preferring sequences with C¹/C² if present
+ only donors with longitudinal sequences available
+ donors with ≥1 C¹ or C² sequence
+ one sequence per donor
+ preferring sequences with C¹/C² if present
+ latest sampled sequence available
+ only donors included in Swiss 4.5K screen
+ sequences sampled ≤1 year of EDI
+ preferring sequences with C¹/C² if present
Sample size
Sequences: 484
Unique donors: 484
Sequences: 116
Unique donors: 33
Sequences: 1528
Unique donors: 1528
Sequences: 431
Unique donors: 431
Analysis and Figure
Effect of host and virus factors on presence of C¹/C² Env
Fig 5A and 5B
Presence of C⁰, C¹ and C² Env in longitudinal sequences
Fig 5C
Effect of time since infection on presence of C¹/C² Env
Fig S4B
C¹/C² frequencies in different calendar years
Fig S4C
B
Proportion of donors with extra V1 Cys
0.15
0.10
0.05
0.00
n.s.
n.s.
n.s.
n.s.
n.s.
17/227
31/431
29/470
10/147
9/112
14/141
<90
90d−1y
1y−3y
3y−5y
5y−10y
>10y
Time since EDI
C
Proportion of donors with extra V1 Cys
0.15
0.10
0.05
0.00
n.s.
n.s.
n.s.
1/39
11/125
16/187
3/80
Before 2000
2000−2005
2005−2010
After 2010
Calendar year of sequence sample
D
LANL sequences sampled <1 year post seroconversion
V1 Cys content
C²
C¹
C⁰
n.s.
n.s.
2004
2005
2006
2007
Mean sampling year
E
Canonical Cys131 to C1
C1 to C2
C2 to Canonical Cys157
% of sequences
0
5
10
15
20
25
30
>30
aa between Cys
F
Median distance between Cys (AA)
8
14
6
Cys131
Cys157
G
6657 sequences, P(C) = 0.00565
Number of simulations
observed
Number of C¹ sequences
p-value = <0.0001
0 simulations had ≥ 356 C² sequences
10000 simulations
Number of C² sequences
Number of C³ sequences
Number of C⁴ sequences

Figure S4. Researching extra V1 Cys at the population level, related to Figures 5 and 6.
(A) Flow chart describing the selection of SHCS, ZPHI and Swiss 4.5K participants researched for V1 sequences in analyses depicted in Figures 5 A-C, S4B and C. **(B)** Proportion of 1,528 SHCS/ZPHI participants with C^1/C^2 Envs depending on sampling time of the sequence in relation to the time since EDI. **(C)** Proportion of 431 SHCS/ZPHI participants with C^1/C^2 Envs depending on the calendar year of sequence sampling. **(D)** Mean sampling year of C^0, C^1 and C^2 sequences in the LANL HIV sequence database filtered web alignment. Differences assessed by t-test (n.s. = not significant). **(E)** C^2 sequences in the LANL HIV sequence database filtered web alignment were used to measure the distances between Cys residues in the V1 loop. Histograms depict the frequencies of sequences and their distances between canonical V1 Cys131 and the first Cys of the C^2 motif (top), the distance between the two Cys of the C^2 motif (middle) and the distance between the second Cys in the motif and the canonical C-terminal V1 Cys157 (bottom).
(F) Graphical depiction of the median distance between V1 Cys residues in V1 loops with the C^2 motif. **(G)** For each sequence in the LANL HIV sequence database filtered web alignment, an equal-length sequence was simulated. Whether or not a Cys was present at each amino acid position was randomly assigned based on the overall prevalence of Cys in all V1 hypervariable sequences (P(C)=0.00565). Histograms depict the number of C^1, C^2, C^3, and C^4 sequences across simulations. The observed number of sequences of each type is labeled as “observed”.

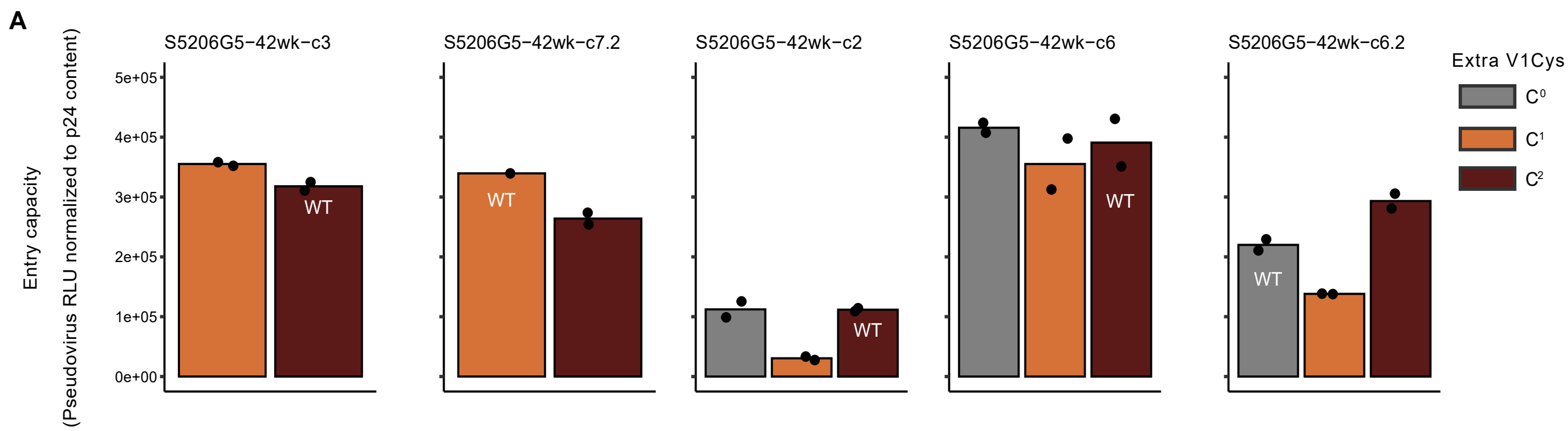

Figure S5. Entry capacity of V1 Cys variant Env, related to Figure 6.
Entry capacity of S5206G5-42wk Env V1 Cys variants was assessed by pseudovirus infection of TZM-bl cells normalized to p24 input. Relative light units (RLU) of emitted luciferase reporter activity, normalized to p24 content are shown as a measure of entry capacity.

Table S1. Plasma neutralization activity (NT50) (top) and predicted plasma specificity (bottom) of 34 Env donors included in the bnAb-inducer Env panel, related to Figure 1.

Plasma NT50 on 41-virus panel

Participant code (Rusert et al Ref.19)	Swiss4.5K Neutralization category	Subtype	Predicted bnAb specificity of donor plasma (defined by MSBP as in Ref. 19)	ZEnv16_1202_7	RHPA4259 clone 7	ZEnv91_0505_12	NAB13pre_cl_9	BaL_26	QH0692 clone 42	MG505_W0M_ENV_A2 A1	X2088	REJO4541 clone 67	Q769_h5
S10906	elite	G	CD4bs	2893	291	529	14207	2071	172	1815	383	894	1839
S30402	elite	B	CD4bs	1479	1432	148	1118	6105	273	848	40	821	710
S31522	elite	B	CD4bs	2079	1568	340	4204	7825	723	877	100	2180	782
S50216	elite	B	CD4bs	40	859	409	1331	1514	441	617	40	386	40
S50408	broad	B	CD4bs	1944	714	764	40	1340	40	376	488	1198	235
S50514	elite	B	CD4bs	701	1305	713	1509	3710	533	134	40	983	1153
S50616	elite	B	CD4bs	1241	685	879	2751	3596	1264	794	40	1743	1459
S50901	elite	B	CD4bs	902	387	285	2664	15465	2494	828	40	1259	486
S52026	cross	B	CD4bs	1026	1375	107	40	3214	174	741	105	1717	173
S52624	elite	B	CD4bs	1393	1303	162	6637	8297	802	668	40	549	1084
S30108	elite	B	V3-Gly	111	373	102	5308	7809	812	138	1879	1181	40
S11456	broad	A	V3-Gly	698	555	40	1368	9677	297	7778	74	955	40
S30755	broad	B	V3-Gly	2374	1474	187	40	8959	790	40	254	586	434
S32405	elite	B	V3-Gly	232	2012	136	188	3080	652	451	40	552	40
S5213G5	elite	B	V3-Gly	40	528	40	40	3278	140	106	2023	280	40
S30349	elite	B	V3-Gly	1025	2943	382	527	2422	231	572	40	1510	1772
S30655	broad	B	V3-Gly	178	2806	40	742	4254	1272	236	94	372	40
S50741	broad	B	V3-Gly	40	1034	40	131	4286	381	116	1420	475	40
S30440	elite	A	V2-Apex	929	472	40	885	511	113	2895	40	635	1823
S32212	elite	G	V2-Apex	102	40	40	8905	133	40	970	40	134	3454
S32224	elite	A	V2-Apex	588	327	552	4929	5040	152	5739	272	588	2988
S32615	elite	AE	V2-Apex	334	91	40	613	381	129	202	40	250	120
S5206G5	elite	D	V2-Apex	1064	146	40	340	1781	4350	1906	40	656	40
S50725	elite	AG	V2-Apex	841	507	216	14167	3312	322	6958	40	457	898
S32505	elite	D	V2-Apex	117	55	113	1022	907	625	801	132	356	40
S51434	broad	B	V2-Apex	6146	5644	5710	1011	26278	762	1466	396	9281	671
S51928	elite	A	V2-Apex	663	114	40	205	469	102	516	40	123	272
S30725	elite	B	MPER	3198	371	40	734	1769	929	132	40	1545	40
S30743	cross	CPX	MPER	86	230	40	40	1680	104	167	41	223	55
S51603	cross	B	MPER	702	928	40	981	2250	1423	468	40	2361	125
S52354	cross	C	MPER	75	510	418	320	861	465	116	40	181	167
S52725	elite	B	MPER	922	748	225	4175	3431	532	797	539	728	40
S50252	elite	B	IF_FP	4350	674	40	233	11719	151	619	316	1784	453
S50804	broad	B	SF	1470	310	251	596	1494	111	706	122	1400	399

Participant code (Rusert et al Ref.19)	C1080_c03	ZEnv07_0504_15	NAB5pre_cl_1	CRF02_cl_250	HIV-001428-2	TRO clone 11	DU422	JR-CSF	HIV_25925_2	ZM106F.PB9	53M12	PVO clone 4	KER2018.11	ZM214M.PL15	0815_v3_c3	ZEnv32_0111_5	CNE5
S10906	3786	464	103	5181	3786	2141	761	1829	2125	2353	208	458	3851	40	5443	119	137
S30402	227	122	145	159	3953	382	40	566	40	179	135	479	40	40	956	272	176
S31522	962	40	508	217	1599	1883	150	891	483	104	505	1014	40	40	1415	2601	40
S50216	1964	423	40	342	311	665	103	3356	543	332	164	1769	355	40	186	118	40
S50408	840	957	465	381	281	931	381	569	594	427	163	812	128	436	40	490	149
S50514	605	387	194	904	1454	2420	40	454	1422	1759	40	1141	40	40	176	40	124
S50616	3726	527	148	577	2231	1296	661	703	40	655	500	328	389	1403	1175	5630	589
S50901	2180	152	40	212	3158	395	156	698	409	606	537	191	135	356	602	315	40
S52026	301	868	106	434	2354	1183	422	4261	2816	696	386	742	40	40	257	195	40
S52624	444	439	205	679	2194	1217	40	3627	40	338	662	881	339	40	2069	694	40
S30108	1218	40	265	487	372	2595	342	1222	1708	623	40	223	40	40	303	308	40
S11456	329	40	630	6391	2166	2078	1018	1855	161	1300	1948	357	180	40	851	40	100
S30755	253	494	183	596	162	3844	40	2557	845	531	285	3148		40	117	40	251
S32405	269	439	40	845	179	1115	40	1968	1373	506	40	455	40	162	104	199	40
S5213G5	40	40	226	4251	523	2985	742	4705	5353	2377	40	155	40	40	352	40	40
S30349	507	265	2434	3711	5668	1691	630	6989	1060	1431	111	885	502	181	1353	40	40
S30655	982	224	8563	25556	700	6837	681	6183	1973	310	40	1167	1414	833	1291	159	
S50741	90	1220	399	6518	1043	2900	829	1362	3026	7023	40	117	134	40	204	40	40
S30440	8547	400	40	13396	2433	195	40	1002	2114	530	560	226	31059	40	40	2413	6853
S32212	2399	40	40	8961	742	40	40	1238	1121	978	510	40	2543	40	40	40	2141
S32224	144035	497	259	20262	6270	456	937	18189	2404	406	612	286	55494	40	987	21376	4234
S32615	14799	40	40	1270	444	120	40	246	1034	259	401	40	1779	40	40	1319	2826
S5206G5	189008	40	174	39189	1808	851	40	259	4701	576	25479	200	12580	40	163	1775	200000
S50725	4545009	169	215	105847	4444	912	40	746	1703	215	40	40	12850	40	465	4589	112044
S32505	1586	40	40		758	603	205	175	1060	134	408	40		129	351	284	3484
S51434	2444	1704	2844	839	2721	2458	602	9150	581	861	1611	1651	341	425	411	210	644
S51928	87571	40	40	1796	858	109	40	5965	400	40	254	40	7554	40	40	1755	2894
S30725	1148	283	311	810	40	456	162	2545	108	242	257	40	1059	40	40	266	40
S30743	3123	133	511	10245	730	1181	689	169	545	3081	40	40	1051	40	345	67	142
S51603	5639	1568	283	860	40	1255	154	975	304	40	40	698	591	830	150	469	40
S52354	188	171	419	628	367	402	280	452	1099		218	113	38221	199	40	1606	40
S52725	6913	605	283	729	219	1571	542	903	1296	418	227	420	1501	40	332	1868	40
S50252	629	872	475	1167	171	621	499	2576	4704	1021	225	283	670	517	220	178	118
S50804	2381	239	411	4185	1331	675	1264	1693	40	318	508	40		193	225	342	574

Participant code (Rusert et al Ref.19)	CRF02_cl_252	ZM249M.PL1	NAB9pre_cl_106	CAP88.6m.c10	JR-FL	ZM233M.PB6	CAP45_2_00_G3	ZEnv92_1008_8	Q842_d12_PNS70d A1	Du156.12	BG505_W6M_ENV_A5_T332N	WITO4160 clone 33	CNE59	Q23_17	Breadth	Potency (Geometric Mean NT50)
S10906	194	1155	242	112	3229	40	875	919	9635	1744	3330	2126	105	3704	95%	1030.38
S30402	40	121	84	40	2373	40	40	40	1959	1136	788	254	205	724	76%	480.286
S31522	40	1052	148	40	4625	40	40	625	5278	1268	1119	919	798	1220	80%	912.13
S50216	40	422	40	464	2324	279	960	1311	450	1660	1258	290	4031	731	80%	609.21
S50408	236	40	130	40	889	1036	226	747	578	724	1099	201		3313	88%	520.454
S50514	327	329	851	117	2099	1843	40	154	1244	1439	2144	786	1169	429	83%	715.892
S50616	40	766	359	40	6322	128	1950	1133	4182	6473	2601	1238	17037	2059	90%	1217.68
S50901	40	179	1304	188	1941	139	1901	1019	1276	957	780	549	844	3750	90%	672.999
S52026	348	590	839	4821	4990	654	303	615	914	37144	1805	40	341	1350	88%	758.075
S52624	40	198	340	40	8077	40	40	473	2559	1348	1642	40	711	1226	76%	943.614
S30108	391	461	462	102	2216	109	40	40	40	793	744	104	888	1789	78%	565.873
S11456	2627	359	40	202	2254		131	251	4832	4031	1183	40	573	40859	83%	946.104
S30755	276	731	900	40	4413	1463	40	1325	3780	3021	9705	447	149	3210	83%	836.98
S32405	194	618	671	395	1021	143	40	1257	238	918	1238	40	713	4554	78%	527.313
S5213G5	597	224	226	136	1349	339	40	40	316	1860	1366	40	40	13921	63%	769.528
S30349	3331	100	107	1007	2814	161	40	131	2365	1887	929	782	179	7605	90%	864.588
S30655	17184	40	40	3550	1763		40	1313	40	855	2933	40	986	2846	79%	1291.24
S50741	2322	467	3187	40	1556	1931	113	40	40	6025	1250	379		7883	75%	889.856
S30440	1270	209	173	152	131	7059	12201	2759	8886	1730	2904	4826	1851	37204	85%	1443.93
S32212	40	2023	40	40	143	40	40	166	1370	255	237	40	188	37589	56%	900.858
S32224	348	1650	577	2406	443	5563	20630	8094	9353	2252	5563	14006	2221	14844	98%	2301.47
S32615	40	248	374	40	135	712	3354	4827	3111	1131	783	241	7897	6708	76%	673.934
S5206G5	40	780	1079	284	630	8107	148845	6757	117642	7390	5508	214	22996	200000	83%	2992.37
S50725	40	7498	432	40	883	59393	9588	15805	92277	31593	24993	838	8919	2083875	83%	4388.19
S32505	40	363	460	40	113	1306	13055	1446	40	2233	1239	155	1234	3667	82%	522.732
S51434	228	181	811	683	5942	1416	5069	3110	5413	929	3951	6613	2218	4876	100%	1601.81
S51928	104	40	40	40	40	7717	7138	5730	2460	973	1336	10559	399	10439	68%	1113.18
S30725	40	40	425	173	107	859	1245	820	40	891	113	1275	738	1710	73%	540.205
S30743	99	56	81	1078	222	40	40	2730	40	1804	270	116	1791	8103	80%	402.178
S51603	206	244	430	1606	614	273	991	793	189	2662	695	2339	6753	7914	85%	780.016
S52354	263	291	2088	1116	580	444	672	336	167	1451	266	105	1244	834	93%	429.883
S52725	105	915	608	208	1986	513	348	3215	1845	4136	1443	756	3034	9007	93%	881.016
S50252	254	401	331	1353	752	2197	3102	109	507	1077	1467	2312	118	1235	98%	646.684
S50804	258	40	4426	298	1822	40	336	1228	1069	155	638	349	40	6683	88%	628.906

Spearman correlation coefficients of plasma and reference bnAbs

Participant	Neutralizati	Subtype	Predicted bnAb specificity of donor plasma (defined by MSBP as in Ref. 19)	CD4bs: VRC01	VRC-PG04	3BNC117	NIH45-46	N6	1NC9	N49P7	CH235.12	1-18	VRC.CH31	BG24	8ANC134	HJ16	IOMA	b12
S10906	elite	G	CD4bs	0.53	0.31	0.52	0.44	0.46	0.34	0.49	0.35	0.71	0.49	0.53	0.41	-0.10	-0.03	-0.20
S30402	elite	B	CD4bs	0.70	0.45	0.67	0.72	0.56	0.51	0.74	0.70	0.75	0.68	0.77	0.63	0.10	0.27	0.12
S31522	elite	B	CD4bs	0.58	0.35	0.60	0.59	0.55	0.38	0.71	0.59	0.65	0.63	0.66	0.51	-0.06	0.21	0.07
S50216	elite	B	CD4bs	0.26	0.29	0.47	0.35	0.15	0.32	0.37	0.26	0.42	0.25	0.55	0.09	-0.05	0.41	0.29
S50408	broad	B	CD4bs	0.08	0.15	0.35	0.09	-0.01	0.29	0.27	0.21	0.25	0.15	0.16	0.22	-0.03	0.37	0.01
S50514	elite	B	CD4bs	0.49	0.41	0.44	0.52	0.35	0.37	0.58	0.35	0.68	0.42	0.55	0.28	0.05	0.25	-0.05
S50616	elite	B	CD4bs	0.41	0.24	0.46	0.36	0.45	0.30	0.56	0.51	0.44	0.38	0.59	0.33	-0.06	0.24	0.23
S50901	elite	B	CD4bs	0.44	0.33	0.41	0.41	0.42	0.27	0.58	0.37	0.49	0.43	0.64	0.44	-0.09	0.32	0.09
S52026	cross	B	CD4bs	0.38	0.56	0.45	0.47	0.35	0.41	0.45	0.45	0.41	0.32	0.42	0.21	0.05	0.27	0.13
S52624	elite	B	CD4bs	0.61	0.42	0.62	0.59	0.47	0.53	0.64	0.60	0.77	0.62	0.69	0.61	0.02	0.41	0.05
S30108	elite	B	V3-Gly	0.18	0.14	0.13	0.18	0.33	0.05	0.40	0.08	0.48	0.21	0.30	0.23	-0.04	0.13	0.11
S11456	broad	A	V3-Gly	0.41	0.26	0.34	0.37	0.45	0.26	0.37	0.38	0.56	0.36	0.50	0.37	-0.09	0.08	-0.05
S30755	broad	B	V3-Gly	0.39	0.48	0.51	0.34	0.08	0.42	0.30	0.31	0.43	0.39	0.27	0.28	-0.06	0.42	-0.02
S32405	elite	B	V3-Gly	0.31	0.47	0.37	0.32	0.19	0.33	0.31	0.27	0.41	0.35	0.33	0.09	0.16	0.44	0.12
S5213G5	elite	B	V3-Gly	0.25	0.34	0.18	0.23	0.40	0.17	0.24	0.26	0.43	0.21	0.22	0.23	-0.03	0.05	0.03
S30349	elite	B	V3-Gly	0.47	0.36	0.51	0.49	0.32	0.40	0.42	0.41	0.62	0.47	0.50	0.46	-0.04	0.13	0.05
S30655	broad	B	V3-Gly	0.00	-0.03	0.13	-0.01	-0.13	0.03	-0.06	-0.02	0.23	0.07	0.05	0.05	0.18	0.06	0.09
S50741	broad	B	V3-Gly	0.16	0.23	0.06	0.08	0.25	0.06	0.08	0.08	0.32	0.05	0.09	0.02	-0.07	0.03	0.03
S30440	elite	A	V2-Apex	0.13	0.04	0.22	-0.01	0.08	0.04	0.01	0.18	0.05	0.05	0.07	-0.03	-0.21	-0.19	-0.40
S32212	elite	G	V2-Apex	0.41	0.36	0.33	0.36	0.25	0.26	0.29	0.21	0.43	0.29	0.32	0.28	-0.17	-0.02	-0.25
S32224	elite	A	V2-Apex	0.11	-0.02	0.22	-0.01	0.25	-0.08	0.05	0.20	0.09	0.05	0.10	-0.03	-0.36	-0.16	-0.19
S32615	elite	AE	V2-Apex	0.11	0.11	0.13	-0.01	0.16	0.10	0.12	0.15	0.09	0.00	0.11	-0.08	-0.18	-0.10	-0.26
S5206G5	elite	D	V2-Apex	0.07	0.15	0.12	-0.04	0.07	0.11	0.02	0.13	0.05	0.00	0.07	-0.09	-0.08	-0.06	-0.22
S50725	elite	AG	V2-Apex	0.25	0.18	0.27	0.11	0.28	0.11	0.21	0.24	0.26	0.19	0.26	0.00	-0.14	-0.09	-0.16
S32505	elite	D	V2-Apex	0.05	0.04	-0.01	-0.08	0.12	-0.04	0.06	0.07	0.00	-0.04	0.10	-0.19	-0.02	-0.12	-0.03
S51434	broad	B	V2-Apex	0.31	0.24	0.49	0.34	0.14	0.34	0.47	0.29	0.39	0.39	0.49	0.34	-0.10	0.42	0.23
S51928	elite	A	V2-Apex	0.07	-0.05	0.25	-0.06	0.05	0.04	0.04	0.21	0.04	0.04	0.06	-0.01	-0.23	-0.13	-0.22
S30725	elite	B	MPER	-0.11	-0.18	0.07	-0.15	-0.04	-0.01	0.05	-0.04	0.03	-0.13	0.03	-0.03	-0.12	0.02	0.14
S30743	cross	CPX	MPER	0.14	0.17	0.26	0.08	0.06	0.19	0.03	0.18	0.25	0.04	0.10	-0.09	0.16	-0.06	0.07
S51603	cross	B	MPER	0.00	-0.05	0.19	0.02	-0.05	0.01	0.13	0.00	0.10	0.00	0.20	-0.04	0.04	0.21	0.29
S52354	cross	C	MPER	-0.07	0.10	0.04	-0.09	0.09	0.01	-0.04	-0.05	0.01	-0.19	-0.01	-0.24	-0.18	0.09	0.13
S52725	elite	B	MPER	0.23	0.14	0.38	0.15	0.25	0.16	0.30	0.23	0.40	0.22	0.30	0.08	-0.16	0.19	-0.02
S50252	elite	B	IF_FP	0.09	0.07	0.22	0.14	0.20	0.01	0.23	0.21	0.19	0.02	0.21	0.11	0.01	-0.06	0.12
S50804	broad	B	SF	0.22	0.16	0.23	0.08	0.32	0.00	0.22	0.12	0.28	0.26	0.15	0.33	-0.27	0.25	-0.08

Participant	V3-Gly: 10_1074	PGT121	PGT128	VRC41.01	PGDM12	PGDM21	PGT125	PGT130	PGT135	2G12
S10906	0.11	0.19	0.21	0.18	0.06	0.02	0.21	0.12	0.25	0.13
S30402	0.00	0.15	0.18	0.03	0.01	0.12	0.29	0.15	0.18	0.27
S31522	-0.05	0.08	0.12	-0.03	-0.07	0.07	0.20	0.14	0.17	0.23
S50216	0.00	-0.04	0.32	0.08	0.10	0.10	0.30	-0.01	0.18	0.34
S50408	0.18	0.09	0.27	0.10	0.22	0.09	0.26	0.25	0.14	0.28
S50514	0.33	0.15	0.27	0.22	0.41	0.14	0.03	-0.13	0.39	0.37
S50616	-0.28	-0.18	-0.12	-0.21	-0.27	-0.17	0.06	0.07	-0.14	0.07
S50901	-0.04	0.04	0.02	-0.12	-0.14	-0.06	0.11	-0.01	0.02	0.05
S52026	0.42	0.43	0.52	0.37	0.45	0.28	0.29	0.22	0.29	0.24
S52624	0.06	0.17	0.27	0.06	0.07	0.12	0.35	0.17	0.27	0.34
S30108	0.56	0.32	0.28	0.49	0.48	0.45	0.08	0.15	0.60	0.31
S11456	0.34	0.59	0.52	0.40	0.37	0.28	0.51	0.41	0.46	0.19
S30755	0.39	0.30	0.44	0.22	0.43	0.32	0.35	0.12	0.23	0.51
S32405	0.41	0.24	0.54	0.36	0.44	0.27	0.39	0.26	0.33	0.35
S5213G5	0.87	0.77	0.64	0.77	0.80	0.63	0.32	0.34	0.64	0.31
S30349	0.52	0.56	0.67	0.59	0.48	0.43	0.45	0.43	0.55	0.32
S30655	0.52	0.34	0.77	0.58	0.54	0.43	0.58	0.54	0.59	0.45
S50741	0.86	0.58	0.40	0.72	0.85	0.54	0.06	0.07	0.55	0.32
S30440	-0.19	-0.12	0.02	-0.19	-0.11	-0.35	0.17	0.19	-0.09	-0.14
S32212	-0.12	-0.03	0.10	-0.03	-0.08	-0.29	0.16	0.16	0.10	-0.15
S32224	-0.25	-0.22	-0.08	-0.20	-0.24	-0.35	0.14	0.18	-0.13	-0.12
S32615	-0.23	-0.17	-0.09	-0.31	-0.21	-0.41	0.05	0.13	-0.16	-0.22
S5206G5	-0.17	-0.03	0.03	-0.28	-0.16	-0.36	0.18	0.17	-0.19	-0.11
S50725	-0.20	-0.23	0.01	-0.23	-0.09	-0.37	0.25	0.22	-0.10	-0.08
S32505	-0.05	-0.12	-0.12	-0.15	-0.03	-0.22	-0.01	0.00	-0.10	-0.16
S51434	-0.05	0.02	0.15	-0.06	-0.09	0.04	0.27	0.06	0.10	0.39
S51928	-0.20	-0.15	-0.02	-0.20	-0.15	-0.29	0.20	0.26	-0.12	-0.05
S30725	-0.02	-0.12	-0.07	-0.03	-0.02	0.00	0.05	0.07	-0.04	0.18
S30743	0.39	0.25	0.48	0.45	0.30	0.27	0.36	0.42	0.26	0.13
S51603	-0.01	-0.13	0.10	0.11	0.03	0.08	0.15	0.07	0.00	0.30
S52354	0.26	0.04	0.10	0.15	0.24	0.04	-0.14	0.00	0.13	0.02
S52725	0.00	-0.06	0.09	-0.01	0.02	0.00	0.23	0.19	0.08	0.15
S50252	0.30	0.24	0.22	0.31	0.34	0.17	0.06	0.10	0.16	0.27
S50804	0.10	0.12	0.21	0.01	-0.14	-0.02	0.43	0.47	0.17	0.17

Participant	V2-Apex: PG9	PG16	PGDM1400	CAP256-VRC26	CH01	PGDM1401	PGT145	MPER: 4E10	10E8	DH511.11P	PGZL1	2F5	Interface/Fusion Peptide: VRC34.01	PGT151	8ANC195	ACS202	35O22	Silent Face: SF12
S10906	0.41	0.43	0.41	0.16	0.34	0.35	0.37	-0.03	0.02	0.04	0.06	0.09	0.38	0.34	-0.17	0.01	0.24	0.29
S30402	0.01	0.06	0.04	-0.31	0.06	0.09	0.20	0.16	0.11	0.06	0.03	0.34	0.13	0.17	0.26	0.13	0.22	0.29
S31522	-0.02	0.02	-0.03	-0.32	-0.02	0.02	0.15	0.32	0.24	0.23	0.21	0.39	0.11	0.06	0.21	0.02	0.33	0.22
S50216	0.12	0.16	0.24	0.06	0.32	0.30	0.21	0.39	0.43	0.48	0.30	0.19	0.15	0.27	0.09	0.31	0.14	0.15
S50408	0.05	0.03	0.09	-0.04	0.14	0.13	0.23	0.06	0.24	0.05	0.08	0.04	0.09	0.16	0.29	0.16	0.20	0.35
S50514	0.02	-0.05	0.08	-0.13	0.07	0.08	0.20	0.08	0.17	0.18	0.08	0.05	0.12	0.15	0.05	0.17	0.04	0.16
S50616	0.16	0.15	0.12	-0.15	0.21	0.05	0.07	0.29	0.33	0.27	0.19	0.41	0.15	-0.04	0.24	-0.24	0.24	0.15
S50901	0.19	0.19	0.21	-0.07	0.22	0.13	0.20	0.39	0.36	0.32	0.21	0.50	0.21	0.06	0.00	-0.03	0.26	0.33
S52026	0.00	0.04	0.05	0.00	0.06	0.02	0.16	0.27	0.30	0.21	0.17	-0.11	0.17	0.51	0.02	0.38	0.06	0.27
S52624	0.02	0.05	0.07	-0.27	-0.01	0.13	0.12	0.19	0.17	0.12	0.10	0.36	0.04	0.16	0.13	0.15	0.13	0.32
S30108	-0.15	-0.12	-0.08	-0.24	-0.12	-0.16	-0.01	0.37	0.36	0.42	0.36	0.30	-0.04	0.11	0.00	0.09	0.27	0.07
S11456	0.16	0.21	0.24	0.17	0.14	0.22	0.08	0.13	0.09	0.13	0.17	0.09	0.21	0.43	0.04	0.12	0.14	0.14
S30755	0.12	0.03	0.16	0.02	0.10	0.19	0.19	-0.03	0.08	0.02	-0.11	0.01	0.08	0.29	-0.03	0.24	0.07	0.44
S32405	-0.04	-0.01	0.12	-0.02	0.11	0.11	0.17	0.41	0.44	0.40	0.30	0.27	0.08	0.39	0.11	0.32	0.16	0.32
S5213G5	-0.03	-0.05	0.01	0.06	0.02	-0.13	-0.02	0.04	0.02	0.05	0.07	-0.16	0.18	0.40	-0.17	0.23	0.11	0.04
S30349	0.13	0.14	0.18	-0.20	0.12	0.10	0.29	0.06	0.04	0.02	0.05	0.03	0.14	0.45	0.08	0.21	0.10	0.21
S30655	-0.05	-0.05	0.09	-0.03	0.04	0.09	0.12	0.27	0.29	0.30	0.35	0.13	-0.01	0.31	0.15	0.08	-0.01	0.09
S50741	-0.02	-0.04	0.00	0.05	0.07	-0.10	0.02	0.04	0.15	0.13	0.13	-0.08	0.17	0.38	-0.27	0.19	0.07	0.07
S30440	0.85	0.86	0.81	0.54	0.75	0.67	0.48	0.01	0.17	0.18	0.16	0.16	0.40	0.31	-0.01	-0.14	-0.10	0.22
S32212	0.65	0.62	0.71	0.44	0.38	0.47	0.32	0.03	-0.02	0.07	0.05	0.14	0.18	0.25	-0.04	0.05	0.13	0.14
S32224	0.82	0.75	0.63	0.40	0.66	0.45	0.32	0.16	0.31	0.30	0.32	0.19	0.39	0.25	-0.14	-0.25	0.10	0.10
S32615	0.67	0.62	0.70	0.48	0.58	0.54	0.32	0.23	0.32	0.33	0.33	0.30	0.23	0.04	-0.04	-0.20	0.03	0.22
S5206G5	0.56	0.57	0.64	0.59	0.55	0.51	0.21	0.19	0.25	0.29	0.26	0.21	0.30	0.09	0.02	-0.16	0.03	0.17
S50725	0.64	0.57	0.61	0.43	0.64	0.44	0.30	0.18	0.32	0.33	0.34	0.29	0.41	0.19	0.04	-0.30	0.13	0.08
S32505	0.45	0.40	0.43	0.53	0.52	0.26	0.18	0.42	0.43	0.41	0.44	0.25	0.27	-0.01	0.03	-0.26	0.18	0.00
S51434	0.20	0.13	0.25	-0.21	0.33	0.35	0.51	0.13	0.29	0.08	0.04	0.23	0.13	0.11	0.11	0.17	0.17	0.40
S51928	0.84	0.79	0.75	0.40	0.73	0.63	0.49	0.12	0.30	0.26	0.25	0.24	0.33	0.20	0.00	-0.22	-0.03	0.22
S30725	0.34	0.24	0.34	-0.04	0.30	0.34	0.40	0.39	0.58	0.44	0.44	0.37	-0.01	0.14	-0.02	-0.11	0.12	0.25
S30743	0.15	0.11	0.30	0.11	0.29	0.23	0.23	0.41	0.43	0.39	0.48	0.20	0.03	0.17	0.18	-0.02	0.18	0.22
S51603	0.07	0.09	0.14	-0.18	0.24	0.18	0.25	0.54	0.67	0.60	0.49	0.37	0.06	0.28	0.19	-0.10	0.16	0.12
S52354	0.03	-0.02	0.00	0.05	0.19	-0.13	-0.12	0.32	0.38	0.40	0.37	0.04	-0.01	0.13	-0.17	-0.01	-0.08	-0.04
S52725	0.20	0.20	0.27	0.03	0.29	0.31	0.17	0.46	0.56	0.57	0.51	0.43	0.13	0.23	0.01	-0.01	0.18	0.25
S50252	0.31	0.22	0.28	0.18	0.23	0.22	0.37	0.06	0.28	0.12	0.12	-0.19	0.39	0.62	-0.16	0.20	0.06	0.05
S50804	0.36	0.31	0.33	-0.02	0.20	0.25	0.37	0.21	0.32	0.20	0.23	0.42	0.05	0.08	-0.02	-0.08	0.14	0.53

Table S2. bnAb-inducer Env panel characteristics, related to Figures 1 and S1.

								Neutralization sensitivity to bnAbs (50% Inhibitory concentration (IC50) µg/mL																			PNGS at key Env sites (N)			
Paticipant	Subtype	Env clone	Year of isolation	Isolation weeks post EDI[1]	GenBank Accession	Extra V1Cys	bnAb Plasma Specificity (see Table S1)	VRC01	VRC-PG04	3BNC117	N6	PGT128	PGDM12	PGDM21	PGT121	PGDM1400	VRC26.25	PG16	PGT145	CH01	DH511.11P	10E8	2F5	35O22	PGT151	SF12	N156	N160	N332	N334
S10906	G	S10906-109wk-c19	2011	109	PQ282667	C0	CD4bs	0.8159	0.2495	25.0000	0.0462	25.0000	25.0000	25.0000	25.0000	25.0000	0.0148	25.0000	25.0000	25.0000	0.0244	0.0352	0.9569	0.1517	25.0000	0.2084	N			N
S30402	B	R282_3.4_cl56	2011	176	MH714324	C2	CD4bs	25.0000	25.0000	25.0000	25.0000	25.0000	25.0000	25.0000	25.0000	25.0000	25.0000	25.0000	25.0000		2.7179	5.0668	25.0000	25.0000	0.0023	0.3656	N			N
S31522	B	S31522-147wk-c20	1995	147	PQ282670	C0	CD4bs	25.0000	25.0000	25.0000	25.0000	25.0000	0.0529	0.0244	0.0050	0.2584	0.4996	0.0454	0.0197	25.0000	0.0356	0.0655	0.1947	0.0054	0.0569	6.2846	N	N	N	
S50216	B	S50216-677wk-c68	1999	677	PQ282684	C0	CD4bs	25.0000	25.0000	24.1761	25.0000	0.0244	0.0760	2.5829	0.0244	25.0000	25.0000	25.0000	25.0000	25.0000	0.6309	0.2193	1.5806	25.0000	0.0276	0.0340	N		N	
S50408	B	S50408-1083wk-c40	2003	1083	PQ282691	C0	CD4bs	25.0000	25.0000	0.1619	0.0426	25.0000	0.7507	25.0000	25.0000	0.9176	25.0000	0.5730	0.0855	7.6000	0.0244	0.0376	25.0000	25.0000	25.0000	0.0257	N	N	N	
S50514	B	S50514-891wk-c4	2001	891	PQ282692	C0	CD4bs	17.7573	25.0000	25.0000	25.0000	25.0000	25.0000	14.3757	0.9378	1.9215	25.0000	5.8198	0.1075	16.5108	0.0431	0.2048	25.0000	25.0000	25.0000	7.1989	N	N	N	
S50616	B	S50616-1071wk-c57	2011	1071	PQ282693	C0	CD4bs	25.0000	25.0000	25.0000	25.0000	0.0244	25.0000	25.0000	25.0000	25.0000	25.0000	25.0000	25.0000	10.9966	3.5333	11.8949	24.2347	25.0000	0.2730	0.2251	N	N	N	
S50901	B	S50901-909wk-c2	2002	909	PQ282697	C0	CD4bs	25.0000	25.0000	21.4372	1.1646	0.1342	1.5646	24.6354	0.4385	0.3222	25.0000	0.2493	0.0389	6.6982	0.8216	0.2568	4.6690	25.0000	13.6943	0.0926	N	N	N	
S52026	B	S52026-432wk-c83	1996	432	PQ282687	C0	CD4bs	4.2951	0.4207	0.2308	0.0660	25.0000	25.0000	25.0000	25.0000	0.8330	25.0000	1.7733	0.0876	1.0180	0.6301	0.9798	3.0835	25.0000	7.8821	1.4528	N	N	N	
S52624	B	S52624-272wk-c6	2001	272	PQ282689	C0	CD4bs	25.0000	0.0244	25.0000	0.0882	25.0000	25.0000	25.0000	25.0000	12.5861	25.0000	1.2233	25.0000	0.1775	0.0465	0.0733	0.8885	0.0998	3.7880	0.4275	N	N		N
S30108	B	S30108-217wk-c42	2001	217	PQ282669	C1	V3-Gly	23.6386	22.8348	0.3579	0.2915	3.5307	25.0000	25.0000	25.0000	9.1253	25.0000	25.0000	1.0363	25.0000	0.1524	0.4451	25.0000	25.0000	25.0000	0.0780	N	N		
S11456	A	S11456-244wk-c77	2012	244	PQ282666	C0	V3-Gly	8.4401	25.0000	25.0000	0.0807	23.0134	25.0000	25.0000	25.0000	25.0000	25.0000	0.7093	25.0000	18.0979	3.7413	3.1500	25.0000	0.0357	0.0160	0.0485	N	N		N
S30755	B	S30755-540wk-c48	2002	540	PQ282679	C0	V3-Gly	24.8538	25.0000	25.0000	3.4341	25.0000	25.0000	25.0000	25.0000	0.0579	25.0000	0.2860	0.0112	25.0000	0.8724	0.5881	3.7727	4.4843	25.0000	25.0000	N	N	N	
S32405	B	S32405-230wk-c34	2012	230	PQ282673	C0	V3-Gly	1.4319	4.2492	0.8073	0.8078	25.0000	25.0000	25.0000	25.0000	25.0000	25.0000	25.0000	8.8867	25.0000	0.1057	0.3672	1.8674	25.0000	25.0000	25.0000	N	N	N	
S5213G5	B	S5213G5-230wk-c27	2003	230	PQ282683	C0	V3-Gly	3.5508	1.9842	25.0000	0.0868	0.0244	0.1867	0.0524	0.0207	25.0000	25.0000	25.0000	25.0000	25.0000	0.0227	0.1947	1.4656	25.0000	3.1337	0.2690	N		N	
S30349	B	T282_3.8_cl82	2012	214	MH714327	C2	V3-Gly	25.0000	25.0000	25.0000	0.7185	0.0244	25.0000	25.0000	25.0000	12.6392	25.0000	3.3535	0.0326	1.7091	0.1599	0.0511	25.0000	0.2883	25.0000	1.5433	N	N	N	
S30655	B	S30655-204wk-c26	2011	204	PQ282668	C2	V3-Gly	2.8837	1.4155	0.1858	0.0788	25.0000	25.0000	25.0000	25.0000	25.0000	25.0000	25.0000	25.0000	25.0000	0.0244	0.2257	1.6201	0.0438	25.0000	0.0244	N		N	
S50741	B	S50741-847wk-c12	2006	847	PQ282695	C0	V3-Gly	3.7443	5.7849	0.2287	0.1194	25.0000	25.0000	25.0000	25.0000	25.0000	25.0000	25.0000	1.9624	25.0000	0.1702	0.1608	1.9824	13.4468	0.0141	0.1034	N	N		
S30440	A	S30440-173wk-c66	2010	173	PQ282676	C0	V2-Apex	0.2780	0.0244	0.0244	0.0244	25.0000	25.0000	25.0000	25.0000	25.0000	25.0000	25.0000	25.0000	25.0000	0.1965	4.3480	8.8286	25.0000	0.0136	0.1748	N			N
S32212	G	S32212-164wk-c59	2010	164	PQ282671	C0	V2-Apex	2.6741	0.9774	0.0944	0.4431	0.0244	25.0000	25.0000	25.0000	25.0000	25.0000	25.0000	25.0000	25.0000	0.4519	1.7645	6.9998	0.0015	0.2545	0.0520	N			N
S32224	A	S32224-174wk-c33	2009	174	PQ282672	C0	V2-Apex	25.0000	25.0000	25.0000	20.3544	25.0000	25.0000	25.0000	25.0000	0.0244	25.0000	25.0000	2.5408	25.0000	1.1711	0.6699	9.0336	25.0000	0.0169	25.0000	N	N		N
S32615	AE	S32615-178wk-c33	2008	178	PQ282675	C0	V2-Apex	6.4117	0.7241	25.0000	1.2167	0.0448	25.0000	25.0000	25.0000	25.0000	25.0000	25.0000	25.0000	25.0000	0.0285	0.5299	25.0000	0.0088	25.0000	25.0000	N			N
S5206G5	D	S5206G5-581wk-c51	2008	581	PQ282686	C2	V2-Apex	1.6076	1.5572	0.2083	0.1244	25.0000	25.0000	25.0000	25.0000	25.0000	25.0000	25.0000	25.0000	25.0000	0.1818	0.6273	4.9869	0.4615	25.0000	0.3145	N	N		
S50725	AG	S50725-295wk-c60	2011	295	PQ282694	C0	V2-Apex	25.0000	25.0000	25.0000	0.1059	25.0000	25.0000	25.0000	25.0000	0.0244	0.0058	25.0000	25.0000	10.3990	0.0707	1.3918	24.7923	25.0000	0.0453	25.0000	N	N		N
S32505	D	S32505-230wk-c18	2001	230	PQ282674	C0	V2-Apex	25.0000	25.0000	25.0000	0.4728	25.0000	25.0000	0.8285	6.7880	25.0000	25.0000	25.0000	25.0000	25.0000	0.1232	0.1140	2.0873	25.0000	25.0000	0.2526	N	N	N	
S51434	B	S51434-865wk-c3	2003	865	PQ282680	C0	V2-Apex	25.0000	25.0000	6.0020	3.3246	25.0000	25.0000	25.0000	25.0000	25.0000	25.0000	25.0000	12.5700	25.0000	2.2954	1.9772	11.3862	25.0000	25.0000	0.0244	N	N		
S51928	A	S51928-278wk-c26	1998	278	PQ282682	C0	V2-Apex	0.0244	0.1016	0.0722	0.0266	0.0244	0.0887	0.0777	0.0880	25.0000	21.2220	25.0000	4.2746	25.0000	0.0424	25.0000	5.8157	3.9850	13.2566	25.0000	N	N	N	
S30725	B	S30725-597wk-c70	2007	597	PQ282677	C0	MPER	2.1537	0.4085	0.0817	0.0929	0.0244	0.3351	25.0000	0.0169	25.0000	25.0000	25.0000	20.5443	25.0000	0.4157	1.6537	11.5026	25.0000	0.0023	0.1356	N	N	N	
S30743	CPX	S30743-346wk-c88	2001	346	PQ282678	C2	MPER	0.5863	0.1718	0.1150	0.0824	0.1214	25.0000	25.0000	25.0000	25.0000	25.0000	25.0000	13.8551	25.0000	6.8092	6.5186	25.0000	23.2643	0.0132	1.3156	N		N	
S51603	B	S51603-618wk-c58	1995	618	PQ282681	C0	MPER	23.0811	7.1167	0.8410	1.4335	10.8691	25.0000	25.0000	0.1926	25.0000	25.0000	25.0000	12.9881	25.0000	0.7993	0.6766	5.2655	25.0000	25.0000	0.0482	N	N	N	
S52354	C	S52354-263wk-c60	2006	263	PQ282688	C0	MPER	25.0000	25.0000	25.0000	25.0000	25.0000	25.0000	25.0000	25.0000	2.8656	25.0000	0.1106	1.8884	15.2191	0.1808	1.1696	25.0000	0.0101	3.7212	25.0000	N	N		
S52725	B	S52725-275wk-c19	2007	275	PQ282690	C0	MPER	12.8638	25.0000	7.6434	1.3847	0.0985	25.0000	25.0000	25.0000	25.0000	25.0000	25.0000	2.9051	25.0000	1.3474	0.6835	25.0000	25.0000	25.0000	0.0407	N	N	N	
S50252	B	S50252-603wk-c11	2001	603	PQ282685	C0	IF_FP	16.1011	2.9121	0.6029	0.8250	25.0000	25.0000	25.0000	25.0000	25.0000	25.0000	25.0000	25.0000	25.0000	0.0837	0.0738	1.3068	25.0000	25.0000	0.2331	N	N		
S50804	B	S50804-770wk-c17	2000	770	PQ282696	C0	SF	1.7647	6.2387	0.4955	0.3158	25.0000	25.0000	25.0000	25.0000	25.0000	25.0000	25.0000	25.0000	25.0000	0.2919	0.1817	25.0000	0.0007	25.0000	0.4703	N			
		**Average Isolation year**	**2005**																											

Legend
1 EDI: estimated date of infection
2 PNGS: Predicted N glycosylation site

	VRC01	VRC-PG04	3BNC117	N6	PGT128	PGDM12	PGDM21	PGT121	PGDM1400	VRC26.25	PG16	PGT145	CH01	DH511.11P	10E8	2F5	35O22	PGT151	SF12
Median IC50	16.93	14.98	14.54	0.46	25.00	25.00	25.00	25.00	25.00	25.00	25.00	17.20	25.00	0.18	0.56	7.91	25.00	13.48	0.26
Q1	2.73	1.09	0.23	0.09	0.10	25.00	25.00	11.34	4.43	25.00	3.97	1.91	16.51	0.05	0.18	2.01	0.19	0.05	0.08
Q3	25.00	25.00	25.00	2.85	25.00	25.00	25.00	25.00	25.00	25.00	25.00	25.00	25.00	0.82	1.59	25.00	25.00	25.00	5.10
IQR	22.27	23.91	24.77	2.76	24.90	0.00	0.00	13.66	20.57	0.00	21.03	23.09	8.49	0.76	1.40	22.99	24.81	24.95	5.02

Resistant (IC50 >1 µg/mL)	30	25	19	13	23	28	30	26	27	31	28	27	32	7	11	31	23	22	12
Neutralized (IC50 <1 µg/mL)	4	9	15	21	11	6	4	8	7	3	6	7	1	27	23	3	11	12	22

	Number of Envs with IC50 > 1 µg/mL																		
**bnAb inducer fingerprint**	VRC01	VRC-PG04	3BNC117	N6	PGT128	PGDM12	PGDM21	PGT121	PGDM1400	VRC26.25	PG16	PGT145	CH01	DH511.11P	10E8	2F5	35O22	PGT151	SF12
CD4bs	9	7	8	6	7	7	9	6	6	8	7	5	8	2	2	7	7	6	3
V3-Glycan	8	8	4	1	6	7	7	7	7	8	6	6	8	1	1	8	5	6	3
V2-Apex	7	5	5	3	6	8	7	8	7	8	9	9	9	2	5	9	6	5	4
MPER	4	3	2	3	2	4	5	3	5	5	4	5	5	2	3	5	4	3	2
Interface/Fusion Peptide	1	1	0	0	1	1	1	1	1	1	1	1	1	0	0	1	1	1	0
Silent Face	1	1	0	0	1	1	1	1	1	1	1	1	1	0	0	1	0	1	0

Table S3. Reference bnAbs and nAbs, related to Figures 1, 4, S1, S2 and S3

bnAb	Epitope	Reference/PubMed ID	Spearman fingerprinting (N = 43)	Neutralization of bnAb inducer Envs (N = 19)	C^1/C^2 Env neutralization (N = 14)	C^1/C^2 trimer BAMA (N = 29)
PGDM1400	V2-apex	Sok 2014 PNAS/25422458	x	x	x	x
PGDM1401	V2-apex	Sok 2014 PNAS/25422458	x			
CAP256-VRC26.25	V2-apex	Doria-Rose 2015 J Virol/26468542	x	x	x	x
PG9	V2-apex	Walker 2009 Science/19729618	x			x
PG16	V2-apex	Walker 2009 Science/19729618	x	x	x	x
PGT145	V2-apex	Walker 2011 Nature/21849977	x	x		x
CH01	V2-apex	Bonsignori 2011 J Virol/21795340	x	x		
PGT128	V3-glycan	Walker 2011 Nature/21849977	x	x	x	x
PGDM12	V3-glycan	Sok 2016 Immunity/27438765	x	x	x	x
PGDM21	V3-glycan	Sok 2016 Immunity/27438765	x	x	x	x
PGT121	V3-glycan	Walker 2011 Nature/21849977	x	x		x
PGT125	V3-glycan	Walker 2011 Nature/21849977	x			x
PGT130	V3-glycan	Walker 2011 Nature/21849977	x			x
PGT135	V3-glycan	Walker 2011 Nature/21849977	x			x
2G12	V3-glycan	Trkola 1996 J Virol/8551569	x			x
10_1074	V3-glycan	Mouquet 2012 PNAS/23115339	x			
VRC41.01	V3-glycan	Alam 2017 Sci Transl Med/28298421	x			
VRC01	CD4bs	Wu 2010 Science/20616233	x	x	x	x
VRC-PG04	CD4bs	Wu2011 Science/21835983	x	x	x	
3BNC117	CD4bs	Klein 2012 J Exp Med/22826297	x	x	x	x
N6	CD4bs	Huang 2016 Immunity/27851912	x	x	x	x
1NC9	CD4bs	Scheid 2011 Science/21764753	x			x
b12	CD4bs	Burton 1991 PNAS/1719545	x			x
1-18	CD4bs	Schommers 2020 Cell/32004464	x			x
CH235.12	CD4bs	Bonsignori 2016 Cell/26949186	x			x
NIH45-46	CD4bs	Scheid 2011 Science/21764753	x			
N49P7	CD4bs	Sajadi 2018 Cell/29731169	x			
VRC.CH31	CD4bs	Bonsignori 2012 J Virol/22301150	x			
BG24	CD4bs	Barnes 2022 Sci Adv/35960796	x			
8ANC134	CD4bs	Scheid 2011 Science/21764753	x			
HJ16	CD4bs	Corti 2010 PLoS One/20098712	x			
IOMA	CD4bs	Gristick 2016 Nat Struct Mol Biol/27617431	x			
SF12	Silent Face	Schoofs 2019 Immunity/31126879	x	x	x	x
35O22	Interface	Huang 2014 Nature/25186731	x	x	x	x
PGT151	Interface	Falkowska 2014 Immunity/24768347	x	x		x
ASC202	Interface	van Schooten 2022 PLoS Pathog/36395347	x			x
8ANC195	Interface	Scheid 2011 Science/21764753	x			
VRC34.01	Fusion Peptide	Kong 2016 Science/27174988	x			x
DH511.11P	MPER	Williams 2017 Sci Immunol/28783671	x	x	x	
10E8	MPER	Huang 2012 Nature/23151583	x	x		
2F5	MPER	Muster 1993 J Virol/7514684	x	x	x	
4E10	MPER	Buchacher 1992 Vaccines	x			
PGZL1	MPER	Zhang 2019 Nat Comm/31772165	x			
CAP256-VRC26.09	V2-apex	Doria-Rose 2014 Nature/24590074				x
48d	CD4i	Thali 1993 J Virol/7685405				x
17b	CD4i	Thali 1993 J Virol/7685405				x
b6	CD4bs	del Real 1999 Mol Immunol/10593511				x

Table S4. Multi-clade virus panels, related to Figures 1 and S1

Subtype	Tier	Env	Genbank accession	Year of isolation	Infection stage	19-virus panel	41-virus panel	PNGS at key Env sites (N)			
								N156	N160	N332	N334
A	2	BG505_W6M_ENV_A5_T332N	DQ208456	1993		x	x	N	N		
A	1B	Q23_17	AF004885	1994	chronic	x	x	N	N	N	
A	2	KER2018.11	AY736810	2000	chronic	x	x	N	N		N
A		BF520_c2_A	KX168094	1994		x		N	N	N	
A	2	MG505_W0M_ENV_A2 A1	DQ208449				x	N	N		
A	2	Q769_h5	AF407159	1996	<3mo		x	N	N		
A	2	Q842_d12_PNS70d A1	AF407160	1994	<3mo		x	N	N		N
B	2	NAB5pre_cl_1	EU023923	2003	chronic	x	x	N	N	N	
B	2	TRO clone 11	AY835445	1995	acute	x	x	N	N	N	
B	2	REJO4541 clone 67	AY835449	2001	acute	x	x	N	N		N
B	1B	BaL_26	DQ318211	1985	<12mo	x	x	N	N	N	
B	2	JR-FL	AY669728	1986	chronic	x	x	N	N	N	
B	2	JR-CSF	AY669726	1986	chronic	x	x	N	N	N	
B	2	WITO4160 clone 33	AY835451	2000	acute T/F	x	x	N	N	N	
B	2	RHPA4259 clone 7	AY835447	2000	<12mo		x	N	N	N	
B	2	QH0692 clone 42	AY835439	1994	acute		x	N		N	
B	3	PVO clone 4	AY835444	1996	acute		x	N	N	N	
B	2	NAB9pre_cl_106	EU023928	2001	chronic		x	N	N	N	
B	2	ZEnv16_1202_7	KU600818				x	N	N		
B	2	ZEnv91_0505_12	KU600815				x	N	N		
B	2	ZEnv07_0504_15	KU600814				x	N	N	N	
C	1B	HIV_25925_2	EF117273	1999	<3mo	x	x	N	N	N	
C	2	ZM106F.PB9	AY424163	1998	<3mo	x	x	N	N	N	
C	2	Du156.12	DQ411852	1999	acute	x	x	N	N	N	
C	2	HIV-001428-2	EF117266	2000	<3mo		x	N	N	N	
C	2	DU422	AY043175	1998			x	N	N	N	
C	2	53M12	AY423984	2000	<12mo		x	N	N		
C	2	ZM249M.PL1	DQ388514	2003	acute		x	N	N	N	
C	2	ZM214M.PL15	DQ388516	2003	<3mo		x	N		N	
C	2	CAP88.6m.c10	KU198436	2005	<12mo		x	N		N	
C	2	ZM233M.PB6	DQ388517	2002	<12mo		x		N	N	
C	2	CAP45_2_00_G3	DQ435682	2005	<3mo		x	N	N		N
G	2	X2088	EU885764	2006	chronic	x	x	N	N	N	
G	2	NAB13pre_cl_9	EU023937		<12mo	x	x	N	N	N	
G	2	CRF02_cl_252	EU513190	2004			x	N	N	N	
A1D	unclassified	0815_v3_c3	HM215260	2004	<12mo		x	N		N	
AE	2	C1080_c03	JN944660	1999	<12mo	x	x	N	N		N
AE	2	CNE59	HM215422	2006	<12mo	x	x	N	N		N
AE	2	CNE5	HM215415	2006	<12mo		x	N	N		N
AE	2	ZEnv92_1008_8	KU600817				x	N	N		N
AE	2	ZEnv32_0111_5	KU600816				x	N	N		N
AG	2	CRF02_cl_250	EU513189	2004	<12mo	x	x	N	N	N	

Table S5. CATNAP-derived chronic Env neutralization IC50s against reference bnAbs, related to Figures 1 and S1.

				Reference bnAb IC50 (μg/mL)																		
Env	Subtype	Accession number	Infection stage	VRC01	VRC-PG04	3BNC117	N6	PGT128	PGDM12	PGDM21	PGT121	PGDM1400	VRC26.25	PG16	PGT145	CH01	DH511.11P	10E8	2F5	35O22	PGT151	SF12
B.US.2019.1536_BL_G9_4.MZ397073	B	MZ397073	Chronic			0.03					0.04	0.02										
B.US.2015.1HB1_W4_020416_A6_S52.KY323804	B	KY323804	Chronic	0.04		0.01					25	25										
B.US.2015.1HB2_W4_103015_A11_S74.KY323867	B	KY323867	Chronic	0.11		0.02					25	25										
B.US.2015.1HB3_W4_110215_C7_S50E.KY323986	B	KY323986	Chronic	1.72		0.16					25	0.49										
B.US.2015.1HC1_W4_070516PL2_B4_S96.KY324049	B	KY324049	Chronic	0.27		0.35					25	25										
B.US.2015.1HC2_D0_012216_D1_S67.KY324102	B	KY324102	Chronic	0.28		0.66					0.01	0										
B.US.2015.1HC3_D0_012216_G3_S52.KY324191	B	KY324191	Chronic	0.46		0.13					0.06	0.02										
B.US.2015.1HD1_D0_012016_H4_S83.KY324412	B	KY324412	Chronic	0.12		0.01					0	0.2										
B.US.2015.1HD2_PREINF_051916_B4_S62.KY324507	B	KY324507	Chronic	0		0					2.9	25										
B.DE.2016.1HD4K_D0_061616PL2_A4_S53.KY324536	B	KY324536	Chronic	0.4		0.17					0	25										
B.DE.2016.1HD5K_W4_061416_A6_S32E.KY324621	B	KY324621	Chronic	0.15		0.05					25	0.31										
B.DE.2016.1HD6K_D0_061616_C10_S24.KY324663	B	KY324663	Chronic	0.66		0.23					0.02	0.13										
B.DE.2016.1HD8K_W4_070116PL3_H8_S80.KY324798	B	KY324798	Chronic	0.21		0.04					25	25										
B.DE.2016.1HD9K_D0_070816_B3_S30.KY324807	B	KY324807	Chronic	0.28		0.03					25	0.15										
C.ET..286_36.JQ362420	C	JQ362420	Chronic	0.21	0.27	0.06	0.05	0.01			0	0.01	0	0.01	0	25	0.5	0.82	25	0	0.01	
C.ET.2002.288_38.JQ362421	C	JQ362421	Chronic	1.18	0.35	0.08	0.23	0.01			0.01	0.65	25	0.21	2.61	25	0.88	0.52	25	25	25	
B.US.2001.45_01DG5.JQ609687	B	JQ609687	Chronic	0.02		0	0	0.01			0	0.03	0.01	0.02	0		0.8	0.21		25	0	
D.UG.1998.57128_VRC15.AY736829	D	AY736829	Chronic	25	25	0.48	0.8	0.06			2.04	0.01	0.07	0.14	0.12	25	0.42	0.24	25	0.11	25	
B.US.2019.6113_V14_C7_3.MZ397029	B	MZ397029	Chronic			0.02					0	0.07										
B.US..653116_NE1.MT023028	B	MT023028	Chronic															0.01	0.28			
B.US.1989.89_6.U39362	B	U39362	Chronic	0.65	0.08	0.11	0.12	0.01			0.01	25	25	25	25	25	0.99	0.21	1.53	0.46	25	
B.BR.1992.92BR020.AY669718	B	AY669718	Chronic	0.13	0.25			0.01	0.03	0.09	0.01	25		25	1.05				25			
A1.RW.1992.92RW008.AY669703	A1	AY669703	Chronic	0.04	0.01			0			0			0	0.4				10.4			
A1C.RW.1992.92RW009.AY669700	A1C	AY669700	Chronic	0.29	0.11			0.81			1.74			0.11	0.03				25			
A1CD.RW.1992.92RW024.AY669699	A1CD	AY669699	Chronic	0.33	0.31			25			25			0.08	0.07				25			
A1C.RW.1992.92RW026.AY669702	A1C	AY669702	Chronic		0.14			0.01			0.01			0.03	1.45				25			
01_AE.TH.1992.92TH021.AY669775	01_AE	AY669775	Chronic	0.5	1.29			0.01	25	25	25	25		0.05	0				25			
D.UG.1992.92UG001.AY669754	D	AY669754	Chronic	1.16	0.55			25			25			25	25				13			
D.UG.1992.92UG005.AY669758	D	AY669758	Chronic	0.58	0.44			0.02			18.3			25	25				25			
A1.UG.1992.92UG031.AY669701	A1	AY669701	Chronic	0.47	25			25			25			0.43	25				25			
D.UG.1992.92UG046.AY623599	D	AY623599	Chronic	12.7	22.4			25	25	25	25			0.08	25				25			
B.US.1992.92US715.U08451	B	U08451	Chronic	1		0.43		0.02			0.06				0.11			0.1	1.6	0.01		
C.IN.1993.93IN905.AY669742	C	AY669742	Chronic	0.1	0.33			0.01			0.01	0.29		0.25	0				25			
C.MW.1993.93MW959.AY669739	C	AY669739	Chronic	0.05	25			0.05			0.01			0.01	1.2				25			
A1.RW.1993.93RW029.AY669697	A1	AY669697	Chronic	1.57	0.54			25			25			25	25				25			
B.TH.1993.93TH305.AY669729	B	AY669729	Chronic	0.24	0.26			0.01			0.01			19.3	0.03				25			
A1.UG.1993.93UG077.AY669704	A1	AY669704	Chronic	0.1	0.08			0.01			0.02			25	25				25			
A1C.KE.1994.94KE105.AY669768	A1C	AY669768	Chronic	0.5	2.98			0.01			0.03			25	1.32				25			
A1.UG.1994.94UG103.AY669705	A1	AY669705	Chronic	0.23	0.2			0.01	2.21	25	1.51	0.02		0.05	0.19				1.92			
D.UG.1994.94UG114.AY494966	D	AY494966	Chronic	0.33	0.46			0.08			0			25	20.1				25			
08_BC.CN.1998.98CN006.AY669745	08_BC	AY669745	Chronic	0.66	1.32			0.02			0.01			25	25				25			
07_BC.CN.1998.98CN009.AY669747	07_BC	AY669747	Chronic	0.35	0.13			0.03			0.01			25	0.14				25			
C.IN.1998.98IN022.AY669748	C	AY669748	Chronic	0.65	25			0.03	0.5	0.13	0.03			0	0				25			
B.US.1986.ADA.AY426119	B	AY426119	Chronic	0.41	0.31	0.08	0.14	0.02			0.01	0.05	25	0.01	0.01	25	0.16	0.05	0.34	25	25	
B.US..BL01.AY124970	B	AY124970	Chronic	25	25	25	25	25			19.6	25	25	25	25	25	1.28	0.27	0.53	0.01	0	
B.US..BR07.AY124979	B	AY124979	Chronic	1.62	1.29	0.15	0.34	1.25			0.1	25	25	25	25	25	0.29	0.09	0.67	0	0.01	
01_AE.TH.1999.C3347_C11.JX512902	01_AE	JX512902	Chronic	0.08	25	0.03	0.01	0	25	25	25	0.01	25	0.01	0.01	25	0.09	0.02	0.12	0.01	25	0.04
C.ZA.2010.CAP378_2_00_D2_5.KF114888	C	KF114888	Chronic	25		25	0.03	25			25	25	0		25			1.71	25			
A1.TZ.2008.CH0219_E4.HM204621	A1	HM204621	Chronic	25		25	25				25	14.4	25									
01_AE.TH.1990.CM235_2.JN944662	01_AE	JN944662	Chronic	0.26		0.1					25			0.01				0.17	0.88			
01_AE.TH.1990.CM244_90TH.AY713425	01_AE	AY713425	Chronic	1.46		0.98					25			0.36				0.68	25			
01_AE.TH.1993.CMU02.AY669779	01_AE	AY669779	Chronic	0.52	1.24			25	25	25	25			25	1.43				0.6			
B.CN.2006.CNE1.HQ699949	B	HQ699949	Chronic	0.02																		
01_AE.CN.2007.CNE107.HQ699986	01_AE	HQ699986	Chronic	0.88																		
08_BC.CN.2007.CNE46.HQ699971	08_BC	HQ699971	Chronic	1.77																		
08_BC.CN.2007.CNE47.HQ699972	08_BC	HQ699972	Chronic	19.8																		
08_BC.CN.2007.CNE49.HQ699974	08_BC	HQ699974	Chronic	0.28																		
B.CN.2004.CNE64.HQ699979	B	HQ699979	Chronic	25																		
C.CN.2007.CNE65.HQ699980	C	HQ699980	Chronic	1.2																		
07_BC.CN.2007.CNE68.HQ699982	07_BC	HQ699982	Chronic	0.55																		
BC.CN.2010.CNXJ0031_3.KC807958	BC	KC807958	Chronic	25										25					25			
BC.CN.2010.CNXJ0651_9.KC807940	BC	KC807940	Chronic	10.2										0.02					25			
BC.CN.2010.CNXJ09277_8.KC807975	BC	KC807975	Chronic	25										25					25			
BC.CN.2010.CNXJ1081_10.KC807949	BC	KC807949	Chronic	25										1.06					25			
C.ZA.1999.COT6_15.DQ447266	C	DQ447266	Chronic														0.08	0.03	25			
B.US.1991.DH12.AF069140	B	AF069140	Chronic	0.23								0.04		0.32	0.01							
B.CN.2008.DRVI01_0803_11.MK054029	B	MK054029	Chronic	25							0.12							1.1				
D.CD.1983.ELI.K03454	D	K03454	Chronic	25				25	25	25	25			0.09	25							
B.US.2007.EN3C068.MN516430	B	MN516430	Chronic	2.04				0.02			25			25	2.57					25		
B.HT.1992.HT593_1.U08444	B	U08444	Chronic	0.52	0.2	0.22	0.13	25			25	0.4	25	0.14	0.06	25	0.13	0.04	0.17	25	0	
B.FR.1983.HXB2.K03455	B	K03455	Chronic	0.04	0.08	0.04	0.01	11	0.12	25	25	25	25	25	25	25	0.01	0.01	0.04	0.14	0.01	
B.DE.2017.IDC561_H05.MN871333	B	MN871333	Chronic	1.12		0.42	0.17															
B.US.1986.JRCSF.AY669726	B	AY669726	Chronic	0.21	0.12	0.03	0.1	0.01	0.18	0.22	0.04	0	25	0	0	0.29	1.99	0.28	25	0.01	0.01	
A1.KE.2000.KER2008_12.AY736809	A1	AY736809	Chronic	0.53	0.27	0.25	0.28	13.1			2.08	0	0.01	0	25	1.23	0.54	11.7	25	0	0	0.1
A1.KE.2000.KER2018_11.AY736810	A1	AY736810	Chronic	0.38	0.47	0.37	0.11	25			25	0	0	0	0	0.14	25	2.35	2.27	0	0	
A1.KE.2000.KNH1209_18.AY736813	A1	AY736813	Chronic	0.11	0.07	0.04	0.05	0.01			0	25	0.1	0.52	25	25	0.36	0.87	2.24	0.08	0	
01_AE.TH.1996.M02138.AY713424	01_AE	AY713424	Chronic	0.61	0.24	0.18	0.33	25			25	0.41	24.7	0.03	25		0.05	0.02	0.03	0.22	25	0.1
A1.KE..MB201_A1.DQ187026	A1	DQ187026	Chronic	0.19	0.05	0.52	0.09	0.01			0	0.19	25	0	2.49	0.24	0.68	0.33	0.44	25	0.01	
A1.KE..MB539_2B7.DQ187082	A1	DQ187082	Chronic	0.48	0.44	0.09	0.11	2.47			25	0	0.37	0.02	1.19	25	10.2	14.2	2.49	0.46	0	
A1.KE..MG505_W0M_A2.DQ208449	A1	DQ208449	Chronic	0.11	0.05			0.03			0.14	0.05		0.03	1.99			0.12	0.4	25	0.01	
A1.KE..MI369_A5.DQ187018	A1	DQ187018	Chronic	0.2	0.05	0.04	0.07	0.02			0.02	0	0	0.01	0.01	0.85	0.55	0.63	1.44	0	0.01	
B.CH.2001.NAB01.EU023918	B	EU023918	Chronic	1.7				0.01			0.01				1.13				1.54			
B.CH.2003.NAB5PRE_CL_1.EU023923	B	EU023923	Chronic	25	2.16			0.01			0.11	2.07		25	0.13			0.03	1.26	25	25	
B.CH.2001.NAB9PRE_CL_106.EU023928	B	EU023928	Chronic	0.84	0.07			15.8			0.07	25		18.6	25			0.01	0.21	25	25	
B.FR.1985.NL43.AY669735	B	AY669735	Chronic	0.09	0.03			25			25			0.02	0.01				2.02			
01_AE.TH.1995.NP03.KC822430	01_AE	KC822430	Chronic													25						
G.ES.2002.P0402_C2_11.EU885759	G	EU885759	Chronic	0.15	0.95	0.06	0.05	0.01	0.11	25	0.01	0.03	0	0.05	0.02	25	0.15	0.11	14.1	0.5	0	0.03
F1.ES.2007.P1655_C8_B5.FJ817371	F1	FJ817371	Chronic																25			

D.ES.2007.P1741_A2.HM068556	D	HM068556	Chronic	2.18		0.31	0.43				25	1.31	25									
D.CG.2007.P940_2_C3.HM068555	D	HM068555	Chronic	25		25	25				25	0.17	0									
A1D.KE.1996.QA790_204I_C8.FJ866126	A1D	FJ866126	Chronic	0.12				25			25			0.45	25							
C.KE.1995.QB099_391M_C8.FJ866132	C	FJ866132	Chronic	0.15				0.06			0.03			0.06	25							
D.KE.1997.QB857_110I_B3.FJ866138	D	FJ866138	Chronic	25				0.07			25			0.2	0.65							
D.KE.1999.QD435_100M_A4.FJ866139	D	FJ866139	Chronic	0.35				25			25			25	0.2							
B.US.1989.R2.AF128126	B	AF128126	Chronic	0.21	0.26									25								
01_AE.TH.1999.R3265_C6.JX512903	01_AE	JX512903	Chronic	0.35	1.68	0.08	0.06	25	25	25	25	0.01	25	0.02	0.02	1.51	25	1.91	25	25	25	0.01
B.US.1987.SG3.L02317	B	L02317	Chronic	0.03	0.06			25			0.12			25	25							
01_AE.TH.1992.TH023_6.KU562843	01_AE	KU562843	Chronic	0.88		0.05	0.02	0.43			25	0.02	25	25	0.02	25	0	0	0.01	0.09	25	
01_AE.TH.1993.TH966_8.U08456	01_AE	U08456	Chronic	0.31	0.16	0.06	0.05	0.01			25	0.01	0.01	0	0	0.05	0.13	0.03	0.18	25	25	
01_AE.TH.1993.TH976_17.U08458	01_AE	U08458	Chronic	0.16	0.02	0.03	0.06	25			25	25	0.01	25	25	25	0.44	0.25	0.13	0.47	25	0.08
C.ZA.1998.TV1_29.EU855132	C	EU855132	Chronic	25	25	25	0.74	0.1			0.1	0	0.46	0	25	0.56	0.62	0.34	25	25	0	
C.TZ.2001.TZA125_17.JQ362423	C	JQ362423	Chronic	25	25	25	0.71	0.66			25	0.05	0	0.02	0.12	25	0.47	0.32	25	25	0.07	
C.TZ.2001.TZBD_02.JQ362424	C	JQ362424	Chronic	0.08	0.07	25	0.02	25			0	0.01	0	0.02	0.04	20	2.4	1.93	25	25	0	
D.UG.1992.UG021_16.U27399	D	U27399	Chronic	0.35	25	0.4	0.04	25	25	0.21	1.87	25	25	25	25		0.02	0.06	25	25	0.01	
D.UG.1992.UG024_2.U43386	D	U43386	Chronic	0.18	0.2	0.06	0.03	25	0.05	25	25	0.5	0.13	1.18	1.5		0.11	0.04	0.04	25	25	
A1.UG.1992.UG037_8.U09127	A1	U09127	Chronic	0.08	0.05	0.02	0.04	0.02			0.06	0.07	25	0	2.83	0.37	0.21	0.12	0.31	0.03	25	
B.US.2016.VA40774.MK499378	B	MK499378	Chronic	0.47		0.05	0.15	0.03			25	25	25	25				25	12.6			
A1.BE..VLGC_A1.AY669698	A1	AY669698	Chronic	0.16	0.02			0.84			25			0.18	25				25			
BG.ES.2002.X1100_C7.EU885760	BG	EU885760	Chronic	1.3															25			
G.ES.2002.X1193_C1.EU885761	G	EU885761	Chronic	0.14	0.04	0.07	0.07	0.02	0.28	0.23	0.02	0.14	0.01	0.01	0.01	25	0.32	0.19	25	0.32	0.01	25
G.ES.2003.X1254_C3.EU885762	G	EU885762	Chronic	0.05	0.05	0.07	0.03	0.02	25	0.11	0.02	25	25	0.02	2.96	25	1.34	25	19.7	25	0.09	0.07
G.ES.2004.X1632_S2_B10.FJ817370	G	FJ817370	Chronic	0.12	0.01	25	0.06	25	25	25	25	0.01	0	0.01	0.02	2.05	0.73	0.68	25	0.56	0.13	0.03
G.ES.2006.X2088_C9.EU885764	G	EU885764	Chronic	25	25	25	0.04	25	0.07	0.03	0	25	25	25	25	25	25	25	25	25	25	25
G.ES.2007.X2160_C25.EU885765	G	EU885765	Chronic	0.84															25			
14_BG.ES.2007.X2252_C7.EU885766	14_BG	EU885766	Chronic	0.36															25			
G.ES.2006.X558_8_B12_6.GQ222684	G	GQ222684	Chronic																25			
07_BC.CN..YN148R_9.HQ326139	07_BC	HQ326139	Chronic	1.55							0.13			0.12		25		1.64	25			
B.US.1986.YU2.M93258	B	M93258	Chronic	0.09	0.09	0.03	0.03	0.06			0.07	0.3	25	0.07	0.09	25	25	1.87	10.7	0.29	11.4	0.51
B.US.2012.Z258_2012_SGA5.KX595122	B	KX595122	Chronic	25	25	25	25															
C.ZA.1997.ZA012_29.EU855133	C	EU855133	Chronic	0.27	0.12	0.07	0.03	0.03			0	2.53	2.25	1.38	0.97	25	2.29	1.39	25	25	0.06	
C.ZM.2000.ZM109_4.AY424138	C	AY424138	Chronic	0.12	0.02	0.08	0.04	25	25	25	25	0.02	0	25	0.03	25	0.3	0.21	25	25	0.46	0.24
C.ZM.1998.ZM135_10A.AY424079	C	AY424079	Chronic	0.86	25	0.06	0.14	25	25	1.29	0.89	0	25	25	25	25	0.16	0.11	25	25	25	25
C.ZM.2000.ZM53_12.AY423984	C	AY423984	Chronic	0.93	1.93	0.23	0.2	25	25	25	0	0.01	0	0.02	0.31	25	25	2.78	25	25	25	25

IC50	VRC01	VRC-PG04	3BNC117	N6	PGT128	PGDM12	PGDM21	PGT121	PGDM1400	VRC26.25	PG16	PGT145	CH01	DH511.11P	10E8	2F5	35O22	PGT151	SF12
Median	0.41	0.27	0.09	0.08	0.06	25	25	1.51	0.19	13.5	0.18	1.19	25	0.48	0.25	25	12.8	0.08	0.1
Q1	0.16	0.08	0.04	0.04	0.01	0.18	0.22	0.01	0.02	0.01	0.02	0.03	2.05	0.15	0.07	1.54	0.08	0.01	0.05
Q3	1.38	1.5	0.4	0.22	25	25	25	25	25	25	25	25	25	1.3	1.24	25	25	25	18.9
IQR	1.22	1.42	0.36	0.18	25	24.8	24.8	25	25	25	25	25	23	1.14	1.17	23.5	24.9	25	18.8
Resistant (IC50 >1 µg/mL)	34	21	10	4	31	13	14	50	24	23	33	41	30	11	14	65	22	18	4
Neutralized (IC50 <1 µg/mL)	80	46	54	42	46	8	7	49	43	21	50	36	7	29	37	18	22	24	10
Number of virus tested per bnAb in CATNAP	114	67	64	46	77	21	21	99	67	44	83	77	37	40	51	83	44	42	14

Table S6. Predicted specificity of bnAb inducer plasma shown in Figures S3C and S3D.

		Spearman correlation to reference bnAbs							
	Participant	S5206G5	S30440	S30834	S30931	S51928	S50725	S5213G5	S10437
	Subtype	D	A	C	A	A	AG	B	A
	Predicted plasma specificity (defined by MSBP as in Rusert et al. 2016)	V2-apex	V2-apex	CD4bs	V2-apex	V2-apex	V2-apex	V3-glycan	MPER
V2-apex	PG9	0.56	0.85	0.31	0.83	0.84	0.64	-0.03	0.04
	PG16	0.57	0.86	0.35	0.81	0.79	0.57	-0.05	0.10
	PGDM1400	0.64	0.81	0.41	0.77	0.75	0.61	0.01	0.18
	CAP256-VRC26.25	0.59	0.54	0.27	0.48	0.40	0.43	0.06	0.11
	CH01	0.55	0.75	0.38	0.74	0.73	0.64	0.02	0.30
	PGDM1401	0.51	0.67	0.30	0.59	0.63	0.44	-0.13	0.16
	PGT145	0.21	0.48	0.26	0.41	0.49	0.30	-0.02	-0.02
V3-glycan	10_1074	-0.17	-0.19	0.13	-0.21	-0.20	-0.20	0.87	0.23
	PGT121	-0.03	-0.12	0.25	-0.15	-0.15	-0.23	0.77	0.21
	PGT128	0.03	0.02	0.36	-0.10	-0.02	0.01	0.64	0.39
	VRC41.01	-0.28	-0.19	0.20	-0.23	-0.20	-0.23	0.77	0.19
	PGDM12	-0.16	-0.11	0.11	-0.17	-0.15	-0.09	0.80	0.21
	PGDM21	-0.36	-0.35	0.01	-0.34	-0.29	-0.37	0.63	0.07
	PGT125	0.18	0.17	0.32	0.12	0.20	0.25	0.32	0.36
	PGT130	0.17	0.19	0.23	0.06	0.26	0.22	0.34	0.24
	PGT135	-0.19	-0.09	0.11	-0.11	-0.12	-0.10	0.64	0.35
	2G12	-0.11	-0.14	0.00	-0.11	-0.05	-0.08	0.31	0.04
CD4bs	VRC01	0.07	0.13	0.25	0.14	0.07	0.25	0.25	0.22
	VRC-PG04	0.15	0.04	0.38	0.07	-0.05	0.18	0.34	0.20
	3BNC117	0.12	0.22	0.48	0.20	0.25	0.27	0.18	0.23
	NIH45-46	-0.04	-0.01	0.24	-0.06	-0.06	0.11	0.23	0.23
	N6	0.07	0.08	0.26	0.04	0.05	0.28	0.40	0.23
	1NC9	0.11	0.04	0.39	-0.01	0.04	0.11	0.17	0.04
	N49P7	0.02	0.01	0.20	-0.01	0.04	0.21	0.24	0.16
	CH235.12	0.13	0.18	0.39	0.10	0.21	0.24	0.26	-0.04
	1-18	0.05	0.05	0.29	0.03	0.04	0.26	0.43	0.39
	VRC.CH31	0.00	0.05	0.17	0.10	0.04	0.19	0.21	0.19
	BG24	0.07	0.07	0.36	0.08	0.06	0.26	0.22	0.27
	8ANC134	-0.09	-0.03	0.06	-0.07	-0.01	0.00	0.23	0.08
	HJ16	-0.08	-0.21	-0.04	-0.31	-0.23	-0.14	-0.03	-0.15
	IOMA	-0.06	-0.19	0.19	-0.07	-0.13	-0.09	0.05	0.20
	b12	-0.22	-0.40	0.10	-0.32	-0.22	-0.16	0.03	-0.16
Interface/Fusion Peptide	VRC34.01	0.30	0.40	0.29	0.38	0.33	0.41	0.18	0.15
	PGT151	0.09	0.31	0.31	0.15	0.20	0.19	0.40	0.32
	8ANC195	0.02	-0.01	-0.03	-0.05	0.00	0.04	-0.17	-0.04
	ACS202	-0.16	-0.14	0.22	-0.24	-0.22	-0.30	0.23	0.03
	35O22	0.03	-0.10	0.09	-0.06	-0.03	0.13	0.11	0.04
MPER	4E10	0.19	0.01	0.19	0.07	0.12	0.18	0.04	0.25
	10E8	0.25	0.17	0.15	0.23	0.30	0.32	0.02	0.22
	DH511.11P	0.29	0.18	0.17	0.25	0.26	0.33	0.05	0.40
	PGZL1	0.26	0.16	0.17	0.20	0.25	0.34	0.07	0.30
	2F5	0.21	0.16	-0.06	0.19	0.24	0.29	-0.16	0.22
SF	SF12	0.17	0.22	0.18	0.11	0.22	0.08	0.04	0.07

Table S7. NT50 of heterologous bnAb inducer plasma against S5206G5-42wk Env variants shown in Figure 3.

Longitudinal heterologous bnAb inducer plasma NT50

		Env S5206G5-42wk-c6.2					Env S5206G5-42wk-c3	
Participant	WPI	C^2	C^1	$C^{0(WT)}$	Participant	WPI	$C^{2(WT)}$	C^1
S30440	27	100	100	100	S30440	27	108.8	213.1
	85	115.9	105.7	186.1		85	800.8	962.8
	140	350.3	359.2	346		140	1458	3082
	199	420.8	354.7	317.7		199	1392	5634
S30834	208	208	100	100	S30834	208	100	100
	416	355.1	443.4	356.3		416	100	100
	470	1096	602.3	953.4		470	100	100
	587	259.9	253	1150		587	100	100
	621	112	281.6	266.6		621	100	100
	751	107	325	403		751	100	100
S30931	272	100	100	100	S30931	272	655	749.9
	352	100	100	100		352	406.7	515.6
	469	100	100	100		469	860.9	1586
	783	100	100	100		783	149	232.8
	843	100	100	100		843	296.1	305.1
	895	100	100	100		895	147.8	261.9
S51928	343	100	100	100	S51928	343	100	100
	441	100	100	100		441	100	100
	574	100	100	100		574	100	100
	651	100	100	100		651	100	100
	766	100	100	100		766	100	100
S50725	295	100	100	100	S50725	295	100	100
	320	100	100	100		320	100	100
	342	100	100	100		342	100	100
	368	100	100	100		368	100	100
	394	100	100	100		394	100	100
	451	100	100	149.6		451	100	100
	479	100	100	100		479	100	100
S5213G5	260	100	100	100	S5213G5	260	100	100
	314	100	100	100		314	100	100
	369	100	100	100		369	100	100
	427	100	100	100		427	100	100
S10437	15	100	100	100	S10437	15	100	100
	76	100	100	100		76	100	100
	108	100	100	100		108	100	100
	325	100	100	100		325	100	100
Mixed effects model p-value (reference is WT)		0.36481	0.28477	Ref			Ref	0.174

Table S8. S5206G5-42wk-c3 soluble Env trimers: list of stabilizing mutations, related to Figures 4 and S3.

Mutation	Annotation	Reference/PubMed ID	Comment
E47D	TD8	Guenaga 2016 J Virol/26719252	
T49E	TD8	Guenaga 2016 J Virol/26719252	
A65K	TD8	Guenaga 2016 J Virol/26719252	
E106T	TD8	Guenaga 2016 J Virol/26719252	
V154M	4mut	Chuang 2017 J Virol/28275193	
I201C	DS	Kwon 2015 Nat Strct Mol Biol/26098315	only used in DS-SOSIP version
Y300M	4mut	Chuang 2017 J Virol/28275193	
N302M	4mut	Chuang 2017 J Virol/28275193	
S320L	4mut	Chuang 2017 J Virol/28275193	
G429R	TD8	Guenaga 2016 J Virol/26719252	
K432Q	TD8	Guenaga 2016 J Virol/26719252	
A433C	DS	Kwon 2015 Nat Strct Mol Biol/26098315	only used in DS-SOSIP version
A501C	SOS	Sanders 2013 PloS Pat/24068931	
E509R	optimized furin cleavage site	Sanders 2013 PloS Pat/24068931	
K510R	optimized furin cleavage site	Sanders 2013 PloS Pat/24068931	
511(+1)R	optimized furin cleavage site	Sanders 2013 PloS Pat/24068931	
511(+2)R	optimized furin cleavage site	Sanders 2013 PloS Pat/24068931	
F519R	FP-stabilization	Guenaga 2017 Immunity/28514686	
L520R	FP-stabilization	Guenaga 2017 Immunity/28514686	
V535M	Trimer-formation	de Taeye 2015 Cell/26687358	
I559P	IP	Sanders 2013 PloS Pat/24068931	
A561P	MD39	Steichen 2016 Immunity/27617678	
L568D	MD39	Steichen 2016 Immunity/27617678	
T569G	helix-disrupting glycine (2gly)	Guenaga 2017 Immunity/28514686	
V570H	MD39	Steichen 2016 Immunity/27617678	
R585H	MD39	Steichen 2016 Immunity/27617678	
T605C	SOS	Sanders 2013 PloS Pat/24068931	
Q619L	anti-TD8	Guenaga 2016 J Virol/26719252	
D636G	helix-disrupting glycine (2gly)	Guenaga 2017 Immunity/28514686	
D648E	FP-stabilization	Guenaga 2017 Immunity/28514686	

Table S10. Primer sequences, related to STAR methods

Primer name	Sequence (5'-3')
S5206G5-42wk-c3_C146eS_SENSE	CTGATGCCAATTCAAACAGTAACGCCACTGTGGA
S5206G5-42wk-c3_C146eS_antiSENSE	TCCACAGTGGCGTTACTGTTTGAATTGGCATCAG
S5206G5-42wk-c6.2_R144C_SENSE	CTGATGCCCATTCAAACTGTAGCACCACTGTGGAG
S5206G5-42wk-c6.2_R144C_antiSENSE	CTCCACAGTGGTGCTACAGTTTGAATGGGCATCA
S5206G5-42wk-c6.2_H137C_SENSE	GCTGCACCAATGCCACTTTAAACTGTACTGATGCCCATTCAAAC
S5206G5-42wk-c6.2_H137C_antiSENSE	GTTTGAATGGGCATCAGTACAGTTTAAAGTGGCATTGGTGCAGC
S5206G5-42wk-c7.2_T149_T150insC_SENSE	CAAACTGATGCCAACACCTGCACTGTGGAGTCAAAACTG
S5206G5-42wk-c7.2_T149_T150insC_antiSENSE	CAGTTTTGACTCCACAGTGCAGGTGTTGGCATCAGT TTG
S5206G5-42wk-c6_C137S_SENSE	GCACCAATGCCAATTTAAACAGCACCAATGCCACAT
S5206G5-42wk-c6_C137S_antiSENSE	ATGTGGCATTGGTGCTGTTTAAATTGGCATTGGTGC
S5206G5-42wk-c6_C157S_SENSE	AATGAGACCAATTCAACCAGCACTGTGGAGTCAAAAC
S5206G5-42wk-c6_C157S_antiSENSE	GTTTTGACTCCACAGTGCTGGTTGAATTGGTCTCATT
S5206G5-42wk-c2_C137S_SENSE	CACCAAAGCCACTTTAAACAGCACCAATGCCGCT
S5206G5-42wk-c2_C137S_antiSENSE	AGCGGCATTGGTGCTGTTTAAAGTGGCTTTGGTG
S5206G5-42wk-c2_C150S_SENSE	GTACTACCAATTCAACCAGTAACGCCACTGTGGAG
S5206G5-42wk-c2_C150S_antiSENSE	CTCCACAGTGGCGTTACTGGTTGAATTGGTAGTAC
S5206G5-42wk-c3TRIMER_C146eS_SENSE	CGCCAACAGCAACAGCAACGCCACCGT
S5206G5-42wk-c3TRIMER_C146eS_antiSENSE	ACGGTGGCGTTGCTGTTGCTGTTGGCG
S5206G5-42wk-c3TRIMER_C137S_SENSE	GCACCAACGCCAACCTGAATAGCACAAATGCCAC
S5206G5-42wk-c3TRIMER_C137S_antiSENSE	GTGGCATTTGTGCTATTCAGGTTGGCGTTGGTCC
