# Supplementary material for: Rare twin cysteine residues in the HIV-1 envelope variable region 1 link to neutralization escape and breadth development
Source: Cell Host Microbe. Author manuscript; Available in PMC 2026 Sep 29. (PMC13621619; doi:10.1016/j.chom.2025.01.004)
Supplement: Supplementary file 2 [file NIHMS2116957-supplement-MMC2.pdf]

For the best experience, open this PDF portfolio in Acrobat X or Adobe Reader X, or later.

Get Adobe Reader Now!
