# Supplementary material for: Rare twin cysteine residues in the HIV-1 envelope variable region 1 link to neutralization escape and breadth development
Source: Cell Host Microbe. Author manuscript; Available in PMC 2026 Sep 29. (PMC13621955; doi:10.1016/j.chom.2026.05.021)
Supplement: Supplementary file 1 [file NIHMS2187495-supplement-Table_S1.pdf]

| Participant code (Rusert et al Ref. 19) | Swiss4.5K Neutralization category | Subtype | Predicted bnAb specificity of donor plasma (defined by MSBP as in Ref. 19) | Plasma NT50 on 41-virus panel | | | | | | | | | | | | | | | | | |
|---|---|---|---|---|---|---|---|---|---|---|---|---|---|---|---|---|---|---|---|---|---|
| | | | | ZEnv16_1202_7 | RHPA4259 clone 7 | ZEnv91_0505_12 | NAB13pre_cl_9 | BaL_26 | QH0692 clone 42 | MG505_W0M_ENV_A2 A1 | X2088 | REJO4541 clone 67 | Q769_h5 | C1080_c03 | ZEnv07_0504_15 | NAB5pre_cl_1 | CRF02_cl_250 | HIV-001428-2 | TRO clone 11 | DU422 | JR-CSF |
| S10906 | elite | G | CD4bs | 2893 | 291 | 529 | 14207 | 2071 | 172 | 1815 | 383 | 894 | 1839 | 3786 | 464 | 103 | 5181 | 3786 | 2141 | 761 | 1829 |
| S30402 | elite | B | CD4bs | 1479 | 1432 | 148 | 1118 | 6105 | 273 | 848 | 40 | 821 | 710 | 227 | 122 | 145 | 159 | 3953 | 382 | 40 | 566 |
| S31522 | elite | B | CD4bs | 2079 | 1568 | 340 | 4204 | 7825 | 723 | 877 | 100 | 2180 | 782 | 962 | 40 | 508 | 217 | 1599 | 1883 | 150 | 891 |
| S50216 | elite | B | CD4bs | 40 | 859 | 409 | 1331 | 1514 | 441 | 617 | 40 | 386 | 40 | 1964 | 423 | 40 | 342 | 311 | 665 | 103 | 3356 |
| S50408 | broad | B | CD4bs | 1944 | 714 | 764 | 40 | 1340 | 40 | 376 | 488 | 1198 | 235 | 840 | 957 | 465 | 381 | 281 | 931 | 381 | 569 |
| S50514 | elite | B | CD4bs | 701 | 1305 | 713 | 1509 | 3710 | 533 | 134 | 40 | 983 | 1153 | 605 | 387 | 194 | 904 | 1454 | 2420 | 40 | 454 |
| S50616 | elite | B | CD4bs | 1241 | 685 | 879 | 2751 | 3596 | 1264 | 794 | 40 | 1743 | 1459 | 3726 | 527 | 148 | 577 | 2231 | 1296 | 661 | 703 |
| S50901 | elite | B | CD4bs | 902 | 387 | 285 | 2664 | 15465 | 2494 | 828 | 40 | 1259 | 486 | 2180 | 152 | 40 | 212 | 3158 | 395 | 156 | 698 |
| S52026 | cross | B | CD4bs | 1026 | 1375 | 107 | 40 | 3214 | 174 | 741 | 105 | 1717 | 173 | 301 | 868 | 106 | 434 | 2354 | 1183 | 422 | 4261 |
| S52624 | elite | B | CD4bs | 1393 | 1303 | 162 | 6637 | 8297 | 802 | 668 | 40 | 549 | 1084 | 444 | 439 | 205 | 679 | 2194 | 1217 | 40 | 3627 |
| S30108 | elite | B | V3-Gly | 111 | 373 | 102 | 5308 | 7809 | 812 | 138 | 1879 | 1181 | 40 | 1218 | 40 | 265 | 487 | 372 | 2595 | 342 | 1222 |
| S11456 | broad | A | V3-Gly | 698 | 555 | 40 | 1368 | 9677 | 297 | 7778 | 74 | 955 | 40 | 329 | 40 | 630 | 6391 | 2166 | 2078 | 1018 | 1855 |
| S30755 | broad | B | V3-Gly | 2374 | 1474 | 187 | 40 | 8959 | 790 | 40 | 254 | 586 | 434 | 253 | 494 | 183 | 596 | 162 | 3844 | 40 | 2557 |
| S32405 | elite | B | V3-Gly | 232 | 2012 | 136 | 188 | 3080 | 652 | 451 | 40 | 552 | 40 | 269 | 439 | 40 | 845 | 179 | 1115 | 40 | 1968 |
| S5213G5 | elite | B | V3-Gly | 40 | 528 | 40 | 40 | 3278 | 140 | 106 | 2023 | 280 | 40 | 40 | 40 | 226 | 4251 | 523 | 2985 | 742 | 4705 |
| S30349 | elite | B | V3-Gly | 1025 | 2943 | 382 | 527 | 2422 | 231 | 572 | 40 | 1510 | 1772 | 507 | 265 | 2434 | 3711 | 5668 | 1691 | 630 | 6989 |
| S30655 | broad | B | V3-Gly | 178 | 2806 | 40 | 742 | 4254 | 1272 | 236 | 94 | 372 | 40 | 982 | 224 | 8563 | 25556 | 700 | 6837 | 681 | 6183 |
| S50741 | broad | B | V3-Gly | 40 | 1034 | 40 | 131 | 4286 | 381 | 116 | 1420 | 475 | 40 | 90 | 1220 | 399 | 6518 | 1043 | 2900 | 829 | 1362 |
| S30440 | elite | A | V2-Apex | 929 | 472 | 40 | 885 | 511 | 113 | 2895 | 40 | 635 | 1823 | 8547 | 400 | 40 | 13396 | 2433 | 195 | 40 | 1002 |
| S32212 | elite | G | V2-Apex | 102 | 40 | 40 | 8905 | 133 | 40 | 970 | 40 | 134 | 3454 | 2399 | 40 | 40 | 8961 | 742 | 40 | 40 | 1238 |
| S32224 | elite | A | V2-Apex | 588 | 327 | 552 | 4929 | 5040 | 152 | 5739 | 272 | 588 | 2988 | 144035 | 497 | 259 | 20262 | 6270 | 456 | 937 | 18189 |
| S32615 | elite | AE | V2-Apex | 334 | 91 | 40 | 613 | 381 | 129 | 202 | 40 | 250 | 120 | 14799 | 40 | 40 | 1270 | 444 | 120 | 40 | 246 |
| S5206G5 | elite | D | V2-Apex | 1064 | 146 | 40 | 340 | 1781 | 4350 | 1906 | 40 | 656 | 40 | 189008 | 40 | 174 | 39189 | 1808 | 851 | 40 | 259 |
| S50725 | elite | AG | V2-Apex | 841 | 507 | 216 | 14167 | 3312 | 322 | 6958 | 40 | 457 | 898 | 4545009 | 169 | 215 | 105847 | 4444 | 912 | 40 | 746 |
| S32505 | elite | D | V2-Apex | 117 | 55 | 113 | 1022 | 907 | 625 | 801 | 132 | 356 | 40 | 1586 | 40 | 40 | | 758 | 603 | 205 | 175 |
| S51434 | broad | B | V2-Apex | 6146 | 5644 | 5710 | 1011 | 26278 | 762 | 1466 | 396 | 9281 | 671 | 2444 | 1704 | 2844 | 839 | 2721 | 2458 | 602 | 9150 |
| S51928 | elite | A | V2-Apex | 663 | 114 | 40 | 205 | 469 | 102 | 516 | 40 | 123 | 272 | 87571 | 40 | 40 | 1796 | 858 | 109 | 40 | 5965 |
| S30725 | elite | B | MPER | 3198 | 371 | 40 | 734 | 1769 | 929 | 132 | 40 | 1545 | 40 | 1148 | 283 | 311 | 810 | 40 | 456 | 162 | 2545 |
| S30743 | cross | CPX | MPER | 86 | 230 | 40 | 40 | 1680 | 104 | 167 | 41 | 223 | 55 | 3123 | 133 | 511 | 10245 | 730 | 1181 | 689 | 169 |
| S51603 | cross | B | MPER | 702 | 928 | 40 | 981 | 2250 | 1423 | 468 | 40 | 2361 | 125 | 5639 | 1568 | 283 | 860 | 40 | 1255 | 154 | 975 |
| S52354 | cross | C | MPER | 75 | 510 | 418 | 320 | 861 | 465 | 116 | 40 | 181 | 167 | 188 | 171 | 419 | 628 | 367 | 402 | 280 | 452 |
| S52725 | elite | B | MPER | 922 | 748 | 225 | 4175 | 3431 | 532 | 797 | 539 | 728 | 40 | 6913 | 605 | 283 | 729 | 219 | 1571 | 542 | 903 |
| S50252 | elite | B | IF_FP | 4350 | 674 | 40 | 233 | 11719 | 151 | 619 | 316 | 1784 | 453 | 629 | 872 | 475 | 1167 | 171 | 621 | 499 | 2576 |
| S50804 | broad | B | SF | 1470 | 310 | 251 | 596 | 1494 | 111 | 706 | 122 | 1400 | 399 | 2381 | 239 | 411 | 4185 | 1331 | 675 | 1264 | 1693 |

| Participant code | HIV_25925_2 | ZM106F.PB9 | 53M12 | PVO clone 4 | KER2018.11 | ZM214M.PL15 | 0815_v3_c3 | ZEnv32_0111_5 | CNE5 | CRF02_cl_252 | ZM249M.PL1 | NAB9pre_cl_106 | CAP88.6m.c10 | JR-FL | ZM233M.PB6 | CAP45_2_00_G3 | ZEnv92_1008_8 | Q842_d12_PNS70d A1 | Du156.12 | BG505_W6M_ENV_A5_T332N | WITO4160 clone 33 | CNE59 | Q23_17 | Breadth | Potency (Geometric Mean NT50) |
|---|---|---|---|---|---|---|---|---|---|---|---|---|---|---|---|---|---|---|---|---|---|---|---|---|---|
| S10906 | 2125 | 2353 | 208 | 458 | 3851 | 40 | 5443 | 119 | 137 | 194 | 1155 | 242 | 112 | 3229 | 40 | 875 | 919 | 9635 | 1744 | 3330 | 2126 | 105 | 3704 | 95% | 1030.38 |
| S30402 | 40 | 179 | 135 | 479 | 40 | 40 | 956 | 272 | 176 | 40 | 121 | 84 | 40 | 2373 | 40 | 40 | 40 | 1959 | 1136 | 788 | 254 | 205 | 724 | 76% | 480.286 |
| S31522 | 483 | 104 | 505 | 1014 | 40 | 40 | 1415 | 2601 | 40 | 40 | 1052 | 148 | 40 | 4625 | 40 | 40 | 625 | 5278 | 1268 | 1119 | 919 | 798 | 1220 | 80% | 912.13 |
| S50216 | 543 | 332 | 164 | 1769 | 355 | 40 | 186 | 118 | 40 | 40 | 422 | 40 | 464 | 2324 | 279 | 960 | 1311 | 450 | 1660 | 1258 | 290 | 4031 | 731 | 80% | 609.21 |
| S50408 | 594 | 427 | 163 | 812 | 128 | 436 | 40 | 490 | 149 | 236 | 40 | 130 | 40 | 889 | 1036 | 226 | 747 | 578 | 724 | 1099 | 201 | | 3313 | 88% | 520.454 |
| S50514 | 1422 | 1759 | 40 | 1141 | 40 | 40 | 176 | 40 | 124 | 327 | 329 | 851 | 117 | 2099 | 1843 | 40 | 154 | 1244 | 1439 | 2144 | 786 | 1169 | 429 | 83% | 715.892 |
| S50616 | 40 | 655 | 500 | 328 | 389 | 1403 | 1175 | 5630 | 589 | 40 | 766 | 359 | 40 | 6322 | 128 | 1950 | 1133 | 4182 | 6473 | 2601 | 1238 | 17037 | 2059 | 90% | 1217.68 |
| S50901 | 409 | 606 | 537 | 191 | 135 | 356 | 602 | 315 | 40 | 40 | 179 | 1304 | 188 | 1941 | 139 | 1901 | 1019 | 1276 | 957 | 780 | 549 | 844 | 3750 | 90% | 672.999 |
| S52026 | 2816 | 696 | 386 | 742 | 40 | 40 | 257 | 195 | 40 | 348 | 590 | 839 | 4821 | 4990 | 654 | 303 | 615 | 914 | 37144 | 1805 | 40 | 341 | 1350 | 88% | 758.075 |
| S52624 | 40 | 338 | 662 | 881 | 339 | 40 | 2069 | 694 | 40 | 40 | 198 | 340 | 40 | 8077 | 40 | 40 | 473 | 2559 | 1348 | 1642 | 40 | 711 | 1226 | 76% | 943.614 |
| S30108 | 1708 | 623 | 40 | 223 | 40 | 40 | 303 | 308 | 40 | 391 | 461 | 462 | 102 | 2216 | 109 | 40 | 40 | 40 | 793 | 744 | 104 | 888 | 1789 | 78% | 565.873 |
| S11456 | 161 | 1300 | 1948 | 357 | 180 | 40 | 851 | 40 | 100 | 2627 | 359 | 40 | 202 | 2254 | | 131 | 251 | 4832 | 4031 | 1183 | 40 | 573 | 40859 | 83% | 946.104 |
| S30755 | 845 | 531 | 285 | 3148 | | 40 | 117 | 40 | 251 | 276 | 731 | 900 | 40 | 4413 | 1463 | 40 | 1325 | 3780 | 3021 | 9705 | 447 | 149 | 3210 | 83% | 836.98 |
| S32405 | 1373 | 506 | 40 | 455 | 40 | 162 | 104 | 199 | 40 | 194 | 618 | 671 | 395 | 1021 | 143 | 40 | 1257 | 238 | 918 | 1238 | 40 | 713 | 4554 | 78% | 527.313 |
| S5213G5 | 5353 | 2377 | 40 | 155 | 40 | 40 | 352 | 40 | 40 | 597 | 224 | 226 | 136 | 1349 | 339 | 40 | 40 | 316 | 1860 | 1366 | 40 | 40 | 13921 | 63% | 769.528 |
| S30349 | 1060 | 1431 | 111 | 885 | 502 | 181 | 1353 | 40 | 40 | 3331 | 100 | 107 | 1007 | 2814 | 161 | 40 | 131 | 2365 | 1887 | 929 | 782 | 179 | 7605 | 90% | 864.588 |
| S30655 | 1973 | 310 | 40 | 1167 | 1414 | 833 | 1291 | 159 | | 17184 | 40 | 40 | 3550 | 1763 | | 40 | 1313 | 40 | 855 | 2933 | 40 | 986 | 2846 | 79% | 1291.24 |
| S50741 | 3026 | 7023 | 40 | 117 | 134 | 40 | 204 | 40 | 40 | 2322 | 467 | 3187 | 40 | 1556 | 1931 | 113 | 40 | 40 | 6025 | 1250 | 379 | | 7883 | 75% | 889.856 |
| S30440 | 2114 | 530 | 560 | 226 | 31059 | 40 | 40 | 2413 | 6853 | 1270 | 209 | 173 | 152 | 131 | 7059 | 12201 | 2759 | 8886 | 1730 | 2904 | 4826 | 1851 | 37204 | 85% | 1443.93 |
| S32212 | 1121 | 978 | 510 | 40 | 2543 | 40 | 40 | 40 | 2141 | 40 | 2023 | 40 | 40 | 143 | 40 | 40 | 166 | 1370 | 255 | 237 | 40 | 188 | 37589 | 56% | 900.858 |
| S32224 | 2404 | 406 | 612 | 286 | 55494 | 40 | 987 | 21376 | 4234 | 348 | 1650 | 577 | 2406 | 443 | 5563 | 20630 | 8094 | 9353 | 2252 | 5563 | 14006 | 2221 | 14844 | 98% | 2301.47 |
| S32615 | 1034 | 259 | 401 | 40 | 1779 | 40 | 40 | 1319 | 2826 | 40 | 248 | 374 | 40 | 135 | 712 | 3354 | 4827 | 3111 | 1131 | 783 | 241 | 7897 | 6708 | 76% | 673.934 |
| S5206G5 | 4701 | 576 | 25479 | 200 | 12580 | 40 | 163 | 1775 | 200000 | 40 | 780 | 1079 | 284 | 630 | 8107 | 148845 | 6757 | 117642 | 7390 | 5508 | 214 | 22996 | 200000 | 83% | 2992.37 |
| S50725 | 1703 | 215 | 40 | 40 | 12850 | 40 | 465 | 4589 | 112044 | 40 | 7498 | 432 | 40 | 883 | 59393 | 9588 | 15805 | 92277 | 31593 | 24993 | 838 | 8919 | 2083875 | 83% | 4388.19 |
| S32505 | 1060 | 134 | 408 | 40 | | 129 | 351 | 284 | 3484 | 40 | 363 | 460 | 40 | 113 | 1306 | 13055 | 1446 | 40 | 2233 | 1239 | 155 | 1234 | 3667 | 82% | 522.732 |
| S51434 | 581 | 861 | 1611 | 1651 | 341 | 425 | 411 | 210 | 644 | 228 | 181 | 811 | 683 | 5942 | 1416 | 5069 | 3110 | 5413 | 929 | 3951 | 6613 | 2218 | 4876 | 100% | 1601.81 |
| S51928 | 400 | 40 | 254 | 40 | 7554 | 40 | 40 | 1755 | 2894 | 104 | 40 | 40 | 40 | 40 | 7717 | 7138 | 5730 | 2460 | 973 | 1336 | 10559 | 399 | 10439 | 68% | 1113.18 |
| S30725 | 108 | 242 | 257 | 40 | 1059 | 40 | 40 | 266 | 40 | 40 | 40 | 425 | 173 | 107 | 859 | 1245 | 820 | 40 | 891 | 113 | 1275 | 738 | 1710 | 73% | 540.205 |
| S30743 | 545 | 3081 | 40 | 40 | 1051 | 40 | 345 | 67 | 142 | 99 | 56 | 81 | 1078 | 222 | 40 | 40 | 2730 | 40 | 1804 | 270 | 116 | 1791 | 8103 | 80% | 402.178 |
| S51603 | 304 | 40 | 40 | 698 | 591 | 830 | 150 | 469 | 40 | 206 | 244 | 430 | 1606 | 614 | 273 | 991 | 793 | 189 | 2662 | 695 | 2339 | 6753 | 7914 | 85% | 780.016 |
| S52354 | 1099 | | 218 | 113 | 38221 | 199 | 40 | 1606 | 40 | 263 | 291 | 2088 | 1116 | 580 | 444 | 672 | 336 | 167 | 1451 | 266 | 105 | 1244 | 834 | 93% | 429.883 |
| S52725 | 1296 | 418 | 227 | 420 | 1501 | 40 | 332 | 1868 | 40 | 105 | 915 | 608 | 208 | 1986 | 513 | 348 | 3215 | 1845 | 4136 | 1443 | 756 | 3034 | 9007 | 93% | 881.016 |
| S50252 | 4704 | 1021 | 225 | 283 | 670 | 517 | 220 | 178 | 118 | 254 | 401 | 331 | 1353 | 752 | 2197 | 3102 | 109 | 507 | 1077 | 1467 | 2312 | 118 | 1235 | 98% | 646.684 |
| S50804 | 40 | 318 | 508 | 40 | | 193 | 225 | 342 | 574 | 258 | 40 | 4426 | 298 | 1822 | 40 | 336 | 1228 | 1069 | 155 | 638 | 349 | 40 | 6683 | 88% | 628.906 |

Spearman correlation coefficients of plasma and reference bnAbs

| Paticipant | Neutralizati | Subtype | Predicted bnAb specificity of donor plasma (defined by MSBP as in Ref. 19) | CD4bs | | | | | | | | | | | | | | | V3-Gly | | | | | | | | | |
|---|---|---|---|---|---|---|---|---|---|---|---|---|---|---|---|---|---|---|---|---|---|---|---|---|---|---|---|---|
| | | | | VRC01 | VRC-PG04 | 3BNC117 | NIH45-46 | N6 | 1NC9 | N49P7 | CH235.12 | 1-18 | VRC.CH31 | BG24 | 8ANC134 | HJ16 | IOMA | b12 | 10_1074 | PGT121 | PGT128 | VRC41.01 | PGDM12 | PGDM21 | PGT125 | PGT130 | PGT135 | 2G12 |
| S10906 | elite | G | CD4bs | 0.53 | 0.31 | 0.52 | 0.44 | 0.46 | 0.34 | 0.49 | 0.35 | 0.71 | 0.49 | 0.53 | 0.41 | -0.10 | -0.03 | -0.20 | 0.11 | 0.19 | 0.21 | 0.18 | 0.06 | 0.02 | 0.21 | 0.12 | 0.25 | 0.13 |
| S30402 | elite | B | CD4bs | 0.70 | 0.45 | 0.67 | 0.72 | 0.56 | 0.51 | 0.74 | 0.70 | 0.75 | 0.68 | 0.77 | 0.63 | 0.10 | 0.27 | 0.12 | 0.00 | 0.15 | 0.18 | 0.03 | 0.01 | 0.12 | 0.29 | 0.15 | 0.18 | 0.27 |
| S31522 | elite | B | CD4bs | 0.58 | 0.35 | 0.60 | 0.59 | 0.55 | 0.38 | 0.71 | 0.59 | 0.65 | 0.63 | 0.66 | 0.51 | -0.06 | 0.21 | 0.07 | -0.05 | 0.08 | 0.12 | -0.03 | -0.07 | 0.07 | 0.20 | 0.14 | 0.17 | 0.23 |
| S50216 | elite | B | CD4bs | 0.26 | 0.29 | 0.47 | 0.35 | 0.15 | 0.32 | 0.37 | 0.26 | 0.42 | 0.25 | 0.55 | 0.09 | -0.05 | 0.41 | 0.29 | 0.00 | -0.04 | 0.32 | 0.08 | 0.10 | 0.10 | 0.30 | -0.01 | 0.18 | 0.34 |
| S50408 | broad | B | CD4bs | 0.08 | 0.15 | 0.35 | 0.09 | -0.01 | 0.29 | 0.27 | 0.21 | 0.25 | 0.15 | 0.16 | 0.22 | -0.03 | 0.37 | 0.01 | 0.18 | 0.09 | 0.27 | 0.10 | 0.22 | 0.09 | 0.26 | 0.25 | 0.14 | 0.28 |
| S50514 | elite | B | CD4bs | 0.49 | 0.41 | 0.44 | 0.52 | 0.35 | 0.37 | 0.58 | 0.35 | 0.68 | 0.42 | 0.55 | 0.28 | 0.05 | 0.25 | -0.05 | 0.33 | 0.15 | 0.27 | 0.22 | 0.41 | 0.14 | 0.03 | -0.13 | 0.39 | 0.37 |
| S50616 | elite | B | CD4bs | 0.41 | 0.24 | 0.46 | 0.36 | 0.45 | 0.30 | 0.56 | 0.51 | 0.44 | 0.38 | 0.59 | 0.33 | -0.06 | 0.24 | 0.23 | -0.28 | -0.18 | -0.12 | -0.21 | -0.27 | -0.17 | 0.06 | 0.07 | -0.14 | 0.07 |
| S50901 | elite | B | CD4bs | 0.44 | 0.33 | 0.41 | 0.41 | 0.42 | 0.27 | 0.58 | 0.37 | 0.49 | 0.43 | 0.64 | 0.44 | -0.09 | 0.32 | 0.09 | -0.04 | 0.04 | 0.02 | -0.12 | -0.14 | -0.06 | 0.11 | -0.01 | 0.02 | 0.05 |
| S52026 | cross | B | CD4bs | 0.38 | 0.56 | 0.45 | 0.47 | 0.35 | 0.41 | 0.45 | 0.45 | 0.41 | 0.32 | 0.42 | 0.21 | 0.05 | 0.27 | 0.13 | 0.42 | 0.43 | 0.52 | 0.37 | 0.45 | 0.28 | 0.29 | 0.22 | 0.29 | 0.24 |
| S52624 | elite | B | CD4bs | 0.61 | 0.42 | 0.62 | 0.59 | 0.47 | 0.53 | 0.64 | 0.60 | 0.77 | 0.62 | 0.69 | 0.61 | 0.02 | 0.41 | 0.05 | 0.06 | 0.17 | 0.27 | 0.06 | 0.07 | 0.12 | 0.35 | 0.17 | 0.27 | 0.34 |
| S30108 | elite | B | V3-Gly | 0.18 | 0.14 | 0.13 | 0.18 | 0.33 | 0.05 | 0.40 | 0.08 | 0.48 | 0.21 | 0.30 | 0.23 | -0.04 | 0.13 | 0.11 | 0.56 | 0.32 | 0.28 | 0.49 | 0.48 | 0.45 | 0.08 | 0.15 | 0.60 | 0.31 |
| S11456 | broad | A | V3-Gly | 0.41 | 0.26 | 0.34 | 0.37 | 0.45 | 0.26 | 0.37 | 0.38 | 0.56 | 0.36 | 0.50 | 0.37 | -0.09 | 0.08 | -0.05 | 0.34 | 0.59 | 0.52 | 0.40 | 0.37 | 0.28 | 0.51 | 0.41 | 0.46 | 0.19 |
| S30755 | broad | B | V3-Gly | 0.39 | 0.48 | 0.51 | 0.34 | 0.08 | 0.42 | 0.30 | 0.31 | 0.43 | 0.39 | 0.27 | 0.28 | -0.06 | 0.42 | -0.02 | 0.39 | 0.30 | 0.44 | 0.22 | 0.43 | 0.32 | 0.35 | 0.12 | 0.23 | 0.51 |
| S32405 | elite | B | V3-Gly | 0.31 | 0.47 | 0.37 | 0.32 | 0.19 | 0.33 | 0.31 | 0.27 | 0.41 | 0.35 | 0.33 | 0.09 | 0.16 | 0.44 | 0.12 | 0.41 | 0.24 | 0.54 | 0.36 | 0.44 | 0.27 | 0.39 | 0.26 | 0.33 | 0.35 |
| S5213G5 | elite | B | V3-Gly | 0.25 | 0.34 | 0.18 | 0.23 | 0.40 | 0.17 | 0.24 | 0.26 | 0.43 | 0.21 | 0.22 | 0.23 | -0.03 | 0.05 | 0.03 | 0.87 | 0.77 | 0.64 | 0.77 | 0.80 | 0.63 | 0.32 | 0.34 | 0.64 | 0.31 |
| S30349 | elite | B | V3-Gly | 0.47 | 0.36 | 0.51 | 0.49 | 0.32 | 0.40 | 0.42 | 0.41 | 0.62 | 0.47 | 0.50 | 0.46 | -0.04 | 0.13 | 0.05 | 0.52 | 0.56 | 0.67 | 0.59 | 0.48 | 0.43 | 0.45 | 0.43 | 0.55 | 0.32 |
| S30655 | broad | B | V3-Gly | 0.00 | -0.03 | 0.13 | -0.01 | -0.13 | 0.03 | -0.06 | -0.02 | 0.23 | 0.07 | 0.05 | 0.05 | 0.18 | 0.06 | 0.09 | 0.52 | 0.34 | 0.77 | 0.58 | 0.54 | 0.43 | 0.58 | 0.54 | 0.59 | 0.45 |
| S50741 | broad | B | V3-Gly | 0.16 | 0.23 | 0.06 | 0.08 | 0.25 | 0.06 | 0.08 | 0.08 | 0.32 | 0.05 | 0.09 | 0.02 | -0.07 | 0.03 | 0.03 | 0.86 | 0.58 | 0.40 | 0.72 | 0.85 | 0.54 | 0.06 | 0.07 | 0.55 | 0.32 |
| S30440 | elite | A | V2-Apex | 0.13 | 0.04 | 0.22 | -0.01 | 0.08 | 0.04 | 0.01 | 0.18 | 0.05 | 0.05 | 0.07 | -0.03 | -0.21 | -0.19 | -0.40 | -0.19 | -0.12 | 0.02 | -0.19 | -0.11 | -0.35 | 0.17 | 0.19 | -0.09 | -0.14 |
| S32212 | elite | G | V2-Apex | 0.41 | 0.36 | 0.33 | 0.36 | 0.25 | 0.26 | 0.29 | 0.21 | 0.43 | 0.29 | 0.32 | 0.28 | -0.17 | -0.02 | -0.25 | -0.12 | -0.03 | 0.10 | -0.03 | -0.08 | -0.29 | 0.16 | 0.16 | 0.10 | -0.15 |
| S32224 | elite | A | V2-Apex | 0.11 | -0.02 | 0.22 | -0.01 | 0.25 | -0.08 | 0.05 | 0.20 | 0.09 | 0.05 | 0.10 | -0.03 | -0.36 | -0.16 | -0.19 | -0.25 | -0.22 | -0.08 | -0.20 | -0.24 | -0.35 | 0.14 | 0.18 | -0.13 | -0.12 |
| S32615 | elite | AE | V2-Apex | 0.11 | 0.11 | 0.13 | -0.01 | 0.16 | 0.10 | 0.12 | 0.15 | 0.09 | 0.00 | 0.11 | -0.08 | -0.18 | -0.10 | -0.26 | -0.23 | -0.17 | -0.09 | -0.31 | -0.21 | -0.41 | 0.05 | 0.13 | -0.16 | -0.22 |
| S5206G5 | elite | D | V2-Apex | 0.07 | 0.15 | 0.12 | -0.04 | 0.07 | 0.11 | 0.02 | 0.13 | 0.05 | 0.00 | 0.07 | -0.09 | -0.08 | -0.06 | -0.22 | -0.17 | -0.03 | 0.03 | -0.28 | -0.16 | -0.36 | 0.18 | 0.17 | -0.19 | -0.11 |
| S50725 | elite | AG | V2-Apex | 0.25 | 0.18 | 0.27 | 0.11 | 0.28 | 0.11 | 0.21 | 0.24 | 0.26 | 0.19 | 0.26 | 0.00 | -0.14 | -0.09 | -0.16 | -0.20 | -0.23 | 0.01 | -0.23 | -0.09 | -0.37 | 0.25 | 0.22 | -0.10 | -0.08 |
| S32505 | elite | D | V2-Apex | 0.05 | 0.04 | -0.01 | -0.08 | 0.12 | -0.04 | 0.06 | 0.07 | 0.00 | -0.04 | 0.10 | -0.19 | -0.02 | -0.12 | -0.03 | -0.05 | -0.12 | -0.12 | -0.15 | -0.03 | -0.22 | -0.01 | 0.00 | -0.10 | -0.16 |
| S51434 | broad | B | V2-Apex | 0.31 | 0.24 | 0.49 | 0.34 | 0.14 | 0.34 | 0.47 | 0.29 | 0.39 | 0.39 | 0.49 | 0.34 | -0.10 | 0.42 | 0.23 | -0.05 | 0.02 | 0.15 | -0.06 | -0.09 | 0.04 | 0.27 | 0.06 | 0.10 | 0.39 |
| S51928 | elite | A | V2-Apex | 0.07 | -0.05 | 0.25 | -0.06 | 0.05 | 0.04 | 0.04 | 0.21 | 0.04 | 0.04 | 0.06 | -0.01 | -0.23 | -0.13 | -0.22 | -0.20 | -0.15 | -0.02 | -0.20 | -0.15 | -0.29 | 0.20 | 0.26 | -0.12 | -0.05 |
| S30725 | elite | B | MPER | -0.11 | -0.18 | 0.07 | -0.15 | -0.04 | -0.01 | 0.05 | -0.04 | 0.03 | -0.13 | 0.03 | -0.03 | -0.12 | 0.02 | 0.14 | -0.02 | -0.12 | -0.07 | -0.03 | -0.02 | 0.00 | 0.05 | 0.07 | -0.04 | 0.18 |
| S30743 | cross | CPX | MPER | 0.14 | 0.17 | 0.26 | 0.08 | 0.06 | 0.19 | 0.03 | 0.18 | 0.25 | 0.04 | 0.10 | -0.09 | 0.16 | -0.06 | 0.07 | 0.39 | 0.25 | 0.48 | 0.45 | 0.30 | 0.27 | 0.36 | 0.42 | 0.26 | 0.13 |
| S51603 | cross | B | MPER | 0.00 | -0.05 | 0.19 | 0.02 | -0.05 | 0.01 | 0.13 | 0.00 | 0.10 | 0.00 | 0.20 | -0.04 | 0.04 | 0.21 | 0.29 | -0.01 | -0.13 | 0.10 | 0.11 | 0.03 | 0.08 | 0.15 | 0.07 | 0.00 | 0.30 |
| S52354 | cross | C | MPER | -0.07 | 0.10 | 0.04 | -0.09 | 0.09 | 0.01 | -0.04 | -0.05 | 0.01 | -0.19 | -0.01 | -0.24 | -0.18 | 0.09 | 0.13 | 0.26 | 0.04 | 0.10 | 0.15 | 0.24 | 0.04 | -0.14 | 0.00 | 0.13 | 0.02 |
| S52725 | elite | B | MPER | 0.23 | 0.14 | 0.38 | 0.15 | 0.25 | 0.16 | 0.30 | 0.23 | 0.40 | 0.22 | 0.30 | 0.08 | -0.16 | 0.19 | -0.02 | 0.00 | -0.06 | 0.09 | -0.01 | 0.02 | 0.00 | 0.23 | 0.19 | 0.08 | 0.15 |
| S50252 | elite | B | IF_FP | 0.09 | 0.07 | 0.22 | 0.14 | 0.20 | 0.01 | 0.23 | 0.21 | 0.19 | 0.02 | 0.21 | 0.11 | 0.01 | -0.06 | 0.12 | 0.30 | 0.24 | 0.22 | 0.31 | 0.34 | 0.17 | 0.06 | 0.10 | 0.16 | 0.27 |
| S50804 | broad | B | SF | 0.22 | 0.16 | 0.23 | 0.08 | 0.32 | 0.00 | 0.22 | 0.12 | 0.28 | 0.26 | 0.15 | 0.33 | -0.27 | 0.25 | -0.08 | 0.10 | 0.12 | 0.21 | 0.01 | -0.14 | -0.02 | 0.43 | 0.47 | 0.17 | 0.17 |

| Paticipant | V2-Apex | | | | | | | MPER | | | | | Interface/Fusion Peptide | | | | | Silent Face |
|---|---|---|---|---|---|---|---|---|---|---|---|---|---|---|---|---|---|---|
| | PG9 | PG16 | PGDM1400 | CAP256-VRC26 | CH01 | PGDM1401 | PGT145 | 4E10 | 10E8 | DH511.11P | PGZL1 | 2F5 | VRC34.01 | PGT151 | 8ANC195 | ACS202 | 35O22 | SF12 |
| S10906 | 0.41 | 0.43 | 0.41 | 0.16 | 0.34 | 0.35 | 0.37 | -0.03 | 0.02 | 0.04 | 0.06 | 0.09 | 0.38 | 0.34 | -0.17 | 0.01 | 0.24 | 0.29 |
| S30402 | 0.01 | 0.06 | 0.04 | -0.31 | 0.06 | 0.09 | 0.20 | 0.16 | 0.11 | 0.06 | 0.03 | 0.34 | 0.13 | 0.17 | 0.26 | 0.13 | 0.22 | 0.29 |
| S31522 | -0.02 | 0.02 | -0.03 | -0.32 | -0.02 | 0.02 | 0.15 | 0.32 | 0.24 | 0.23 | 0.21 | 0.39 | 0.11 | 0.06 | 0.21 | 0.02 | 0.33 | 0.22 |
| S50216 | 0.12 | 0.16 | 0.24 | 0.06 | 0.32 | 0.30 | 0.21 | 0.39 | 0.43 | 0.48 | 0.30 | 0.19 | 0.15 | 0.27 | 0.09 | 0.31 | 0.14 | 0.15 |
| S50408 | 0.05 | 0.03 | 0.09 | -0.04 | 0.14 | 0.13 | 0.23 | 0.06 | 0.24 | 0.05 | 0.08 | 0.04 | 0.09 | 0.16 | 0.29 | 0.16 | 0.20 | 0.35 |
| S50514 | 0.02 | -0.05 | 0.08 | -0.13 | 0.07 | 0.08 | 0.20 | 0.08 | 0.17 | 0.18 | 0.08 | 0.05 | 0.12 | 0.15 | 0.05 | 0.17 | 0.04 | 0.16 |
| S50616 | 0.16 | 0.15 | 0.12 | -0.15 | 0.21 | 0.05 | 0.07 | 0.29 | 0.33 | 0.27 | 0.19 | 0.41 | 0.15 | -0.04 | 0.24 | -0.24 | 0.24 | 0.15 |
| S50901 | 0.19 | 0.19 | 0.21 | -0.07 | 0.22 | 0.13 | 0.20 | 0.39 | 0.36 | 0.32 | 0.21 | 0.50 | 0.21 | 0.06 | 0.00 | -0.03 | 0.26 | 0.33 |
| S52026 | 0.00 | 0.04 | 0.05 | 0.00 | 0.06 | 0.02 | 0.16 | 0.27 | 0.30 | 0.21 | 0.17 | -0.11 | 0.17 | 0.51 | 0.02 | 0.38 | 0.06 | 0.27 |
| S52624 | 0.02 | 0.05 | 0.07 | -0.27 | -0.01 | 0.13 | 0.12 | 0.19 | 0.17 | 0.12 | 0.10 | 0.36 | 0.04 | 0.16 | 0.13 | 0.15 | 0.13 | 0.32 |
| S30108 | -0.15 | -0.12 | -0.08 | -0.24 | -0.12 | -0.16 | -0.01 | 0.37 | 0.36 | 0.42 | 0.36 | 0.30 | -0.04 | 0.11 | 0.00 | 0.09 | 0.27 | 0.07 |
| S11456 | 0.16 | 0.21 | 0.24 | 0.17 | 0.14 | 0.22 | 0.08 | 0.13 | 0.09 | 0.13 | 0.17 | 0.09 | 0.21 | 0.43 | 0.04 | 0.12 | 0.14 | 0.14 |
| S30755 | 0.12 | 0.03 | 0.16 | 0.02 | 0.10 | 0.19 | 0.19 | -0.03 | 0.08 | 0.02 | -0.11 | 0.01 | 0.08 | 0.29 | -0.03 | 0.24 | 0.07 | 0.44 |
| S32405 | -0.04 | -0.01 | 0.12 | -0.02 | 0.11 | 0.11 | 0.17 | 0.41 | 0.44 | 0.40 | 0.30 | 0.27 | 0.08 | 0.39 | 0.11 | 0.32 | 0.16 | 0.32 |
| S5213G5 | -0.03 | -0.05 | 0.01 | 0.06 | 0.02 | -0.13 | -0.02 | 0.04 | 0.02 | 0.05 | 0.07 | -0.16 | 0.18 | 0.40 | -0.17 | 0.23 | 0.11 | 0.04 |
| S30349 | 0.13 | 0.14 | 0.18 | -0.20 | 0.12 | 0.10 | 0.29 | 0.06 | 0.04 | 0.02 | 0.05 | 0.03 | 0.14 | 0.45 | 0.08 | 0.21 | 0.10 | 0.21 |
| S30655 | -0.05 | -0.05 | 0.09 | -0.03 | 0.04 | 0.09 | 0.12 | 0.27 | 0.29 | 0.30 | 0.35 | 0.13 | -0.01 | 0.31 | 0.15 | 0.08 | -0.01 | 0.09 |
| S50741 | -0.02 | -0.04 | 0.00 | 0.05 | 0.07 | -0.10 | 0.02 | 0.04 | 0.15 | 0.13 | 0.13 | -0.08 | 0.17 | 0.38 | -0.27 | 0.19 | 0.07 | 0.07 |
| S30440 | 0.85 | 0.86 | 0.81 | 0.54 | 0.75 | 0.67 | 0.48 | 0.01 | 0.17 | 0.18 | 0.16 | 0.16 | 0.40 | 0.31 | -0.01 | -0.14 | -0.10 | 0.22 |
| S32212 | 0.65 | 0.62 | 0.71 | 0.44 | 0.38 | 0.47 | 0.32 | 0.03 | -0.02 | 0.07 | 0.05 | 0.14 | 0.18 | 0.25 | -0.04 | 0.05 | 0.13 | 0.14 |
| S32224 | 0.82 | 0.75 | 0.63 | 0.40 | 0.66 | 0.45 | 0.32 | 0.16 | 0.31 | 0.30 | 0.32 | 0.19 | 0.39 | 0.25 | -0.14 | -0.25 | 0.10 | 0.10 |
| S32615 | 0.67 | 0.62 | 0.70 | 0.48 | 0.58 | 0.54 | 0.32 | 0.23 | 0.32 | 0.33 | 0.33 | 0.30 | 0.23 | 0.04 | -0.04 | -0.20 | 0.03 | 0.22 |
| S5206G5 | 0.56 | 0.57 | 0.64 | 0.59 | 0.55 | 0.51 | 0.21 | 0.19 | 0.25 | 0.29 | 0.26 | 0.21 | 0.30 | 0.09 | 0.02 | -0.16 | 0.03 | 0.17 |
| S50725 | 0.64 | 0.57 | 0.61 | 0.43 | 0.64 | 0.44 | 0.30 | 0.18 | 0.32 | 0.33 | 0.34 | 0.29 | 0.41 | 0.19 | 0.04 | -0.30 | 0.13 | 0.08 |
| S32505 | 0.45 | 0.40 | 0.43 | 0.53 | 0.52 | 0.26 | 0.18 | 0.42 | 0.43 | 0.41 | 0.44 | 0.25 | 0.27 | -0.01 | 0.03 | -0.26 | 0.18 | 0.00 |
| S51434 | 0.20 | 0.13 | 0.25 | -0.21 | 0.33 | 0.35 | 0.51 | 0.13 | 0.29 | 0.08 | 0.04 | 0.23 | 0.13 | 0.11 | 0.11 | 0.17 | 0.17 | 0.40 |
| S51928 | 0.84 | 0.79 | 0.75 | 0.40 | 0.73 | 0.63 | 0.49 | 0.12 | 0.30 | 0.26 | 0.25 | 0.24 | 0.33 | 0.20 | 0.00 | -0.22 | -0.03 | 0.22 |
| S30725 | 0.34 | 0.24 | 0.34 | -0.04 | 0.30 | 0.34 | 0.40 | 0.39 | 0.58 | 0.44 | 0.44 | 0.37 | -0.01 | 0.14 | -0.02 | -0.11 | 0.12 | 0.25 |
| S30743 | 0.15 | 0.11 | 0.30 | 0.11 | 0.29 | 0.23 | 0.23 | 0.41 | 0.43 | 0.39 | 0.48 | 0.20 | 0.03 | 0.17 | 0.18 | -0.02 | 0.18 | 0.22 |
| S51603 | 0.07 | 0.09 | 0.14 | -0.18 | 0.24 | 0.18 | 0.25 | 0.54 | 0.67 | 0.60 | 0.49 | 0.37 | 0.06 | 0.28 | 0.19 | -0.10 | 0.16 | 0.12 |
| S52354 | 0.03 | -0.02 | 0.00 | 0.05 | 0.19 | -0.13 | -0.12 | 0.32 | 0.38 | 0.40 | 0.37 | 0.04 | -0.01 | 0.13 | -0.17 | -0.01 | -0.08 | -0.04 |
| S52725 | 0.20 | 0.20 | 0.27 | 0.03 | 0.29 | 0.31 | 0.17 | 0.46 | 0.56 | 0.57 | 0.51 | 0.43 | 0.13 | 0.23 | 0.01 | -0.01 | 0.18 | 0.25 |
| S50252 | 0.31 | 0.22 | 0.28 | 0.18 | 0.23 | 0.22 | 0.37 | 0.06 | 0.28 | 0.12 | 0.12 | -0.19 | 0.39 | 0.62 | -0.16 | 0.20 | 0.06 | 0.05 |
| S50804 | 0.36 | 0.31 | 0.33 | -0.02 | 0.20 | 0.25 | 0.37 | 0.21 | 0.32 | 0.20 | 0.23 | 0.42 | 0.05 | 0.08 | -0.02 | -0.08 | 0.14 | 0.53 |
