# Supplementary material for: Rare twin cysteine residues in the HIV-1 envelope variable region 1 link to neutralization escape and breadth development
Source: Cell Host Microbe. Author manuscript; Available in PMC 2026 Sep 29. (PMC13621955; doi:10.1016/j.chom.2026.05.021)
Supplement: Supplementary file 2 [file NIHMS2187495-supplement-Table_S2.pdf]

Table S2 - bnAb-inducer Env panel characteristics

Neutralization sensitivity to bnAbs (50% Inhibitory concentration (IC50) μg/mL

Paticipant	Subtype	Env clone	Year of isolation	Isolation weeks post EDI[1]	GenBank Accession	Extra V1Cys	bnAb Plasma Specificity (see Table S1)	VRC01	VRC-PG04	3BNC117	N6	PGT128	PGDM12	PGDM21	PGT121	PGDM1400	VRC26.25	PG16
S10906	G	S10906-109wk-c19	2011	109	PQ282667	C0	CD4bs	0.8159	0.2495	25.0000	0.0462	25.0000	25.0000	25.0000	25.0000	25.0000	0.0148	25.0000
S30402	B	R282_3.4_cl56	2011	176	MH714324	C2	CD4bs	25.0000	25.0000	25.0000	25.0000	25.0000	25.0000	25.0000	25.0000	25.0000	25.0000	25.0000
S31522	B	S31522-147wk-c20	1995	147	PQ282670	C0	CD4bs	25.0000	25.0000	25.0000	25.0000	25.0000	0.0529	0.0244	0.0050	0.2584	0.4996	0.0454
S50216	B	S50216-677wk-c68	1999	677	PQ282684	C0	CD4bs	25.0000	25.0000	24.1761	25.0000	0.0244	0.0760	2.5829	0.0244	25.0000	25.0000	25.0000
S50408	B	S50408-1083wk-c40	2003	1083	PQ282691	C0	CD4bs	25.0000	25.0000	0.1619	0.0426	25.0000	0.7507	25.0000	25.0000	0.9176	25.0000	0.5730
S50514	B	S50514-891wk-c4	2001	891	PQ282692	C0	CD4bs	17.7573	25.0000	25.0000	25.0000	25.0000	25.0000	14.3757	0.9378	1.9215	25.0000	5.8198
S50616	B	S50616-1071wk-c57	2011	1071	PQ282693	C0	CD4bs	25.0000	25.0000	25.0000	25.0000	0.0244	25.0000	25.0000	25.0000	25.0000	25.0000	25.0000
S50901	B	S50901-909wk-c2	2002	909	PQ282697	C0	CD4bs	25.0000	25.0000	21.4372	1.1646	0.1342	1.5646	24.6354	0.4385	0.3222	25.0000	0.2493
S52026	B	S52026-432wk-c83	1996	432	PQ282687	C0	CD4bs	4.2951	0.4207	0.2308	0.0660	25.0000	25.0000	25.0000	25.0000	0.8330	25.0000	1.7733
S52624	B	S52624-272wk-c6	2001	272	PQ282689	C0	CD4bs	25.0000	0.0244	25.0000	0.0882	25.0000	25.0000	25.0000	25.0000	12.5861	25.0000	1.2233
S30108	B	S30108-217wk-c42	2001	217	PQ282669	C1	V3-Gly	23.6386	22.8348	0.3579	0.2915	3.5307	25.0000	25.0000	25.0000	9.1253	25.0000	25.0000
S11456	A	S11456-244wk-c77	2012	244	PQ282666	C0	V3-Gly	8.4401	25.0000	25.0000	0.0807	23.0134	25.0000	25.0000	25.0000	25.0000	25.0000	0.7093
S30755	B	S30755-540wk-c48	2002	540	PQ282679	C0	V3-Gly	24.8538	25.0000	25.0000	3.4341	25.0000	25.0000	25.0000	25.0000	0.0579	25.0000	0.2860
S32405	B	S32405-230wk-c34	2012	230	PQ282673	C0	V3-Gly	1.4319	4.2492	0.8073	0.8078	25.0000	25.0000	25.0000	25.0000	25.0000	25.0000	25.0000
S5213G5	B	S5213G5-230wk-c27	2003	230	PQ282683	C0	V3-Gly	3.5508	1.9842	25.0000	0.0868	0.0244	0.1867	0.0524	0.0207	25.0000	25.0000	25.0000
S30349	B	T282_3.8_cl82	2012	214	MH714327	C2	V3-Gly	25.0000	25.0000	25.0000	0.7185	0.0244	25.0000	25.0000	25.0000	12.6392	25.0000	3.3535
S30655	B	S30655-204wk-c26	2011	204	PQ282668	C2	V3-Gly	2.8837	1.4155	0.1858	0.0788	25.0000	25.0000	25.0000	25.0000	25.0000	25.0000	25.0000
S50741	B	S50741-847wk-c12	2006	847	PQ282695	C0	V3-Gly	3.7443	5.7849	0.2287	0.1194	25.0000	25.0000	25.0000	25.0000	25.0000	25.0000	25.0000
S30440	A	S30440-173wk-c66	2010	173	PQ282676	C0	V2-Apex	0.2780	0.0244	0.0244	0.0244	25.0000	25.0000	25.0000	25.0000	25.0000	25.0000	25.0000
S32212	G	S32212-164wk-c59	2010	164	PQ282671	C0	V2-Apex	2.6741	0.9774	0.0944	0.4431	0.0244	25.0000	25.0000	25.0000	25.0000	25.0000	25.0000
S32224	A	S32224-174wk-c33	2009	174	PQ282672	C0	V2-Apex	25.0000	25.0000	25.0000	20.3544	25.0000	25.0000	25.0000	25.0000	0.0244	25.0000	25.0000
S32615	AE	S32615-178wk-c33	2008	178	PQ282675	C0	V2-Apex	6.4117	0.7241	25.0000	1.2167	0.0448	25.0000	25.0000	25.0000	25.0000	25.0000	25.0000
S5206G5	D	S5206G5-581wk-c51	2008	581	PQ282686	C2	V2-Apex	1.6076	1.5572	0.2083	0.1244	25.0000	25.0000	25.0000	25.0000	25.0000	25.0000	25.0000
S50725	AG	S50725-295wk-c60	2011	295	PQ282694	C0	V2-Apex	25.0000	25.0000	25.0000	0.1059	25.0000	25.0000	25.0000	25.0000	0.0244	0.0058	25.0000
S32505	D	S32505-230wk-c18	2001	230	PQ282674	C0	V2-Apex	25.0000	25.0000	25.0000	0.4728	25.0000	25.0000	0.8285	6.7880	25.0000	25.0000	25.0000
S51434	B	S51434-865wk-c3	2003	865	PQ282680	C0	V2-Apex	25.0000	25.0000	6.0020	3.3246	25.0000	25.0000	25.0000	25.0000	25.0000	25.0000	25.0000
S51928	A	S51928-278wk-c26	1998	278	PQ282682	C0	V2-Apex	0.0244	0.1016	0.0722	0.0266	0.0244	0.0887	0.0777	0.0880	25.0000	21.2220	25.0000
S30725	B	S30725-597wk-c70	2007	597	PQ282677	C0	MPER	2.1537	0.4085	0.0817	0.0929	0.0244	0.3351	25.0000	0.0169	25.0000	25.0000	25.0000
S30743	CPX	S30743-346wk-c88	2001	346	PQ282678	C2	MPER	0.5863	0.1718	0.1150	0.0824	0.1214	25.0000	25.0000	25.0000	25.0000	25.0000	25.0000
S51603	B	S51603-618wk-c58	1995	618	PQ282681	C0	MPER	23.0811	7.1167	0.8410	1.4335	10.8691	25.0000	25.0000	0.1926	25.0000	25.0000	25.0000
S52354	C	S52354-263wk-c60	2006	263	PQ282688	C0	MPER	25.0000	25.0000	25.0000	25.0000	25.0000	25.0000	25.0000	25.0000	2.8656	25.0000	0.1106
S52725	B	S52725-275wk-c19	2007	275	PQ282690	C0	MPER	12.8638	25.0000	7.6434	1.3847	0.0985	25.0000	25.0000	25.0000	25.0000	25.0000	25.0000
S50252	B	S50252-603wk-c11	2001	603	PQ282685	C0	IF_FP	16.1011	2.9121	0.6029	0.8250	25.0000	25.0000	25.0000	25.0000	25.0000	25.0000	25.0000
S50804	B	S50804-770wk-c17	2000	770	PQ282696	C0	SF	1.7647	6.2387	0.4955	0.3158	25.0000	25.0000	25.0000	25.0000	25.0000	25.0000	25.0000
		Average Isolation year	2005															

Paticipant	PGT145	CH01	DH511.11P	10E8	2F5	35O22	PGT151	SF12	PNGS at key Env sites (N) N156	N160	N332	N334
S10906	25.0000	25.0000	0.0244	0.0352	0.9569	0.1517	25.0000	0.2084	N			N
S30402	25.0000		2.7179	5.0668	25.0000	25.0000	0.0023	0.3656	N			N
S31522	0.0197	25.0000	0.0356	0.0655	0.1947	0.0054	0.0569	6.2846	N	N	N	
S50216	25.0000	25.0000	0.6309	0.2193	1.5806	25.0000	0.0276	0.0340	N		N	
S50408	0.0855	7.6000	0.0244	0.0376	25.0000	25.0000	25.0000	0.0257	N	N	N	
S50514	0.1075	16.5108	0.0431	0.2048	25.0000	25.0000	25.0000	7.1989	N	N	N	
S50616	25.0000	10.9966	3.5333	11.8949	24.2347	25.0000	0.2730	0.2251	N	N	N	
S50901	0.0389	6.6982	0.8216	0.2568	4.6690	25.0000	13.6943	0.0926	N	N	N	
S52026	0.0876	1.0180	0.6301	0.9798	3.0835	25.0000	7.8821	1.4528	N	N	N	
S52624	25.0000	0.1775	0.0465	0.0733	0.8885	0.0998	3.7880	0.4275	N	N		N
S30108	1.0363	25.0000	0.1524	0.4451	25.0000	25.0000	25.0000	0.0780	N	N		
S11456	25.0000	18.0979	3.7413	3.1500	25.0000	0.0357	0.0160	0.0485	N	N		N
S30755	0.0112	25.0000	0.8724	0.5881	3.7727	4.4843	25.0000	25.0000	N	N	N	
S32405	8.8867	25.0000	0.1057	0.3672	1.8674	25.0000	25.0000	25.0000	N	N	N	
S5213G5	25.0000	25.0000	0.0227	0.1947	1.4656	25.0000	3.1337	0.2690	N		N	
S30349	0.0326	1.7091	0.1599	0.0511	25.0000	0.2883	25.0000	1.5433	N	N	N	
S30655	25.0000	25.0000	0.0244	0.2257	1.6201	0.0438	25.0000	0.0244	N		N	
S50741	1.9624	25.0000	0.1702	0.1608	1.9824	13.4468	0.0141	0.1034	N	N		
S30440	25.0000	25.0000	0.1965	4.3480	8.8286	25.0000	0.0136	0.1748	N			N
S32212	25.0000	25.0000	0.4519	1.7645	6.9998	0.0015	0.2545	0.0520	N			N
S32224	2.5408	25.0000	1.1711	0.6699	9.0336	25.0000	0.0169	25.0000	N	N		N
S32615	25.0000	25.0000	0.0285	0.5299	25.0000	0.0088	25.0000	25.0000	N			N
S5206G5	25.0000	25.0000	0.1818	0.6273	4.9869	0.4615	25.0000	0.3145	N	N		
S50725	25.0000	10.3990	0.0707	1.3918	24.7923	25.0000	0.0453	25.0000	N	N		N
S32505	25.0000	25.0000	0.1232	0.1140	2.0873	25.0000	25.0000	0.2526	N	N	N	
S51434	12.5700	25.0000	2.2954	1.9772	11.3862	25.0000	25.0000	0.0244	N	N		
S51928	4.2746	25.0000	0.0424	25.0000	5.8157	3.9850	13.2566	25.0000	N	N	N	
S30725	20.5443	25.0000	0.4157	1.6537	11.5026	25.0000	0.0023	0.1356	N	N	N	
S30743	13.8551	25.0000	6.8092	6.5186	25.0000	23.2643	0.0132	1.3156	N		N	
S51603	12.9881	25.0000	0.7993	0.6766	5.2655	25.0000	25.0000	0.0482	N	N	N	
S52354	1.8884	15.2191	0.1808	1.1696	25.0000	0.0101	3.7212	25.0000	N	N		
S52725	2.9051	25.0000	1.3474	0.6835	25.0000	25.0000	25.0000	0.0407	N	N	N	
S50252	25.0000	25.0000	0.0837	0.0738	1.3068	25.0000	25.0000	0.2331	N	N		
S50804	25.0000	25.0000	0.2919	0.1817	25.0000	0.0007	25.0000	0.4703	N			

Legend
1 EDI: estimated date of infection
2 PNGS: Predicted N glycosylation site

	VRC01	VRC-PG04	3BNC117	N6	PGT128	PGDM12	PGDM21	PGT121	PGDM1400	VRC26.25	PG16	PGT145	CH01	DH511.11P	10E8	2F5	35O22	PGT151	SF12
Median IC50	16.93	14.98	14.54	0.46	25.00	25.00	25.00	25.00	25.00	25.00	25.00	17.20	25.00	0.18	0.56	7.91	25.00	13.48	0.26
Q1	2.73	1.09	0.23	0.09	0.10	25.00	25.00	11.34	4.43	25.00	3.97	1.91	16.51	0.05	0.18	2.01	0.19	0.05	0.08
Q3	25.00	25.00	25.00	2.85	25.00	25.00	25.00	25.00	25.00	25.00	25.00	25.00	25.00	0.82	1.59	25.00	25.00	25.00	5.10
IQR	22.27	23.91	24.77	2.76	24.90	0.00	0.00	13.66	20.57	0.00	21.03	23.09	8.49	0.76	1.40	22.99	24.81	24.95	5.02
Resistant (IC50 >1 μg/mL)	30	25	19	13	23	28	30	26	27	31	28	27	32	7	11	31	23	22	12
Neutralized (IC50 <1 μg/mL)	4	9	15	21	11	6	4	8	7	3	6	7	1	27	23	3	11	12	22

bnAb inducer fingerprint	Number of Envs with IC50 > 1 μg/mL VRC01	VRC-PG04	3BNC117	N6	PGT128	PGDM12	PGDM21	PGT121	PGDM1400	VRC26.25	PG16	PGT145	CH01	DH511.11P	10E8	2F5	35O22	PGT151	SF12
CD4bs	9	7	8	6	7	7	9	6	6	8	7	5	8	2	2	7	7	6	3
V3-Glycan	8	8	4	1	6	7	7	7	7	8	6	6	8	1	1	8	5	6	3
V2-Apex	7	5	5	3	6	8	7	8	7	8	9	9	9	2	5	9	6	5	4
MPER	4	3	2	3	2	4	5	3	5	5	4	5	5	2	3	5	4	3	2
Interface/Fusion Peptide	1	1	0	0	1	1	1	1	1	1	1	1	1	0	0	1	1	1	0
Silent Face	1	1	0	0	1	1	1	1	1	1	1	1	1	0	0	1	0	1	0
