# Supplementary material for: Rare twin cysteine residues in the HIV-1 envelope variable region 1 link to neutralization escape and breadth development
Source: Cell Host Microbe. Author manuscript; Available in PMC 2026 Sep 29. (PMC13621955; doi:10.1016/j.chom.2026.05.021)
Supplement: Supplementary file 3 [file NIHMS2187495-supplement-Table_S6.pdf]

		Spearman correlation to reference bnAbs							
	Participant	S5206G5	S30440	S30834	S30931	S51928	S50725	S5213G5	S10437
	Subtype	D	A	C	A	A	AG	B	A
	Predicted plasma specificity (defined by MSBP as in Rusert et al. 2016)	V2-apex	V2-apex	CD4bs	V2-apex	V2-apex	V2-apex	V3-glycan	MPER
V2-apex	PG9	0.56	0.85	0.31	0.83	0.84	0.64	-0.03	0.04
	PG16	0.57	0.86	0.35	0.81	0.79	0.57	-0.05	0.10
	PGDM1400	0.64	0.81	0.41	0.77	0.75	0.61	0.01	0.18
	CAP256-VRC26.25	0.59	0.54	0.27	0.48	0.40	0.43	0.06	0.11
	CH01	0.55	0.75	0.38	0.74	0.73	0.64	0.02	0.30
	PGDM1401	0.51	0.67	0.30	0.59	0.63	0.44	-0.13	0.16
	PGT145	0.21	0.48	0.26	0.41	0.49	0.30	-0.02	-0.02
V3-glycan	10_1074	-0.17	-0.19	0.13	-0.21	-0.20	-0.20	0.87	0.23
	PGT121	-0.03	-0.12	0.25	-0.15	-0.15	-0.23	0.77	0.21
	PGT128	0.03	0.02	0.36	-0.10	-0.02	0.01	0.64	0.39
	VRC41.01	-0.28	-0.19	0.20	-0.23	-0.20	-0.23	0.77	0.19
	PGDM12	-0.16	-0.11	0.11	-0.17	-0.15	-0.09	0.80	0.21
	PGDM21	-0.36	-0.35	0.01	-0.34	-0.29	-0.37	0.63	0.07
	PGT125	0.18	0.17	0.32	0.12	0.20	0.25	0.32	0.36
	PGT130	0.17	0.19	0.23	0.06	0.26	0.22	0.34	0.24
	PGT135	-0.19	-0.09	0.11	-0.11	-0.12	-0.10	0.64	0.35
	2G12	-0.11	-0.14	0.00	-0.11	-0.05	-0.08	0.31	0.04
CD4bs	VRC01	0.07	0.13	0.25	0.14	0.07	0.25	0.25	0.22
	VRC-PG04	0.15	0.04	0.38	0.07	-0.05	0.18	0.34	0.20
	3BNC117	0.12	0.22	0.48	0.20	0.25	0.27	0.18	0.23
	NIH45-46	-0.04	-0.01	0.24	-0.06	-0.06	0.11	0.23	0.23
	N6	0.07	0.08	0.26	0.04	0.05	0.28	0.40	0.23
	1NC9	0.11	0.04	0.39	-0.01	0.04	0.11	0.17	0.04
	N49P7	0.02	0.01	0.20	-0.01	0.04	0.21	0.24	0.16
	CH235.12	0.13	0.18	0.39	0.10	0.21	0.24	0.26	-0.04
	1-18	0.05	0.05	0.29	0.03	0.04	0.26	0.43	0.39
	VRC.CH31	0.00	0.05	0.17	0.10	0.04	0.19	0.21	0.19
	BG24	0.07	0.07	0.36	0.08	0.06	0.26	0.22	0.27
	8ANC134	-0.09	-0.03	0.06	-0.07	-0.01	0.00	0.23	0.08
	HJ16	-0.08	-0.21	-0.04	-0.31	-0.23	-0.14	-0.03	-0.15
	IOMA	-0.06	-0.19	0.19	-0.07	-0.13	-0.09	0.05	0.20
	b12	-0.22	-0.40	0.10	-0.32	-0.22	-0.16	0.03	-0.16
Interface/Fusion Peptide	VRC34.01	0.30	0.40	0.29	0.38	0.33	0.41	0.18	0.15
	PGT151	0.09	0.31	0.31	0.15	0.20	0.19	0.40	0.32
	8ANC195	0.02	-0.01	-0.03	-0.05	0.00	0.04	-0.17	-0.04
	ACS202	-0.16	-0.14	0.22	-0.24	-0.22	-0.30	0.23	0.03
	35O22	0.03	-0.10	0.09	-0.06	-0.03	0.13	0.11	0.04
MPER	4E10	0.19	0.01	0.19	0.07	0.12	0.18	0.04	0.25
	10E8	0.25	0.17	0.15	0.23	0.30	0.32	0.02	0.22
	DH511.11P	0.29	0.18	0.17	0.25	0.26	0.33	0.05	0.40
	PGZL1	0.26	0.16	0.17	0.20	0.25	0.34	0.07	0.30
	2F5	0.21	0.16	-0.06	0.19	0.24	0.29	-0.16	0.22
SF	SF12	0.17	0.22	0.18	0.11	0.22	0.08	0.04	0.07
